# A new pachypleurosaur from the Early Ladinian Prosanto Formation in the Eastern Alps of Switzerland

**DOI:** 10.1186/s13358-022-00254-2

**Published:** 2022-07-13

**Authors:** Nicole Klein, Heinz Furrer, Iris Ehrbar, Marta Torres Ladeira, Henning Richter, Torsten M. Scheyer

**Affiliations:** 1 Palaeontological Institute and Museum, University of Zurich, Karl Schmid-Strasse 4, CH-8006 Zurich, Switzerland; 2 Diagnostic Imaging Research Unit (DIRU), Clinic for Diagnostic Imaging, Department of Clinical Diagnostics and Services, Vetsuisse Faculty, University of Zurich, Winterthurerstrasse 258c, CH-8057 Zurich, Switzerland

**Keywords:** Alpine Triassic, European pachypleurosaur diversity, Stratigraphic and palaeogeographic distribution, Phylogeny, Character combination, Tooth replacement

## Abstract

The Alpine Prosanto Formation (Middle Triassic) cropping out in the Ducan region in eastern Switzerland has yielded a rich fish and reptile fauna. Here, we present new pachypleurosaur remains from the upper part of the formation (Early Ladinian), similar to the previously known pachypleurosaurs from the Middle Triassic UNESCO World Heritage Site of Monte San Giorgio in southern Switzerland/northern Italy. From these remains, a new pachypleurosaur species, *Prosantosaurus scheffoldi* nov. gen. et spec., is described on the basis of six fairly complete skeletons, one disarticulated specimen and an isolated skull. As is typical for pachypleurosaurs and most other Triassic marine reptiles, the new taxon is based to a large degree on a combination of characters (e.g., nasals articulating broadly with the anterior margins of the prefrontals and lacking posterior processes; postorbitals with rounded anterior processes that articulate with the postfrontals anterolaterally) rather than on many unambiguous autapomorphies, although a few of the latter were found including (1) a premaxilla which is excluded from entering both the external and internal nares and (2) a parietal, which is distinctly longer than wide and carrying distinct anterolaterally angled processes. Phylogenetic relationships of the new taxon are tested within European Pachypleurosauria, revealing that the new species is the sister taxon to a clade including *Serpianosaurus*, *Proneusticosaurus*, and the monophyletic *Neusticosaurus* spp. Mapping of palaeogeographic and stratigraphical distribution of valid European pachypleurosaurs shows that a formerly proposed scenario of migration of pachypleurosaurs from the eastern Palaeotethys during the Olenekian into the Germanic Basin and a subsequent diversification and invasion during the Anisian into the intraplatform basins of the South Alpine realm must be re-assessed. The exceptional preservation and preparation of the Ducan fossils further allow the description of tooth replacement patterns for the first time in a European pachypleurosaur species. The “alveolarization” of replacement teeth, the horizontal replacement pattern, and the subsequent remodelling of the functional alveoli during tooth replacement supports the monophyly of Sauropterygia as discussed before.

**Supplementary Information:**

The online version contains supplementary material available at 10.1186/s13358-022-00254-2.

## Introduction

Pachypleurosauria belongs to Sauropterygia, a diverse group of Mesozoic, secondary aquatic marine reptiles. Within Sauropterygia, Pachypleurosauria traditionally comprised the clade Eosauropterygia together with Nothosauroidea and Pistosauroidea, whereas the latter two were united in a clade Eusauropterygia (Rieppel, 1994, 2000). The Middle Triassic of China (Eastern Tethyan realm), however, yielded a considerable number of new taxa that show a mixture of pachypleurosaur and nothosaur features over the past 20 years, leaving them as eosauropterygians of uncertain phylogenetic affinities. In addition, the monophyly of the classically recognized Nothosauridae (*Nothosaurus* spp. and *Lariosaurus* spp.; Rieppel, 2000) was recently questioned as well, while the monophyly of Pachypleurosauria was supported (e.g., Li & Liu, 2020; Liu et al., 2014). In Shang et al. (2020), describing a new nothosaur, *Brevicaudosaurus jiyangshanensis*, a monophyletic Nothosauridae and Nothosauroidea were recovered, but pachypleurosaur taxa fall into a mostly unresolved grade within Eosauropterygia. In the study of Li and Liu (2020), pachypleurosaurs were recovered as sister group of Nothosauroidea and not of Eusauropterygia as in the traditional hypothesis by Rieppel (2000), which would make Eusauropterygia paraphyletic. In the study of Lin et al. (2021), on the other hand, a monophyletic Pachypleurosauria was recovered as a sister group to Eusauropterygia, confirming the monophyly of these groups as traditionally envisioned.

Pachypleurosauria are documented from shallow marine and near coastal sediments of the Germanic Basin, of the Alpine Triassic, the Iberian Peninsula, and of the eastern Tethyan realm. The oldest evidence of Pachypleurosauria is from the late Early Triassic: *Hanosaurus hupehensis* from the Spathian (lower part of the Jialingjiang Formation; Nanzhang-Yuan’an fauna) of China (Li & Liu, 2020; Rieppel, 1998) and the early Middle Triassic: *Dactylosaurus* sp. from the earliest Anisian of Poland (Kowal-Linka & Bodzioch, 2012, 2017; please note that contra Kowal-Linka & Bodzioch, 2017, we follow Bachmann et al., 2010 here in identifying the age of the Röt Formation [Upper Buntsandstein] as Early Anisian [Aegean] and not as Late Olenekian [Spathian]). From the Triassic of Myanmar two pachypleurosaur specimens have been described but their exact stratigraphic age is very uncertain (San et al., 2019). With a Late Ladinian to possibly Early Carnian age, the Chinese pachypleurosaur *Keichousaurus hui* is considered to be one of the youngest representatives of Pachypleurosauria (Lu et al., 2018; Rieppel, 2000), together with finds on the Iberian Peninsula in the western Tethyan realm (Fortuny et al., 2011).

Contrary to the preservation in black shales yielding hundreds of articulated and complete pachypleurosaurs of the coeval localities of the Alpine Triassic such as Monte San Giorgio and of the localities in the Eastern Tethyan province, the classical Muschelkalk localities of the Germanic Basin yielded mainly isolated bones (e.g., Meyer, 1847; Schröder, 1914; Rieppel, 1987), the exception being the locality of Winterswijk, The Netherlands (summarized in Klein & Sander, 2019). Otherwise, the taxonomic identification of pachypleurosaurs is largely restricted to skull morphology for taxa found in the Germanic Basin (Rieppel & Lin, 1995; Klein, 2009; Rieppel, 2000). Four pachypleurosaur species had been recognized from the Germanic Basin so far: *Dactylosaurus gracilis* from the Early Anisian of Poland; *Anarosaurus heterodontus* from the Early Anisian of Winterswijk, The Netherlands, *A. pumilio* from the Middle Anisian of Central Germany, and *Neusticosaurus pusillus* from the Early Ladinian of Hoheneck (summarized in Rieppel, 2000; Klein, 2009). Isolated bones of *Neusticosaurus* are very common in the so-called ‘Grenzbonebed’ (Upper Muschelkalk to Lower Keuper transition; Middle Ladinian) in various localities of southern Germany (Hagdorn et al., 2015) and nearby Muschelkalk outcrops in France (Corroy, 1928). *Anarosaurus* and *Dactylosaurus* are restricted to the Germanic Basin, but *N. pusillus* is also represented by about 550 complete and fragmentary individuals in the Alpine Triassic, namely from Monte San Giorgio (Sander, 1989). Its occurrence in the Germanic Basin documents a marine transgression (Fraas, 1881; Schoch, 2015) that must have connected the epicontinental realm with the Tethys, allowing a faunal exchange and the immigration of this pachypleurosaur.

The genus *Proneusticosaurus* from the Early Anisian of Poland, consisting of two incomplete postcranial skeletons, was regarded by Volz (1902) as “an ancestor (sensu lato) of *Neusticosaurus*”. Decades later, the skeletons named as *Proneusticosaurus* had been interpreted as likely belonging to the eusauropterygian genus *Cymatosaurus* (Klein, 2010; Rieppel, 1994; Rieppel & Hagdorn, 1997; Sander et al., 2014; Sues, 1987) which is solely known by skulls, whereas in two recent studies the validity of *Proneusticosaurus silesiacus* was supported (Klein & Surmik, 2021; Surmik et al., 2018). Klein and Surmik (2021) found morphological and histological evidence for a phylogenetic relationship closer to pachypleurosaurs, as already suggested by Volz (1902), possibly making it a fifth pachypleurosaur taxon in the Germanic Basin. Although outside Poland only documented by isolated bones, *Proneusticosaurus* was widespread in the Early to Middle Anisian in the Germanic Basin and is also reported from the Vicentinian Alps, northeastern Italy (Klein & Surmik, 2021; Rieppel & Hagdorn, 1997).

The Alpine Triassic, mainly documented by the famous Middle Triassic locality of the UNESCO World Heritage site Monte San Giorgio (Switzerland/Italy), has yielded hundreds of complete pachypleurosaur skeletons since excavations started in 1924 (Carroll & Gaskill, 1985; Peyer, 1932; Rieppel, 1989; Sander, 1989). The stratigraphically oldest taxon, until recently, was *Serpianosaurus mirigiolensis* (Rieppel, 1989). It is known only from the upper part of the Besano Formation (formerly known as the ‘Grenzbitumenzone’, a succession of bituminous shales and dolomites that span the Anisian–Ladinian boundary, e.g., Stockar et al., 2012) and is latest Anisian to earliest Ladinian in age. The stratigraphically younger genus *Neusticosaurus* is found in the Lower Meride Limestone, which is Early Ladinian (241–240 Ma) in age. *N. pusillus* is widespread in the Cava Inferiore beds, whereas the Cava Superiore beds mostly yielded specimens of *N. peyeri* (Sander, 1989). The stratigraphically youngest taxon, *N. edwardsii* (Cornalia, 1854), is mainly preserved in the Cascina beds (Carroll & Gaskill, 1985). *Neusticosaurus pusillus* was also described from the Ladinian Perledo Member of Perledo, Northern Italy, about 25 km east of Monte San Giorgio (Rieppel, 1995) and the Early Ladinian Cunardo Formation cropping out at Valtravaglia, Varese, Italy, only 10 km west of Monte San Giorgio (Lombardo et al., 2005). The latest addition to the Monte San Giorgio pachypleurosaur fauna was *Odoiporosaurus teruzzii* (Renesto et al., 2014), recovered from the middle part of the Besano Formation, which is Late Anisian in age and thus slightly older than *S. mirigiolensis*. The species is known only by a single incomplete specimen, which shares some plesiomorphic characters with *Dactylosaurus gracilis* and *Anarosaurus* spp. explaining its relatively basal position in phylogenetic analyses of pachypleurosaur relationships (Renesto et al., 2014).

Additional Alpine pachypleurosaurs include *Neusticosaurus staubi* from the Prosanto Formation of Canton of Grisons in eastern Switzerland (Kuhn-Schnyder, 1959), *Neusticosaurus toeplitschi* (Nopcsa, 1928) from the Partnach Formation of Austria (Rieppel, 1993), and indeterminate pachypleurosaur material from Middle Triassic strata of Slovenia (Hitij et al., 2010; Rieppel, 1997) and Slovakia (Čerňanský et al., 2018). The holotype of *Neusticosaurus staubi* consists of an incomplete, not fully grown individual which, due to the lack of diagnostic characters is treated as a junior synonym of *N. pusillus* (Rieppel, 2000; Rieppel & Lin, 1995; Sander, 1989)*. Neusticosaurus *(*Psilotrachelosaurus*)* toeplitschi* was originally described from the Northern Calcareous Alps of Austria (Nopcsa, 1928), but the stratigraphical provenance of the holotype specimen is not exactly known, although it is considered as Early Ladinian in age (Rieppel, 1987, 1993). An additional specimen of *N. toeplitschi* was described from the “Lower Ladinian limestones of the Partnach Formation” (Tichy, 1998: p. 519) in Carinthia (Gailtal Alps, Kärnten, Austria), supporting the validity and stratigraphic age of *N. toeplitschi*. Other pachypleurosaur specimens had been found in the Gailtal Alps of Austria (e.g., Tichy, 1998; Zapfe & König, 1980), including new specimens awaiting further scientific examination (Wachtler, 2018).

**Fig. 1:**
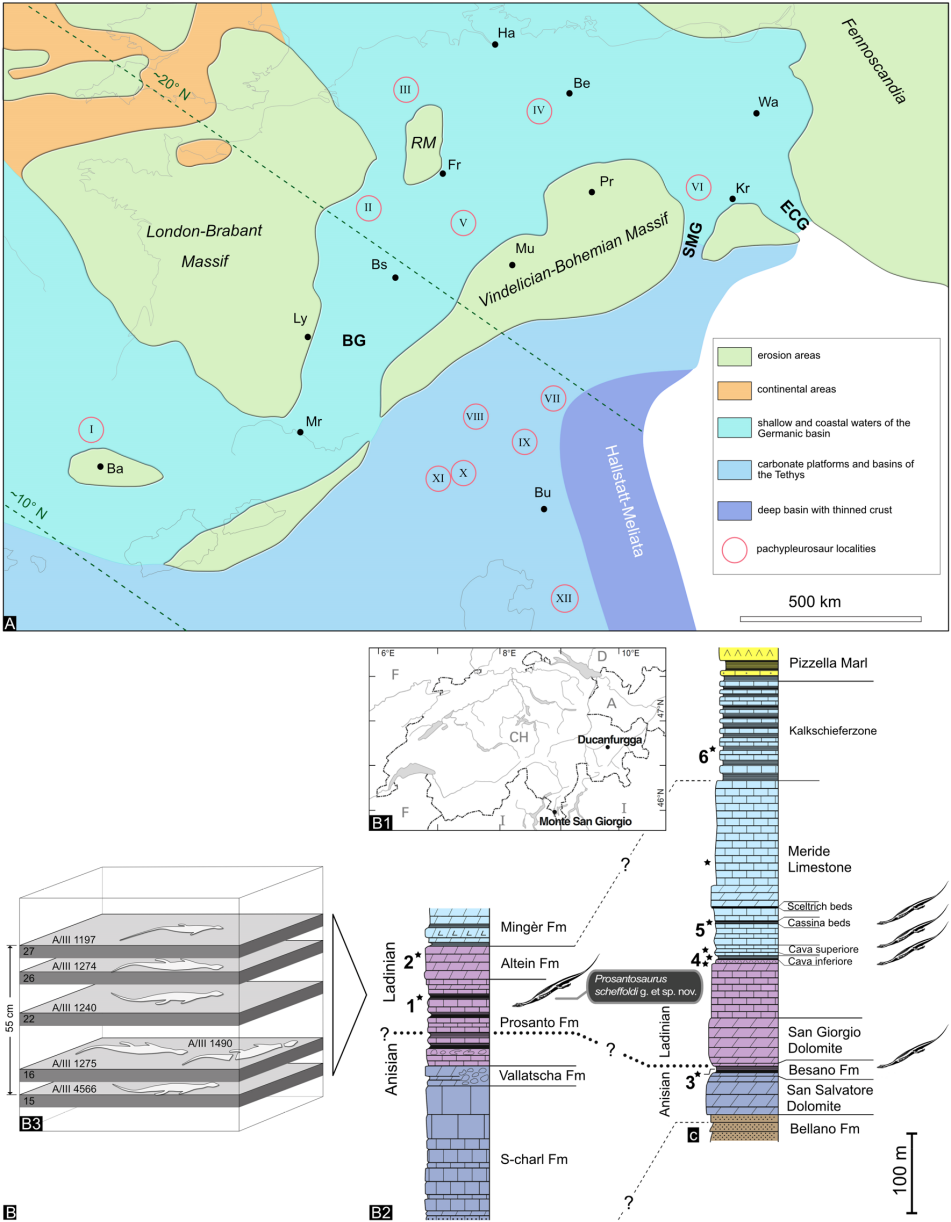
**A** Schematic palaeogeographic map of the Germanic and Alpine Triassic (Middle Triassic) exhibiting European pachypleurosaur occurrences and their stratigraphy. Map is based on Ziegler (2005: fig. 6), Furrer (2019: fig. 129) and references therein. I–VI, Germanic Basin localities; VII–XIII, Alpine Triassic localities. I—Pachypleurosauria indet., Iberian Peninsula (Anisian: Aiguafreda; Ladinian: Mont-ral-Alcover; Carnian: Vilanueva de la Sal); II—*Neusticosaurus* sp., Lorraine region, France (Ladinian); III—*Anarosaurus heterodontus*, Winterswijk, The Netherlands (Early to Middle Anisian); IV—*Anarosaurus pumilio*, Magdeburg, eastern/central Germany (Middle Anisian); V—*Neusticosaurus pusillus*, Hoheneck, southern Germany (Early Ladinian); VI—*Dactylosaurus gracilis*, Gogolin, Poland (Early Anisian); VII—*Neusticosaurus toeplitschi*, Northern Calcareous Alps, Austria (Early Ladinian); VIII—*Prosantosaurus scheffoldi* nov. gen. et sp., Ducan area, SE-Switzerland (Early Ladinian); IX—*Neusticosaurus toeplitschi*, Gailtal Alps, Austria (Early Ladinian); X—*Odoiporosaurus teruzzii*, Besano, Monte San Giorgio area, Italy (Late Anisian); *Serpianosaurus mirigiolensis*, Monte San Giorgio area, Italy/Switzerland (latest Anisian-earliest Ladinian); *Neusticosaurus pusillus*, *N. peyeri* and *N. edwardsii*, Monte San Giorgio, northern Italy/southern Switzerland (Early Ladinian); XI—*Neusticosaurus pusillus*, Valtravaglia, Varese, Italy (Early Ladinian); XII—Pachypleurosauria indet. aff. *Serpianosaurus*/*Neusticosaurus*, West Carpathian Alps, Slovakia (Middle to Late Anisian) and Pachypleurosauria indet., Velika Planina, Slovenia (Early–Middle Anisian). *Ba* Barcelona; *Be* Berlin; *Bs* Basel; *Bu* Budapest; *Fr* Frankfurt a. M.; *Ha* Hamburg; *Kr* Krakow; *Ly* Lyon; *Mr* Marseille; *Mu* Munich; *Pr* Prague; *Wa* Warsaw; *BG* Burgundian Gate; *SMG* Silesian–Moravian Gate; *ECG* Eastern Carpathian Gate. **B** Localities and stratigraphic position of the Alpine pachypleurosaurs in Switzerland. **B1** Map showing the Ducanfurgga locality relative to the UNESCO World Heritage vertebrate site of Monte San Giorgio with the pachypleurosaur-bearing beds indicated. **B2** Correlation of the Middle Triassic section at Ducanfurgga (Upper Austroalpine Silvretta Nappe, south-eastern Switzerland) with that of Monte San Giorgio (southern Alps, southern Switzerland; modified from Scheyer et al., 2017). **B3** Schematic succession of five nearly complete specimens and the disarticulated one found in the upper Prosanto Formation near Ducanfurgga. The numbers refer to bed numbers progressing from older to younger layers

Reviews of the sauropterygian faunal elements from the Iberian Peninsula (Miguel Chaves et al., 2020; Fortuny et al., 2011) showed that pachypleurosaurs, besides nothosaurs, placodonts and pistosaurs, are also found in a variety of localities of Anisian and Ladinian age, with the possible exception of Vilanova de la Sal in Lleida province, which is considered to be Early Carnian in age (Fortuny et al., 2011), although the mentioned “Muschelkalk facies” would likely still indicate a Late Ladinian age instead. The Iberian pachypleurosaurs are yet to be described in detail and currently remain identified as Pachypleurosauria indet. (Miguel Chaves et al., 2020). Rieppel et al. (1999) also mentioned possible isolated postcranial material of pachypleurosaurs from the Middle Triassic Muschelkalk of Makhtesh Ramon, Negev, Israel. See also Fig. 1A for a palaeogeographic and stratigraphical distribution of European Pachypleurosauria.

From the Eastern Tethyan realm, five taxa had been assigned to Pachypleurosauria, of which four are known only by few or only one specimen so far. The oldest representative is the medium-sized *Hanosaurus* from the Nanzhang-Yuan’an fauna (Early Triassic) (Rieppel, 1998). It was found in the Jialingjiang Formation that is today considered to be Spathian in age. It was first described by Young (1972) and is still only known by one specimen preserved on two slabs (Rieppel, 1998). *Dianopachysaurus dingi* from the Anisian of Luoping County, Yunnan Province (Liu et al., 2011) consists of a single, nearly complete and articulated specimen. In spite of its small size (~ 20 cm), the authors argued for a mature ontogenetic stage. *Panzhousaurus rotundirostris* is also a small-sized (less than 50 cm) pachypleurosaur so far known by two specimens (Jiang et al., 2019; Lin et al., 2021) from the Middle Anisian of Panzhou (Guizhou Province, southwestern China). Recently, another small-sized pachypleurosaur, *Honghesaurus longicaudalis* from the Middle Anisian (Pelsonian) of China (Luxi, Honghe, Yunnan) was described on the basis of a single complete specimen (Xu et al., 2022). *Keichousaurus hui* (Young, 1958) represents the so far stratigraphically youngest and best known pachypleurosaur from China. It is known from thousands of individuals recovered from the Zhuganpo Member of the Falang Formation (Late Ladinian or Early Carnian, see above) in a wide geographical area ranging from Dingxiao and Wusha (Xingyi County, Guizhou Province) to Fuyuan County (Yunnan Province) (Benton et al., 2014; Li, 2006).

A current phylogenetic analysis found *Panzhousaurus* most closely related to *Dianopachysaurus* and *Keichousaurus* within Pachypleurosauridae, with *Hanosaurus* forming the sister group to all other pachypleurosaurs (Lin et al., 2021). In the same study, several taxa of hitherto unclear taxonomic affinities among Eosauropterygia had been identified as pachypleurosaurs as well (Lin et al., 2021). These are the medium-sized *Majiashanosaurus discocoracoidis* from the Olenekian (Jiang et al., 2014), the small-sized (less than 50 cm) *Dianmeisaurus gracilis* (Shang & Li, 2015a, 2015b; Shang et al., 2017), *Diandongosaurus acutidentatus* (Sato et al., 2014; Shang et al., 2011), the specialized filter feeder *Wumengosaurus delicatomandibularis* (Cheng et al., 2012; Jiang et al., 2008), all three from the Middle Anisian (Shang & Li, 2015a, 2015b; Shang et al., 2017), and *Qianxisaurus chajiangensis* from the Ladinian (Cheng et al., 2012). *Dawazisaurus brevis* described by Cheng et al. (2016) is likely a junior synonym of *Dianopachysaurus dingi* (Lin et al., 2021). These taxa are again all known only by very few specimens, which provide limited morphological information for each species.

As such, intraspecific variation is known only for a limited number of pachypleurosaurs, namely those species for which high numbers of individuals are known. Ontogenetic variation and sexual dimorphism are well documented among the *Neusticosaurus* species from Monte San Giorgio, *Serpianosaurus mirigiolensis*, and *Keichousaurus hui* (Cheng et al., 2009; Lin & Rieppel, 1998; Rieppel, 1989; Sander, 1989). For *Neusticosaurus*, viviparity or ovoviviparity is inferred (Sander, 1988). Life bearing is documented for *Keichousaurus* (Cheng et al., 2004), and is implicated for other pachypleurosaurs based on their growth record and inferred life history data (Griebeler & Klein, 2019).

Here, we describe a new pachypleurosaur from the upper part of the Prosanto Formation (Early Ladinian) of south-eastern Switzerland (Alpine Triassic). The new taxon occurs almost contemporaneously with Monte San Giorgio pachypleurosaurs (genera *Odoiporosaurus*, *Serpianosaurus,* and *Neusticosaurus*) and thus its phylogenetic relationships are tested foremost among diagnostic European pachypleurosaur taxa. Furthermore, a well-preserved skull from the upper Prosanto Formation allows insights into tooth replacement of European pachypleurosaurs for the first time.

## Geology, stratigraphy and palaeontology of the Prosanto Formation

The Ducan region near Davos in south-eastern Switzerland (Canton of Grisons) belongs to the Austroalpine Silvretta nappe and with the Prosanto Formation provides an important site for Middle Triassic vertebrate fossils (e.g., Bürgin et al., 1991; Furrer, 1995), which has been excavated systematically under supervision of one of us (HF) since 1996 (Furrer, 2009). The fossil record of the formation includes mainly actinopterygian fish and small marine reptiles, but also invertebrates and plant remains (an overview can be found in Furrer, 2019), with few land-living organisms being washed in from nearby islands during storms (Furrer, 2009). The organisms lived in an intraplatform basin, which was separated from the open ocean of the Palaeotethys and became embedded under relatively calm and probably anoxic conditions (Furrer, 1995; Fig. 1). Today, the Ducan region lies about 100 km northeast of the UNESCO site Monte San Giorgio and during the Middle Triassic (Fig. 1B1), both sites were roughly 200 km away from each other (Furrer, 2019). The upper dark lime- and marlstones of the Prosanto Formation with the richest vertebrate fauna were dated to 240.91 ± 0.26 million years using U–Pb zircon ages from a volcanoclastic layer (Furrer et al., 2008), thus placing the here described skeletons into the earliest Ladinian (Fig. 1B2).

## Material and methods

**Fig. 2:**
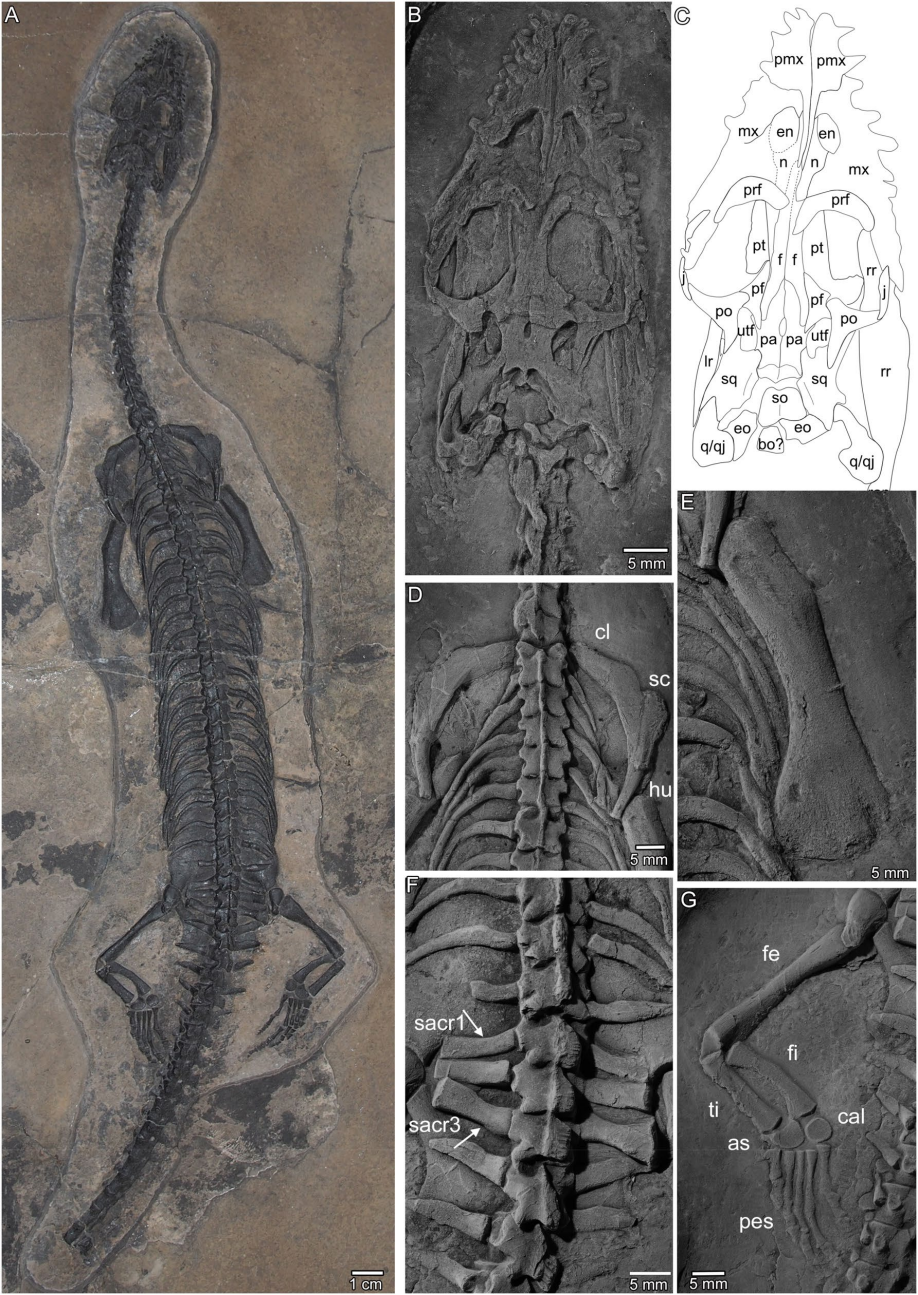
Holotype specimen (PIMUZ A/III 1274) of *Prosantosaurus scheffoldi* gen. et spec. nov. from the upper Prosanto Formation (Early Ladinian, Middle Triassic) of Ducanfurgga locality no. 4, southwest of Davos, Canton of Grisons, south-eastern Switzerland. **A** Nearly complete specimen as prepared in dorsal view. The posterior part of the tail was lost prior to burial. Both forearms are not visible but lie below the trunk region, pointing in an anteromedial direction (see Additional file 1: Fig. S3A). **B** Detail of skull and anterior neck region. **C** Outline sketch of skull sutures. **D** Detail of shoulder girdle (claviculae, scapulae) and anterior dorsal vertebrae and ribs. **E** Detail of right humerus. **F** Detail of posterior dorsal vertebrae and ribs, sacral vertebrae and ribs, and anterior caudal vertebrae and ribs. **G** Detail of left ilium and hindlimb. *ar* articular; *as* astragalus; *bo* basioccipital; *cal* calcaneus; *cl* clavicula; *co* coracoid; *d* dentary; *en* external naris; *eo *exoccipital; *fe* femur; *fi* fibula; *fr* frontal; *hu* humerus; *il* ilium; *in* internal naris; *is* ischium; *j* jugal; *mx* maxilla; *na* naris; *o* orbit; *pa* parietal; *pl* palatine; *pmx* premaxilla; *pof* postfrontal; *po* postorbital; *prf* prefrontal; *pt* pterygoid; *pu* pubis; *q* quadrate; *qj* quadratojugal; *ti* tibia; *sacr* sacral rib; *sc* scapula; *so* supraoccipital; *sp* splenial; *sq* squamosal; *su* surangular; *utf* upper temporal fenestra; *v* vomer

**Fig. 3:**
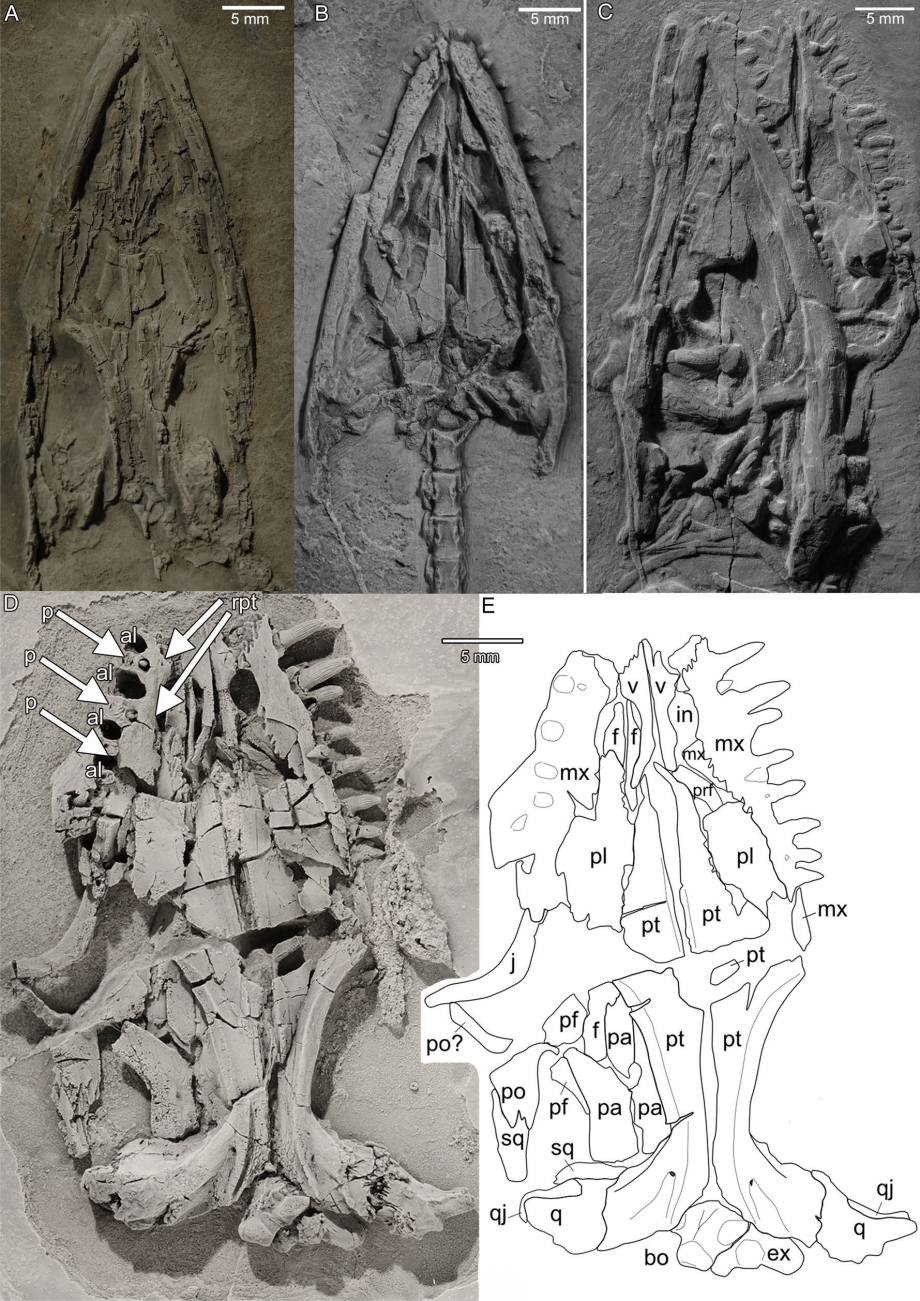
Ventral skull morphology and dentition of *Prosantosaurus scheffoldi* gen et spec. nov. from the upper Prosanto Formation (Early Ladinian, Middle Triassic) of Ducanfurgga locality no. 4, southwest of Davos, Canton of Grisons, south-eastern Switzerland. **A** Detail of the skull in ventral view of specimen PIMUZ A/III 4566. **B** Detail of skull in ventral view of specimen PIMUZ A/III 1275. Note that the posterior pterygoid region is displaced. **C** Detail of lower jaws, skull and dentition in ventral view of specimen PIMUZ A/III 1197. **D** Skull of PIMUZ A/III 1490 providing details of tooth morphology and replacement pattern. **E** Outline sketch of skull PIMUZ A/III 1490. See Additional file 1: Figs. S8, S13 for interpretative and labelled sketches of the skulls of PIMUZ A/III 1275 and PIMUZ A/III 1197

For most specimens, the detailed lithostratigraphic position and taphonomy are documented and the six most complete specimens were found in a bed-by-bed succession in the upper part of the Prosanto Formation (Furrer, 2019: fig. 120) (Fig. 1B3). The fossils were prepared according to standard preparation techniques using pneumatic tools and in the case of PIMUZ A/III 1490, formic acid preparation was employed. Additional figures from all here mentioned specimens (Table 1) are available in the Additional file 1: Figs. S1–S27. An X-ray of the holotype (PIMUZ 1274) was taken at the Diagnostic Imaging Research Unit (DIRU), Clinic for Diagnostic Imaging, Department of Clinical Diagnostics and Services, Vetsuisse Faculty, University of Zurich. Radiography was conducted to check for the presence of the lower arms, which indeed could be identified via X-ray pictures below the anterior trunk region (Additional file 1: Figs. S2, S3). The slab was imaged in dorsoventral orientation in an angle of 90° (57 kV, 10.0 mAs, 30.2 ms) and in an angle of 45° (55 kV, 10.0 mAs, 31.6 ms). The size of the cassette (Fuji IP Casette, Fujifilm, Juni Photo Film Co., Ltd., Japan) was 24 × 30 cm or 18 × 24 cm, respectively. The film-focus-distance (FFD) was set at 120 cm. Specimens were also coated with ammonium chloride for better photographic documentation (Figs. 2, 3, 5; Additional file 1: Figs. S1–S27).

**Table 1:** List of pachypleurosaur specimens from the Prosanto Formation, southwest of Davos, Canton of Grisons, south-eastern Switzerland

Taxonomic assignment	Specimen	Locality	Material	References	Figures
*Prosantosaurus scheffoldi * gen. et spec. nov.	PIMUZ A/III 1274 holotype	Ducanfurgga 4, Davos Sertig	Nearly complete skeleton in dorsal view (both lower forelimbs are covered by the trunk region; posterior part of tail are missing)	Unpublished; PIMUZ excavation 2005	Figure 2; Additional file 1: Figs. S1–S5
*Prosantosaurus scheffoldi * gen. et spec. nov.	PIMUZ A/III 668	Ducantal-Chachlengstell, Davos Sertig	Incomplete skeleton in dorsal view (parts of right body half and posterior tail are missing)	Unpublished; find in scree 1990	Figure 5C; Additional file 1: Fig. S6
*Prosantosaurus scheffoldi * gen. et spec. nov.	PIMUZ A/III 1197	Ducanfurgga 4, Davos Sertig	Nearly complete skeleton in dorsolateral view, skull in ventral view, tail complete	Furrer (2019) *Neusticosaurus staubi*; PIMUZ excavation 1998	Figure 3D; Additional file 1: Figs. S7, S8
*Prosantosaurus scheffoldi * gen. et spec. nov.	PIMUZ A/III 1240	Ducanfurgga 4, Davos Sertig	Nearly complete skeleton in dorsal view (tip of snout and tip of tail missing)	Unpublished; PIMUZ excavation 2000	Figure 5A; Additional file 1: Fig. S9
*Prosantosaurus scheffoldi * gen. et spec. nov.	PIMUZ A/III 4566	Ducanfurgga 4, Davos Sertig	Complete skeleton in ventral view, partly disarticulated	Furrer (2019) *Neusticosaurus staubi*; PIMUZ excavation 1995	Figures 3A, B, 5D, G; Additional file 1: Fig. S10
*Prosantosaurus scheffoldi * gen. et spec. nov.	PIMUZ A/III 1275	Ducanfurgga 4, Davos Sertig	Nearly complete skeleton in ventral view (posterior part of tail is missing)	Unpublished; PIMUZ excavation 2005	Figures 3C, 5B, H; Additional file 1: Figs. S11–S13
*Prosantosaurus scheffoldi * gen. et spec. nov.	PIMUZ A/III 1490	Ducanfurgga 4, Davos Sertig	Incomplete skull in ventral view with disarticulated postcranial material distributed over four slabs	Furrer (2019) *Neusticosaurus staubi*; PIMUZ excavation 1997	Figure 3D, E; Additional file 1: Figs. S14–S16
*Prosantosaurus scheffoldi * gen. et spec. nov.	PIMUZ A/III 710	Ducantal-Hungerbüel, Davos Sertig	Incomplete skull in dorsal view	Bürgin et al. (1991) *Neusticosaurus* cf. *pusillus*; find in scree 1989	Additional file 1: Fig. S17
Pachypleurosauria indet.	PIMUZ A/III 721	Gletscher Ducan, Bergün Stugl	Poorly preserved skull with the anterior cervicals articulated	Unpublished; find in scree 1991	Additional file 1: Fig. S18
Pachypleurosauria indet.	PIMUZ A/III 254	Stulseralp/Val da Stugl, Bergün Stugl	Incomplete trunk region in dorsal view; likely a not fully grown individual	Kuhn-Schnyder (1959) holotype of “*Pachypleurosaurus staubi*”; Carroll and Gaskill (1985)	Additional file 1: Fig. S19
Pachypleurosauria indet.	PIMUZ A/III 711	Ducanfurgga 1, Davos Sertig	Left trunk region in dorsal view	Bürgin et al. (1991) *Neusticosaurus* sp. find in scree 1990	Additional file 1: Fig. S20
Pachypleurosauria indet.	PIMUZ A/III 499	Ducantal, Davos Sertig	Incomplete trunk region in dorsal view	Kuhn-Schnyder (1952) *Pachypleurosaurus* indet. Find in scree before 1952	Additional file 1: Fig. S21
Pachypleurosauria indet.	PIMUZ A/III 720	Ducantal-Chachlengstell, Davos Sertig	Incomplete trunk region in dorsal view	Unpublished; find in scree 1991	Additional file 1: Fig. S22
Pachypleurosauria indet.	PIMUZ A/I 3579	Ducanfurgga 4, Davos Sertig	Disarticulated postcranial elements as stomach content in *Saurichthys* sp.	Furrer (2015, 2019) *Neusticosaurus* sp.; PIMUZ excavation 1998	Additional file 1: Fig. S23

For the phylogenetic analysis every taxon was coded based on personal observation in the following collections: BGR and GPIT (*Dactylosaurus gracilis*), University of Leipzig (*Anarosaurus pumilio*), PIMUZ (*Serpianosaurus mirigiolensis*, *Neusticosaurus pusillus*, *N. peyeri*, *N. edwardsii*; *Prosantosaurus scheffoldi* gen. et spec. nov.), SMNS (*Simosaurus gaillardoti*), TWE and Naturalis (*Anarosaurus heterodontus*), Wuhan Institute of Geology and Mineral Resources (*Keichousaurus hui*). The work of Rieppel (1993) and Tichy (1998) where consulted to code *N. toeplitschi* and that of Renesto et al. (2014) for *Odoiporosaurus teruzzii*. The coding of *Simosaurus*, which was used as outgroup, follows the description of Rieppel (1994) and personal observations at SMNS specimens.

The here used character matrix (Additional file 1: II) is based on a combination of the character matrices of Rieppel and Lin (1995), Li and Liu (2020), Lin et al. (2021), as well as some new characters that had been added (Additional file 1: III). For the present study, mainly characters were chosen that distinguish pachypleurosaur taxa. However, identifying characters that clearly separate pachypleurosaur taxa is difficult due to high interspecific variability, sexual dimorphism, ontogenetic variation, as well as due to various levels of preservation. The analysis is restricted to European Pachypleurosauria herein because (1) the description of the new taxon is studied in the framework of all other known European pachypleurosaurs; (2) the European taxa could be studied first hand in detail; and (3) most non-European taxa are based only on rare material thus limiting the amount of data on intraspecific variation. Please see the additional file for the complete description of characters (Additional file 1: II) and the data matrix (Additional file 1: III). After the initial work of Rieppel and Lin (1995), the three species *Anarosaurus pumilio*, *A. heterodontus* and *Dactylosaurus gracilis,* as well as the four species from Monte San Giorgio (*Neusticosaurus pusillus*, *N. peyeri*, *N. edwardsii*, and *Serpianosaurus mirigiolensis*) had been usually combined in phylogenetic analyses into an *Anarosaurus–Dactylosaurus* and *Serpianosaurus–Neusticosaurus* group, respectively (e.g., Jiang et al., 2014; Liu et al., 2011; Rieppel et al., 2002; Sato et al., 2014; Shang et al., 2017). Only in few recent phylogenetic analyses these taxa had been coded separately again (Li & Liu, 2020; Lin et al., 2021; Renesto et al., 2014). Here, we also code each species separately because phylogenetic relationships of European pachypleurosaurs lie in the focus of the present contribution.

### Systematic palaeontology

**Sauropterygia** Owen, 1860* sensu *Rieppel, 2000

**Eosauropterygia** Rieppel, 1994

**Pachypleurosauria **Nopcsa, 1928 (as diagnosed in Liu et al., 2011**)**

***Prosantosaurus*** ***scheffoldi*** **gen. et spec. nov.**

Figures 2, 3, 4, 5; Tables 1, 2; Additional file 1: Figs. S1–S27.

**Fig. 4:**
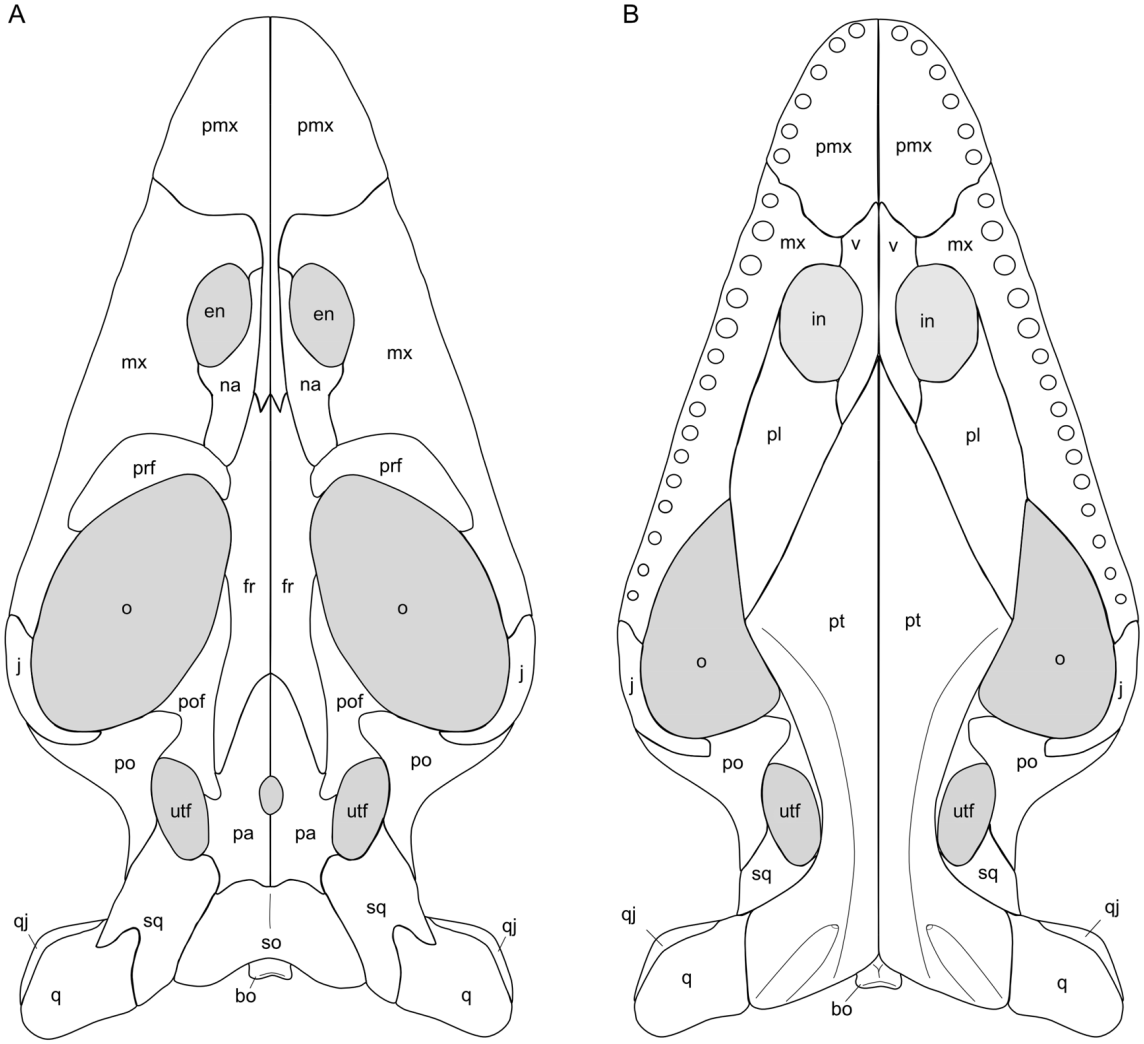
Idealized schematic sketches of the skull of *Prosantosaurus scheffoldi* gen et spec. nov. from the upper Prosanto Formation (Early Ladinian, Middle Triassic) of Ducanfurgga locality no. 4, southwest of Davos, Canton of Grisons, south-eastern Switzerland. **A** Skull in dorsal view and **B** skull in ventral view

**Fig. 5:**
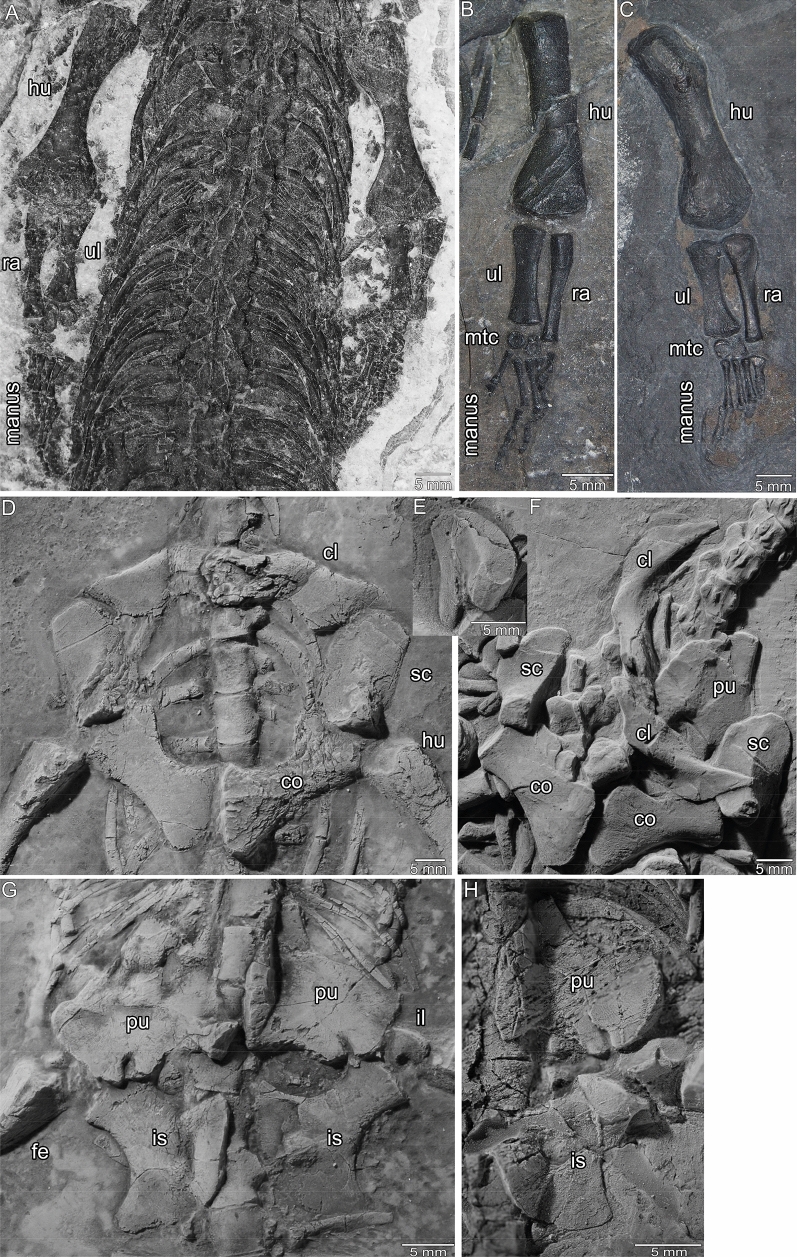
Details of forelimbs and girdle elements of *Prosantosaurus scheffoldi* gen et spec. nov. from the upper Prosanto Formation (Early Ladinian, Middle Triassic) of Ducanfurgga locality no. 4, southwest of Davos, Canton of Grisons, south-eastern Switzerland. **A** Both forearms and anterior trunk region in dorsal view (PIMUZ A/III 1240). **B** Left forearm in ventral view (PIMUZ A/III 1275). **C** Right forearm in dorsal view (PIMUZ A/III 668). **D** Shoulder girdle in ventral view (PIMUZ A/III 4566). **E** Right scapula in ventral view (PIMUZ A/III 1275). **F** Partially disarticulated pelvic girdle in ventral view (PIMUZ A/III 1197). **G** Pelvic girdle in ventral view (PIMUZ A/III 4566). **H** Left pelvic girdle in ventral view (PIMUZ A/III 1275)

**Table 2:** Measurements (in mm) of *Prosantosaurus scheffold*i gen. et spec. nov from the Prosanto Formation (Early Ladinian), southwest of Davos, Canton of Grisons, south-eastern Switzerland

Specimen	PIMUZ A/III 1274	PIMUZ A/III 668	PIMUZ A/III 1240	PIMUZ A/III 1197	PIMUZ A/III 4566	PIMUZ A/III 1275
Body length as preserved	> 450	> 395	> 490	~ 570	> 290	> 300
Condyl. skull length	48	43.5	~ 50	~ 47	45.2	29.6
Length lower jaw	58.5	nm	nm	51.2	48.7	3.8
Neck length	90	72.7	82	nm	82	66
Trunk length (shoulder incl. sacrals)	180	170	~ 170	~ 125	120	97.5
Humerus	38.4/38.2	31.3/30.3	31.9/31.9	34/34.2	23/23.7	20.4/20
Radius	nm	16.7/15.7	16.7/15.1	17.7/18.4	12.55/12.8	10.7/10
Ulna	nm	14.9/13.8	14.8/14	16.1/nm	11.5/11.8	92/88
Femur	30/30.8	27.5	28.4/28.3	nm/31.5	26.3/25.8	22.1/23
Tibia	16/16.7	15.7/nm	15.2/15	15.4/13.4	13/nm	10.6/nm
Fibula	17.9/16.7	17.3/nm	16.1/16.8	13.7/14.5	14/nm	10.9/nm
Number cervicals	18	< 14–15	15	> 11	17	18
Number of dorsals	24	24	23	nm	23–24	25
Number of sacrals	3	3	3	nm	3	3
Number of caudals	> 17 +	> 14 +	> 35 +	60	> 25 +	> 11 +
Number of carpals		2	3	2	2	2
Number of tarsals	2	2	2	2	2	2

### Etymology

The genus is named after the geological unit, the Prosanto Formation, the fossiliferous Lagerstätte in the Ducan mountains southwest of Davos, eastern Switzerland and the Greek term *sauros* for lizard. The species name is in honour of Beat Scheffold, scientific illustrator and sculptor as free-lancer, and technician at PIMUZ, Zurich, Switzerland, for his manifold contributions to Triassic marine reptile research and visualization.

### Differential diagnosis for genus and species

*Prosantosaurus scheffoldi* gen. et spec. nov. is a medium-sized pachypleurosaur with the following unique character combination in its skull morphology: premaxilla excluded from contact with the external and internal nares; small (reduced) parietal that is longer than wide; parietal with narrow posterior shelf, centrally situated parietal foramen, and distinct, anterolaterally angled process accommodating the posterior process of the postfrontal in a triangular facet; posterior skull margin is deeply emarginated due to a far posterior extension of the squamosal to the posterolateral margin of the parietal; constricted temporal region; pterygoid with triangular anterior shelf tapering anteriorly, broad mid-part region, and strongly waisted posterior end with massive lateral extending posterior processes. The new taxon is further distinguishable by the combination of the following postcranial characters: proximally and distally equally broadened sacral ribs with a narrower shaft; gastralia composed of five elements; no pachyostosis in ribs or any other elements.

According to the results of our phylogenetic analyses, the new taxon thus differs by the combination of the following characters from all other European pachypleurosaurs: by the lack of a distinct anterolateral process(es) entering between the prefrontal and the nasal (character 10); the exclusion of the premaxilla from contact with the internal and external naris (character 12); a straight lateral edge of the frontal (character 15); a ventral process of the postfrontal that encompasses the dorsal tip of the postorbital at the dorsoposterior margin of the orbit (character 16); a broad and rounded anteromedial process of the postorbital that overlies the postfrontal (character 17); an anteromedial process of the postorbital that articulates on the postfrontal anterolaterally (character 18); the maxillary tooth row extending to or over the posterior orbit (character 31), and by a subequal length of the preorbital and postorbital regions of skull (character 76).

### Holotype

PIMUZ A/III 1274, a nearly complete and articulated specimen preserved in dorsal view, lacking the posterior half of the tail (Fig. 2; Additional file 1: Figs. S1–S5). Both disarticulated lower forearms are present, but covered by the dorsal vertebrae and ribs and thus only very incompletely visible in dorsal view (Fig. 2A) but visible in X-rays (Additional file 1: Fig. S3A).

### Horizon and locality

Upper Prosanto Formation, 241 Ma, Early Ladinian, Middle Triassic of Ducanfurgga locality no. 4, southwest of Davos, Canton of Grisons, eastern Switzerland.

### Referred specimens

PIMUZ A/III 668, an articulated specimen, which lacks part of the posterior right trunk and hind limb and posterior part of tail; PIMUZ A/III 710, an incomplete skull (Additional file 1: Fig. S17; formerly referred to as “*Neusticosaurus* cf. *pusillus* (Fraas)” and figured by Bürgin et al., 1991: pp. 972–973), PIMUZ A/III 1197, a complete but strongly twisted skeleton; PIMUZ A/III 1240, an articulated specimen lacking the anterior snout and the distal part of the tail; PIMUZ A/III 1275, an articulated specimen lacking the posterior part of the tail; PIMUZ A/III 1490, a disarticulated specimen with well-preserved skull prepared in ventral view, PIMUZ A/III 4566, a mostly complete skeleton with twisted neck, disarticulated right hand and lower limb and disarticulated anterior part of the tail. Figure 1B shows the succession of the beds and embedding of the specimens. The specimens PIMUZ A/III 1197, 1240, 1275, 1490, 4566 were recovered together with the holotype PIMUZ A/III 1274 in a sedimentary sequence of ca. 55 cm thickness, at about 125 cm below the radiometric dated volcanoclastic layer; specimens PIMUZ A/III 668 and 710 were found in the talus from two other outcrops of the upper Prosanto Formation.

### Registration in ZooBank

#### Nomenclatural acts

This published work and the nomenclatural act it contains have been registered in ZooBank, the proposed online registration system for the International Code of Zoological Nomenclature (ICZN). The ZooBank LSIDs (Life Science Identifiers) can be resolved and the associated information viewed through any standard web browser by appending the LSID to the prefix ‘http://zoobank.org/’. The LSID for this publication is: urn:lsid:zoobank.org:pub:FD0E0A35-95C2-4DCC-9303-FA53C105A49B.

### Pachypleurosauria indet.

Because of their incomplete nature, the following pachypleurosaur specimens from the Prosanto Formation (see also Table 1) are considered not diagnostic, and thus cannot be unequivocally included into the new taxon or to any other pachypleurosaur: PIMUZ A/III 721, an incomplete skull (Additional file 1: Fig. S18), PIMUZ A/III 254, a partly articulated, incomplete trunk (Additional file 1: Fig. S19), “holotype” of “*Pachypleurosaurus staubi*” (Kuhn-Schnyder, 1959); PIMUZ A/III 711, an incomplete trunk (Additional file 1: Fig. S20); PIMUZ A/III 499, “*Pachypleurosaurus* sp.” (Kuhn-Schnyder, 1952) an incomplete trunk (Additional file 1: Fig. S21), and PIMUZ A/III 720, an incomplete trunk (Additional file 1: Fig. S22). See Table 1 and Additional file 1: Figs. S1–S27, as well as the description below for further information of the state of preservation and completeness of the specimens.

## Description

### General description of specimens

The holotype (PIMUZ A/III 1274) is the largest specimen among the more complete specimens excavated in the Prosanto Formation so far (Table 2). It is exposed in dorsal view, except for a part of the tail that is preserved in lateral view. The holotype is mostly complete, only lacking the posterior half of the tail, lost before embedding. Both lower forearms are very incompletely visible in dorsal view (Additional file 1: Fig. S3) but can be roughly visualized in X-ray images (Additional file 1: Fig. S3A). The zeugopodial and autopodial elements are not preserved in anatomical correct position, instead pointing in anteromedial direction. Taphonomically this is so far a unique orientation of lower forearms among hundreds of prepared pachypleurosaur specimens (Beardmore & Furrer, 2016; Heijne et al., 2019; Sander, 1989), but a specific reason for this peculiar position of the forearms in PIMUZ A/III 1274 cannot be elucidated herein.

The right half of the skull and trunk of PIMUZ A/III 668 is missing by erosion, as well as the right hind limb and the posterior part of the tail (Additional file 1: Fig. S6). PIMUZ A/III 1240 lacks the anterior part of the skull, which was lost during excavation, and the posterior part of its tail (Fig. 5A; Additional file 1: Fig. S9). This specimen is not well preserved, but might contain organic remains. PIMUZ A/III 668 and PIMUZ A/III 1240 are both exposed in dorsal view. PIMUZ A/III 1197 is a complete individual, including the tip of the tail. However, the specimen is preserved in a rather atypical position and is further on partially disarticulated. The skull and lower jaws are exposed in ventral view (Fig. 3A), the neck in lateral view, the trunk in dorsal view, and the tail in lateral view (Additional file 1: Figs. S7, S8). The slightly disarticulated pectoral girdle and anterior limbs are preserved as a single rotated unit revealing its anatomy in ventral view (Fig. 5F; Additional file 1: Figs. S7, S8). PIMUZ A/III 4566 is preserved in ventral view. The anterior neck region is disarticulated as is the tail of which only the anterior-most part is preserved (Additional file 1: Fig. S10). Both manus are incomplete as is the right lower hind limb. The skull and girdle elements are exposed in ventral view and allow for detailed morphological study (Figs. 3A, 5D, G; Additional file 1: Fig. S10). PIMUZ A/III 1275 is, except for the phalanges of the manus and the posterior half of the tail, complete (Additional file 1: Figs. S11–S13). It is exposed in ventral view, displaying details of skull and girdle morphology (Figs. 3B; 5H). It is the smallest specimen among the referred skeletons assigned to the new taxon, likely representing a not fully grown individual (Table 2). Due to preservation, specimens in dorsal view do not expose most of the girdle elements. PIMUZ A/III 1490 is a disarticulated specimen including a well-preserved skull prepared in ventral view (Fig. 3A, B), an isolated ramus of the lower jaw, long bone elements and scattered ribs, vertebrae and gastralia (Additional file 1: Figs. S14, S15).

PIMUZ A/III 710 (Additional file 1: Fig. S17) is an incomplete skull in dorsal view with the tip of the snout missing (Bürgin et al., 1991). The skull is broadest at the posterior orbits. The size of the upper temporal openings is relatively large compared to *Anarosaurus heterodontus* or *Neusticosaurus* spp., the parietal is small and longer than wide and the posterior skull margin is deeply emarginated.

## Skull

### Dorsal view

The skulls are all dorsoventrally compressed and flattened. Partial disarticulation or shift of posterior elements (i.e., PIMUZ A/III 1240, PIMUZ A/III 1275) in anterior direction happened frequently and obscured sutures (e.g., Figs. 3B, C; 4A). The description of the skull configuration in dorsal view is mainly based on the holotype (PIMUZ A/III 1274; Fig. 2B, C), but specimens PIMUZ A/III 1240, PIMUZ A/III 668, PIMUZ A/III 710, and PIMUZ A/III 1490 additionally provide insights into dorsal skull morphology (Fig. 3A, B; Additional file 1: Figs. S14, S15). The combined information of all skulls resulted in the schematic sketches shown in Fig. 4A.

The skull shows the typical pachypleurosaur configuration with large oval orbits, medium-sized elongated oval upper temporal openings and medium-sized round–oval external naris. The skull is broadest at the posterior region of the orbits. Behind the orbits, the skull table is distinctly constricted. The upper temporal openings are relatively large when compared to other European pachypleurosaurs. The postorbital region is nearly as long as the preorbital region. The posterior skull margin is deeply emarginated, resulting in a difference when comparing condylobasal and actual length, i.e. posterior extension of the squamosal and quadrate/lower jaw length. The premaxillae have a peculiar shape with a very much restricted anterior part when compared to the condition in other European pachypleurosaurs. It only forms the anterior half of the snout and does not reach back to enter the external naris. As a result, the suture between premaxilla and maxilla is located distinctly in front of the anterolateral margin of the external naris. Each premaxilla has a very thin and long posterior processes that is framed laterally by the nasal in dorsal view. The posterior processes of the unfused premaxillae reach beyond the external naris, ending halfway between the posterior external naris and anterior orbital rim, and thus largely separating the anterior half of the elongated nasals. The anterior processes of the nasals exclude the posterior processes of the premaxillae from contact with the external naris (Figs. 2B, C, 4A). The medial and mediodorsal to posteromedial margin of the external naris is thus limited by the nasal, the remaining margin is formed by the maxilla. The maxilla reaches posteriorly as a thin tapering element beyond the mid-orbit, and maybe as far as to the posterior margin of the orbit. Laterally to the skull midline, the maxilla forms most of the space between the anterior orbit and posterior external naris. The jugal is dislocated in the holotype due to taphonomic alteration, but shape and position is well visible in other specimens (PIMUZ A/III 668, PIMUZ A/III 1197, PIMUZ A/III 1240). It is slender and lunate-shaped and lays dorsally to the maxilla forming most of the ventrolateral margin of the orbit (Additional file 1: Figs. S6B, S7B, S9B). The prefrontal is a massive bulging, boomerang-shaped element forming the anterior margin of the orbit. Prefrontal and nasal have an anterolateral contact (Figs. 2B, C, 4A). There is no evidence for a separate lacrimal. The posterior processes of the nasals extend parallel to the lateral margins of the anterior processes of the frontals (Figs. 2B, C, 4A). Medially, the anterolateral processes of the paired frontals contact the posterior processes of the premaxillae halfway between external nares and anterior margin of prefrontals. The unfused frontals limit most of the medial margin of the dorsal orbit. Their posterior lateral processes extend back to the area of the anterior parietal foramen and upper temporal openings, encompassing the anteriorly tapering processes of the parietals. Posterolaterally, the frontals contact the postfrontals and are separated from the upper temporal opening by the distinctly triangular posterior process of the postfrontal (Figs. 2B, C, 4A). This mediodorsal process extends further posterior than the posterior process of the frontal. The postorbital is a massive triradiate element. The ventrolateral process forms the posterior margin of the orbit and the long and tapering posterior process forms the dorsomedial margin of the upper temporal opening. The postorbital reaches here far posterior, beyond the upper temporal opening, approaching the level of the deeply emarginated posterior skull margin. The anteromedial process is broad and rounded, articulating on the postfrontal anterolaterally. The postorbital participates widely in the formation of the upper temporal fenestra anterolaterally. The unfused parietals encompass a relatively large parietal foramen in between their centre and show tapering anterior processes and broad posterior ones. The parietals are elongated with a narrow posterior shelf and distinct, anterolaterally angled processes accommodating the posterior process of the postfrontal in a triangular facet. The parietal is relatively small (Figs. 2B, C, 4A) compared to its dimension in other pachypleurosaur taxa, and it is longer than wide. Posterolateral processes of the parietals form ventrally the anterior margin of the supraoccipitals. The squamosal is a massive element limiting anteriorly the posterior half of the upper temporal opening and extending as far posterior as to the posterolateral margin of the parietal giving the skull roof its excavated appearance. The quadrate is rectangular in shape and located posteroventral to the squamosal, forming the most posteroventral part of the skull. A quadratojugal was identified in PIMUZ A/III 1490; but this bone is mostly obscured due to preservation and/or disarticulation of this region in other specimens. The rectangular flat and keeled supraoccipital is relatively largely exposed in dorsal view because of the strong excavation of the posterior skull table. The basioccipital (exposed in ventral view in PIMUZ A/III 1490 and to some degree in dorsal view in PIMUZ A/III 1240) is a large flat element with a dorsal central ridge (reaching into the foramen magnum), a slightly concave condyle and with prominent ventral tubera (Figs. 2B, C; 4A). Additional elements of the braincase include the exoccipitals but their shape is difficult to discern (it appears that they are fused with the opisthotics thus carrying the paroccipital process).

### Palatal view

The palatal morphology is visible in PIMUZ A/III 4566 (Fig. 3A; Additional file 1: Fig. S10E), PIMUZ A/III 1490 (Fig. 3D, E), and PIMUZ A/III 1275 (Fig. 3B; Additional file 1: Fig. S11B), although the latter is crushed and posterior elements have shifted in anterior direction, obscuring details. The combined information of all skulls resulted in the schematic sketches shown in Fig. 4B. The premaxillae form most of the anterior part of the palate, anterior to the internal naris. They are excluded from the internal nares by a posterolateral contact of the maxilla and vomer. The vomers are obscured anteriorly, but seem to end anterior to the internal naris leaving the tip of snout to be formed by the premaxillae. The vomers are separated anteriorly, but partially fused to each other close to their posterior end. The posterior processes of the vomers encompass the fused anterior processes of the pterygoids on the level of the posterior border of the internal nares. Due to damage and coverage by the dentaries in this area, it is difficult to discern the boundaries of the internal naris, but it appears that the maxilla forms the lateral margin, and the palatine the posterior and posteromedial margin of the internal naris. The pterygoid is the largest and best-preserved element in the palate. It extends from the posterior skull margin (occipital region) up to the posterior part of the external naris. The pterygoid has a triangular anterior shelf that tapers anteriorly and is separated by the posterior processes of the vomers. In the mid-part region, it is broadest, distinctly pointing here laterally. In its posterior half it is deeply waisted, giving the pterygoid a slender appearance in this area, and having lateral extending posterior-most processes, articulating with a massive quadrate (Figs. 3D, E, 4B). The posterior pterygoid carries each a lateral keel that forms the lateral margin. In the posterolateral region, the pterygoid shows deep grooves on the quadrate ramus and as evidenced by PIMUZ A/III 1490, foramina can open into the pterygoid at the anterior border of the grooves. Other specimens that are exposed in ventral view do not allow the identification of such foramina due to preservational reasons. The palatine is an elongated element forming the lateral anterior part of the palate. There is no evidence of an ectopterygoid. As the pterygoids are meeting in the midline, an interpterygoid vacuity is absent, as is any pterygoid dentition (denticles).

## Lower jaw

The lower jaw is best exposed in PIMUZ A/III 1197 where it is visible in lateral view (Fig. 3D; Additional file 1: Fig. S7A). Dentary, splenial, angular and articular show the typical pachypleurosaur condition/configuration (Rieppel, 1989; Sander, 1989). The retroarticular process seems to be more massive when compared to *Neusticosaurus* spp. and *Serpianosaurus mirigiolensis*. Additional morphological details could not be discerned.

## Dentition and tooth replacement

The teeth are conical and slender, with longitudinal striations (Fig. 3D; Additional file 1: Fig. S15) and sitting in sockets, indicating a thecodont tooth anchoring in the jawbones. The second to fourth maxillary teeth are enlarged (Fig. 3C, D). The most posterior dentary teeth become very small (Fig. 3D). Thus, there is a variation in tooth size in the upper and lower jaw but not as distinct as in for example nothosaurs and *Anarosaurus heterodontus*. Where countable, the premaxilla bears six teeth (PIMUZ A/III 1275, PIMUZ A/III 1197; PIMUZ A/III 1274, PIMUZ A/III 668). In PIMUZ A/III 1197 (Figs. 2B, 3C, 4B), nine functional maxillary teeth are visible plus six large alveoli are recognizable where (functional) teeth likely were lost due to preservation or tooth replacement. In the other specimens, the maxillary tooth rows are not well exposed, but the number of maxillary teeth seems always below ten functional teeth. The dentary of PIMUZ A/III 1197 carries 24 teeth (Fig. 3C).

PIMUZ A/III 1490 represents one of the best-preserved skulls in ventral view that comes from the Prosanto Formation. This specimen allows unique insights into dentition and tooth replacement of pachypleurosaurs that remained so far unknown (Rieppel, 2001; but see Liao et al., 2021 on *Keichousaurus hui*). The premaxillae are lost along their sutures to the maxilla, anterior to the internal naris. The maxillary tooth row reaches back to mid-orbit, whereas the maxilla extends back to the level of the posterior orbital rim. It shows eight teeth still attached to the maxilla (Fig. 3D). One of these teeth is displaced (Fig. 3D). The base of each visible tooth is smooth and does not show any striation. The upper 2/3 of each visible tooth is striated, indicative of the tooth crown. The transition to the striated part of the tooth crown is slightly constricted (but not as strong as in *Odoiporosaurus*). Proximally, the striation of the tooth is wide and then it tapers towards the tip, following the typical conical form of the tooth. The left maxilla carries nine teeth of which the first and second visible tooth are enlarged and the fifth visible tooth likely represents a replacement tooth. The second visible tooth is the largest tooth in the entire row. The last tooth is the smallest. The second largest tooth has a smaller tooth positioned directly next to its posterior margin. It is not clear if this represents an already relatively large replacement tooth in position to replace the functional tooth or if this is another functional tooth just sitting very close. Since the teeth seem to share an alveolus, it is likely that these are functional and replacement tooth. In the right maxilla, four large, empty alveoli are recognizable (Fig. 3D). The second visible alveolus is the largest one, which fits to the largest tooth on the left side. A fifth alveolus still carries a functional tooth, but this area is already damaged. The remaining part of the maxilla is fractured or lost, respectively. Between those empty alveoli four bases of functional teeth are visible, with the tooth crowns being preserved on the counter slab (Additional file 1: Fig. S15). The pulp cavities are visible (Fig. 3D). These functional teeth are separated from the alveoli by a thin septum. Anteriorly, lingually to the empty alveoli and functional teeth, two small alveoli or crypts (sensu Shang, 2007) are each carrying a replacement tooth (Fig. 3D).

## Postcranial skeleton

None of the postcranial elements, such as the vertebrae, ribs or gastralia or any other element of the new taxon show pachyostosis.

## Axial skeleton

### *Vertebrae*

An atlas centrum is exposed in PIMUZ A/III 4566 and PIMUZ A/III 1275 (Fig. 3A, B). It is of round–oval shape and seems to be much smaller than the centrum of the axis.

From those specimens that have a complete and articulated neck preserved, the holotype (PIMUZ A/III 1274) and PIMUZ A/III 1275 have 18 cervicals (Table 2; Fig. 2A; Additional file 1: Figs. S1, S2, S4, S11, S12), whereas PIMUZ A/III 4566 and PIMUZ A/III 1240 show 17 and 15 cervicals, respectively (Table 2; Additional file 1: Figs. S9, S10). The centra of the cervicals are not constricted and each carry two lateral ridges ventrally (Additional file 1: Figs. S10–S12). The length and width of the centra increases continuously in posterior direction (Additional file 1: Figs. S10–S12). In the anterior neck region, the centra are longer than wide; in the mid-posterior region they are nearly as wide as long, and the last two centra are wider than long. The cervical centra carry both facets for the double-headed ribs (i.e., di- and parapophysis). The neural arches are roughly rectangular with the lateral margins between pre- and postzygapophyses slightly concave. The neural spine is distinct, but remains low throughout the neck (Fig. 2A; Additional file 1: Figs. S1, S7C, S9A).

The number of dorsal vertebrae is 24 in the holotype (Fig. 2A). Other specimens preserving a complete trunk region (i.e., shoulder girdle to pelvic girdle, including sacral region) show 23–25 dorsal vertebrae (Table 2; Additional file 1: Figs. S1, S2, S9–S12). The dorsal centra are longer than wide and not constricted. The neural arches of the dorsals have a slightly protruding transversal process, making the neural arch here somewhat wider than anteriorly and posteriorly (Fig. 2A, D, F; Additional file 1: Figs. S1, S2, S7). The area between the transversal process and postzygapophyses is constricted, resulting in the typical butterfly-shape of the eosauropterygian neural arch. The neural spine remains low throughout the trunk region.

Sacral vertebrae are usually identified by their joined di- and parapophysis, resulting in a large, roundish rib facet. However, the last dorsal and the first caudal vertebra tend to show “sacralization” in *Simosaurus* (Rieppel, 1994) and other Eosauropterygia (Rieppel, 2000; pers. obs. NK), meaning that these vertebrae also have a large roundish joined rib facet. Thus, sacral vertebrae are best identified with the help of their associated sacral ribs, which are more characteristic (see below). Based on this, the number of sacral vertebrae in the Prosanto pachypleurosaurs is three (Fig. 2F; Additional file 1: Figs. S1E, S6H, I, S9F).

The tail is complete in PIMUZ A/III 1197, exposing in right lateral view 61 caudal vertebrae (Additional file 1: Figs. S7, S8), just a few vertebrae shorter than the long-tailed Chinese pachypleurosaur *Honghesaurus longicaudalis*, which was reported with 69 caudals (Xu et al., 2022). In PIMUZ A/III 1274 (17 caudals), PIMUZ A/III 668 (14 caudals), and PIMUZ A/III 1275 (18 caudals) only the anterior half of the tail is preserved. In PIMUZ A/III 1240 about 2/3 (~ 35 caudals) of the tail is preserved but the posterior part is disarticulated from the rest (Additional file 1: Fig. S9A). Shortly behind the sacrum the tail is disarticulated but about 25 caudal centra can still be counted in PIMUZ A/III 4566. Where visible, the Prosanto specimens show ‘sacralization’ in the shape of the 1st caudal centrum (PIMUZ A/III 1274, PIMUZ A/III 1240, PIMUZ A/III 668; Fig. 2F; Additional file 1: Figs. S1E, S6H, I, S9F) even if the transverse processes are distinct from the sacral ribs. The joined rib facet is formed by the centrum and neural arch, but with each caudal vertebra it is located further ventrally until the centrum carries the rib facet solely, which is between the 6th (holotype PIMUZ A/III 1274), 7th (PIMUZ A/III 1197), 7th or 8th (PIMUZ A/III 668) or the 8th (PIMUZ A/III 1275) caudal.

The centra carry rib facets until the 11th (PIMUZ A/III 1197), 13th (PIMUZ A/III 1274, PIMUZ A/III 668), or 10th caudal. However, the transversal process visible at the respective caudal is always only rudimentary, likely not having been connected to a rib. The caudal centra are somehow awkward and are laterally constricted. All centra are longer than wide and increase in length but decrease in height. The last ones (PIMUZ A/III 1197) are twice as long as wide or high (Additional file 1: Fig. S7). All specimens show high neural spines in the anterior part of the tail (Additional file 1: Figs. S1F, 6A, 7F). In PIMUZ A/III 1197 the neural spine is most massive and highest in caudal 4 to 6, afterwards it decreases and the neural spine is absent after the 10th caudal. The neural arch decreases continuously in size until it disappeared completely about the 43rd caudal. In the holotype (PIMUZ A/III 1274), the neural spines increase in height and massiveness from the 1st until the 3rd caudal; they are highest and most massive in caudal 4–8; from the 9th caudal towards posterior the size of the neural spines decreases and is lost in the 12th caudal. In PIMUZ A/III 668, distinct, nearly equally high neural spines are visible in the 5th to 11th caudal, whereas the ones of the 12th and 13th are damaged but still recognizable. Pre- and postzygapophyses lay nearly horizontally throughout the entire vertebral column, allowing for lateral movement of the column.

#### Ribs

Cervical ribs are only visible in specimens preserved in ventral (PIMUZ A/III 1275, PIMUZ A/III 4566) or lateral view (PIMUZ A/III 11997) (Fig. 3B; Additional file 1: Figs. S7C, S10A, S11A, C). The cervical ribs are firmly attached to the cervicals and not well to distinguish from the centra, which hampers their description. In the mid-posterior neck region of PIMUZ A/III 1275, some cervical ribs had become disarticulated from the cervicals. The cervical ribs are double-headed and have the typical free anterior and posterior process as described for other pachypleurosaurs and Eosauropterygia (summarized in Rieppel, 2000). In this region of the neck, both processes are of an equal length. Further on, PIMUZ A/III 1275 shows the change from typical cervical ribs towards a dorsal rib morphology. In the last three posterior cervical ribs, the posterior process is getting longer and shows a pointed end, whereas the anterior process is lost/reduced, approaching dorsal rib shape (Additional file 1: Fig. S11C, 13C).

The dorsal ribs have a broad, rectangular proximal head that articulates with the respective vertebra. Behind the articulation facet the rib is constricted. The proximal part becomes thick, round–oval, carrying a protruding dorsal hump. The proximal part is rather straight, pointing laterally. The rib then soon tapers and curves distally. It is slender in its mid-region before it broadens again to a flat distal, striated end (Fig. 2 A, D, F; Additional file 1: Figs. S1–S4, S6A, G, S7B, S9D). The first dorsal rib is rather short, the following ribs continuously become longer. The ribs are longest in the posterior trunk region. The 21st–23rd dorsal rib are getting shorter and less curved again. The 24th rib shows a distinct morphology: it is short, with a convex anterior and posterior margin, and a pointed distal end, oriented straight in lateral direction (Fig. 2F; Additional file 1: Figs. S1E, S6H, I, S9F).

The Prosanto specimens have three short sacral ribs (Fig. 2F; Additional file 1: Figs. S1E, S6H, I, S9F), which are very characteristic due to a distinct distal broadening, making them proximally and distally equally broad but leaving the shaft region somewhat (1st, 2nd) and distinctly (3rd) constricted. The rib facet is large and round–oval; the proximal part is not constricted. In the holotype, the sacral ribs are roughly rectangular elements that are medially and distally as long as proximally, with the 3rd sacral rib even being longer than the proximal and medial part (Fig. 2F). However, all three specimens that have the sacral ribs exposed show a certain variability (Additional file 1: Figs. S1E, S6H, I, S9F). In PIMUZ A/III 668 and PIMUZ A/III 1240 is the 3rd sacral rib medially constricted/shorter than proximally and distally. All three sacral ribs articulate in all specimens distally with the ilium, i.e., the 1st sacral points posteriorly the 2nd, is rather straight and the 3rd points anteriorly. In PIMUZ A/III 1274 the 3rd sacral rib is the most strongly developed one, in PIMUZ A/III 668 and PIMUZ A/III 1240 the strongest one is the second sacral rib.

The first three caudal ribs are approximately as long as the sacrals and the last dorsal rib. The proximal part is slightly constricted. Their anterior margin is convex after the constriction, whereas the posterior margin is straighter. The distal end is pointed. From the fourth caudal rib the rib length decreases significantly and around the 10th, the rib is only rudimentarily developed.

Chevrons are best exposed in lateral view. In PIMUZ A/III 1197 the first visible chevron lies between the 6th and 7th caudal vertebrae (Additional file 1: Fig. S7B, F, G). More anterior ones might be lost due to preservation or are absent in this anterior-most part as in other pachypleurosaurs (Carroll & Gaskill, 1985; Rieppel, 1989; Sander, 1989). In PIMUZ A/III 668, chevrons appear between the 5th and 6th caudal (Additional file 1: Fig. S6A). The shape is roughly anchor-like, with a more pronounced anterior process when compared to the posterior process until the fourth chevron. In the 5th chevron anterior and posterior processes are equally short. Altogether 9 (or maybe 10) chevrons can be counted in PIMUZ A/III 1197, the last between the 15th and 16th. The tail of PIMUZ A/III 668 is incomplete; between the last visible caudals (13th and 14th) is still a well-developed chevron preserved. Based on the complete tail of PIMUZ A/III 1197, the chevrons seem to disappear much earlier than in *N. pusillus* and *N. peyeri* where the chevrons reach until the 23rd–27th caudals (Sander, 1989) or in *N. edwardsii* (28th–29th caudals; Carroll & Gaskill, 1985) and in *Serpianosaurus mirigiolensis* (28th–31st; Rieppel, 1989).

#### Gastralia

None of the gastralia is well exposed and articulated. However, due to the high number of elements associated in the gastral apparatus with PIMUZ A/III 1275 and PIMUZ A/III 4566 (Additional file 1: Figs. S10–S12), each gastralium likely consists of 5 elements: a short and more massive median element and two slender rods in line towards each side. Towards distal, disarticulated gastral elements have shifted medially, lying now parallel to the more medial ones, resulting in a similar pattern as described and figured for *Serpianosaurus* that also has gastralia composed of 5 elements (Rieppel, 1989). The median part of the gastral element is well visible underlying the ventral side of the vertebral centra (Additional file 1: Figs. S10A, C, S11A, H), displaying a short pointed anterior process. In the anterior trunk region, one gastralium per dorsal centrum can be located, whereas in the mid and posterior trunk region, where centra become longer, two gastralia per centrum can be found (Additional file 1: Figs. S10A, C, S11A, H).

#### Appendicular skeleton

In PIMUZ A/III 1275 (Additional file 1: Fig. S10A, B) and PIMUZ A/III 4566 (Additional file 1: Fig. S11A), the interclavicle is damaged. The interclavicle is thus only well exposed in PIMUZ A/III 1197 (Fig. 5F). It is still articulated with the right clavicle. It is a small rhomboidal element without a visible anterior process. The clavicle tapers at both its ends: medially and posterolaterally and is without anterolaterally expanded corners, giving the shoulder girdle a roundish appearance (Figs. 2D, 5F). The medial part is thick in dorsoventral direction; the lateral part is dorsolaterally flat, which is the result of a twist between both parts. The posterolateral part extends medial to the dorsal part of the scapula (Fig. 2D; Additional file 1: Figs. S1C, S6A, S10B, S11A). The scapula has the typical massive anteroventral blade with an elongated tapering dorsal shaft (Fig. 2D; Additional file 1: Figs. S1A, C, S2, S3, S6A, S7B, S9A, S11D). The length of the dorsal shaft varies: it is long in PIMUZ A/III 1274 (Fig. 2D) and PIMUZ A/III 668 (Additional file 1: Fig. S6A) but rather short in PIMUZ A/III 1275 (Fig. 5E) and PIMUZ A/III 1197 (Additional file 1: Fig. S7B). In PIMUZ A/III 1274 a large half round coracoid foramen is visible at the medial margin of the anteroventral blade of the scapula (Fig. 2D) and is also present in PIMUZ A/III 668 (Additional file 1: Fig. S6A) but it is not discernible in other scapulae (Fig. 5D–F; Additional file 1: Figs. S7H, S10A, B, S11A, D, S9–S11). The coracoid has a distinctly constricted shaft, i.e., middle part, which is the result of its deeply concave anteromedial margin (Fig. 5D, F). The shape of the coracoids is variable. It is more massive and stouter in PIMUZ A/III 1275 (Additional file 1: Fig. S11A) and it is less constricted in PIMUZ A/III 4566 (Fig. 5D) and PIMUZ A/III 1197 (Fig. 5F). The latter two have a much shorter medial suture then in PIMUZ A/III 1275.

In PIMUZ A/III 1240, the ilium is preserved in its correct anatomical position with the sacral ribs contacting the broad ventral part (Additional file 1: Fig. S9F), whereas it is dislocated in all other specimens. In PIMUZ A/III 668 the ilium is exposed in lateral view, displaying a short but distinctly set-off iliac blade that is posteriorly pointed (Additional file 1: Fig. S6H, I). In PIMUZ A/III 1197 the iliac blade is less distinct, it is more a dorsal, posteriorly curved process, which is even more the case in other specimens (e.g., Additional file 1: Fig. S7J). In PIMUZ A/III 1240 only a short dorsal process of the ilium can be recognized. It is relatively longer in PIMUZ A/III 1274 and PIMUZ A/III 668, and longest in PIMUZ A/III 1275 (Fig. 5H). The pubis has a large, protruding articulation facet to the ilium/acetabulum (Fig. 2A, G). The anterior margin of the pubis is slightly concave, whereas the posterior margin is deeply concave (Fig. 5H). The lateral margin is simple convex in PIMUZ A/III 1275, but divided into two straight parts in the others. The medial margin is straight to slightly convex and the anteromedial edge is pointed in PIMUZ A/III 1275 (but not forming a prepubis). A slit-like obturator foramen is visible in PIMUZ A/III 1197, PIMUZ A/III 4566, and PIMUZ A/III 1275 (Fig. 5G, H). The latter, however, represents a not fully grown individual. For the other individuals, an obturator foramen is not discernible due to preservation. The ischium has the typical shaft-like lateral process and a fan-shaped medial part. The medial margin is asymmetrically formed (Fig. 5G, H). The lateral shaft-like part of the ischium is shorter and stouter in PIMUZ A/III 1275 and PIMUZ A/III 1240 when compared to the other specimens.

As typical for pachypleurosaurs, humerus morphology is highly variable (Additional file 1: Figs. S1A, G, S3B, C, S6A, C, S7B, I, S9A, C, S10A, F, G, S11A, F, S16A, B, S19, S20, S24–S26). It has an angled proximal head, a constricted shaft, and a flat and broad distal end (Figs. 2A, E; 5A–C). All humeri show an entepicondylar foramen. The distal end is always broader than the proximal head. In the two largest humeri, PIMUZ A/III 1274 (Fig. 2E) and PIMUZ A/III 1197 (Additional file 1: Fig. S7I), the proximal head is set off in an angle of roughly 80°–85°, the postaxial/lateral margin is rather straight, the preaxial/medial one curved, and the deltopectoral crest is distinct. The smaller specimen PIMUZ A/III 4566 (Additional file 1: Fig. S10A, F, G) has a similar rectangular proximal head, although it is not set off, and a similar shaft morphology. In PIMUZ A/III 668 (Fig. 5C) and PIMUZ A/III 1240 (Fig. 5A), the shaft is constricted from post- and preaxial side, the proximal head appears more massive but it is not as clearly set-off as in the other ones. The humerus of PIMUZ A/III 668 (Fig. 5C) is very stout and massive. The humerus PIMUZ A/III 1275 (Fig. 5B) shows a rather simple/undifferentiated morphology, supporting its juvenile ontogenetic stage (Sander, 1989).

The ulna is shorter than the radius (Table 2). The radius has a massive proximal head with a lateral/postaxial ridge, a slender, constricted shaft and gracile distal end (Fig. 5A–C; Additional file 1: Figs. S6D, S7B, S9A, S10A, S11A, S16C). The ulna is broad and symmetrical, with the proximal and distal end equally wide and a slightly constricted shaft (Fig. 5A–C; Additional file 1: Figs. S6D, S7B, S9A, S10A, S11A, S16C). The ulna is more constricted in PIMUZ A/III 1240 than in the other specimens. Two carpalia are present in PIMUZ A/III 1197, PIMUZ A/III 668, PIMUZ A/III 1240, and PIMUZ A/III 1275 (Fig. 5A, B; Additional file 1: Figs. S6A, S7B, S9D, S11A). In the other specimen, the number cannot be determined due to disarticulation and preservation (PIMUZ A/III 1274, PIMUZ A/III 4566). Based on observation in all specimens, the phalangeal formula of the manus is 2-3-4-4/5-3, with the 2nd digit being always the longest.

The femur is a relatively straight bone with a pronounced proximal head, which is, depending on view/preservation nearly triangular, a straight, constricted slender shaft, and a small, not very distinct distal end (Fig. 2A, G; Additional file 1: Figs. S1A, H, I, S6A, S7B, J, S9A, E, S10A, H, S11A, G). The distal condyli are not well developed. The femur is longer than the humerus in PIMUZ A/III 4566 and PIMUZ A/III 1275 but shorter than the humerus in the other specimens (Table 2). The tibia is roughly half the length of the femur, the fibula is always slightly longer than the tibia (Table 2; except for PIMUZ A/III 1197). The tibia is rectangular, i.e., it is nearly proximally and, in the shaft, as wide as distally (Fig. 2A, G). All specimens show two tarsalia. Astragalus and calcaneus are both well developed, with the astragalus having an anterior concavity and the calcaneus being round–oval (Fig. 2A; Additional file 1: Figs. S1H, I, S7B, S9A, E, S10A, H, S11A). In all specimens, the pes is larger and more massive when compared to the manus. The 1st toe is short (less than half the length of the longest finger) and massive and the fourth toe is always the longest (Additional file 1: Figs. S1A, H, I, S6A, S9A, E, S11A). Where completely preserved as for example in the holotype PIMUZ A/III 1274, the phalangeal formula is 2-3-4-5-3 (Fig. 2A, G).

## Morphological comparison

### Comparison of the newly described taxon with other pachypleurosaur specimens from the Prosanto Formation

For a complete list of specimens see Table 1, and for additional figures of Prosanto specimens see Additional Figures. PIMUZ A/III 721 (Additional file 1: Fig. S18) represents a poorly preserved but complete skull in dorsal view. The skull is broadest at the posterior orbit, but the posterior skull margin is not distinctly emarginated. The posterior skull region is too damaged to address the shape and extension of the parietal.

PIMUZ A/III 254 (holotype and only known specimen of “*Pachypleurosaurus staubi*” Kuhn-Schnyder, 1959) comes from the same geographic region and strata (Table 1), as the newly described specimens. Its morphology is described in detail in Kuhn-Schnyder (1959). The specimen consists of an incomplete trunk region of a not yet fully grown individual (trunk length is about 12 cm; Additional file 1: Fig. S19). The right forearm and hind limb are persevered but manus and pes are disarticulated and incomplete. The left humerus is highly damaged. None of the bones shows pachyostosis. The humerus shows a constricted shaft and an only slightly (if at all) set off proximal head. The ulna is symmetrical and broad as well as slightly shorter than the radius. The femur is straight and slender. The straight, rectangular tibia and the curved fibula resemble the typical pachypleurosaur morphology. The ilium has a posterior pointing dorsal iliac process. The three sacral ribs are distally as broad as proximally. None of the described above morphological structures allow an unambiguous genus and species assignment of PIMUZ A/III 254.

PIMUZ A/III 711 (Additional file 1: Fig. S20) consists of a left trunk region in dorsal view with the left scapula and humerus preserved (Bürgin et al., 1991). As indicated by the small size of the trunk (~ 7–8 cm) as well as by the rather simple rectangular humerus morphology, the specimen represents a not fully grown, likely juvenile individual. It does not show pachyostosis. Gastralia are visible and seem to be composed of five elements.

PIMUZ A/III 499 (Additional file 1: Fig. S21) also displays only an incomplete trunk (Kuhn-Schnyder, 1952). The ribs and vertebrae are not pachyostotic. Three sacral ribs are preserved that are distally as broad or broader than proximally. The ilium has a dorsal tapering process.

Another incomplete trunk region (PIMUZ A/III 720; Additional file 1: Fig. S22) has only dorsal vertebrae and ribs preserved. Gastralia are well exposed between the ribs and seem to be composed of five elements. This specimen represents the largest individual from the Prosanto Formation, with a trunk width about 40% larger than that of the holotype PIMUZ A/III 1274.

### Comparison of the new taxon with other European pachypleurosaurs

Three unique characters in skull morphology, as listed in the diagnosis above, differentiate *Prosantosaurus scheffoldi* gen. et spec. nov. from other pachypleurosaurs, whereas many morphological features and morphometrics of the postcranium are shared. The premaxilla is excluded from contact with the external and internal nares in *Prosantosaurus* *scheffoldi* gen. et spec. nov., whereas in all other European pachypleurosaurs for which this can be documented, the premaxillae always enter the external and internal nares. According to Rieppel and Lin (1995:26), the premaxilla is in contact with the external naris but not with the internal naris in *Nothosaurus* and *Cymatosaurus*. The small size of the longer than wide parietals with distinct, anterolaterally angled processes is unique in the new taxon. *Anarosaurus* spp. and *Dactylosaurus gracilis* also have longer than wide parietals but their shapes differ. In *Anarosaurus* spp. and *Dactylosaurus gracilis* the anterior processes of the parietals are broad with an interdigitating suture to the posterior processes of the frontals, whereas the anterior processes of the parietals in the new taxon taper and are encompassed by the posterior processes of the frontals. *Serpianosaurus mirigiolensis* and *Odoiporosaurus teruzzii* have parietals that are only slightly wider than long, whereas *N. pusillus*, *N. peyeri* and *N. edwardsii* have distinctly wider than long parietals. The new taxon has a deeply emarginated posterior skull margin, which it shares with *Anarosaurus* spp. and *Dactylosaurus gracilis*. *Neusticosaurus edwardsii* differs from *Prosantosaurus scheffoldi* gen. et spec. nov. by a greater length of the antorbital skull region relative to the postorbital skull region and broader frontals, i.e., a broader orbital bridge (Carroll & Gaskill, 1985). *Prosantosaurus scheffoldi* gen. et spec. nov. differs from *Anarosaurus* spp. and *Dactylosaurus gracilis* in having a proportionally shorter snout. In *Prosantosaurus* *scheffoldi* gen. et spec. nov., the maxilla reaches as far back as almost to or up to the posterior margin of the orbit, a feature that it shares with *Anarosaurus heterondontus* and *Dactylosaurus gracilis*. In *Odoiporosaurus teruzzii*, the maxilla extends not as far posteriorly as in the latter but it reaches here distinctly more posterior when compared to *Neusticosaurus* spp. and *Serpianosaurus mirigiolensis.* In the new taxon, the premaxillae and frontals contact each other in a short interdigitating suture halfway between the external naris and the anterior margin of the prefrontals, a feature shared with *Odoiporosaurus teruzzii*, and *Neusticosaurus* spp. In all other European pachypleurosaurs (*Anarosaurus* spp., *Dactylosaurus gracilis*, *Serpianosaurus mirigiolensis*), the premaxillae and frontals are separated by the nasals. Further, the nasals in the new taxon articulate with the anteromedial margin of the prefrontal and have no further tapering process extending posteriorly in between the frontal and prefrontal or into the frontal. The lack of a posterior tapering and with the frontal interdigitating process of the nasal is shared with *Anarosaurus pumilio* and *Dactylosaurus gracilis*. *Prosantosaurus scheffoldi* gen. et spec. nov. has a distinctly mediodorsally pointing triangular posterior process of the postfrontal that reaches further posterior than the posterior process of the frontal. *Odoiporosaurus teruzzii*, *A. pumilio*, and *Dactylosaurus gracilis* share a similar form and extension of the mediodorsally posterior process of the postfrontal, but this is different in *Neusticosaurus* spp. and *Serpianosaurus mirigiolensis*. The postorbital of the new taxon has a broad and rounded anteromedial process articulating on the postfrontal anterolaterally and a long and tapering posteromedial process that participates widely in the formation of the anterolateral upper temporal fenestra. This differentiates *Prosantosaurus scheffoldi* gen. et spec. nov. from *Neusticosaurus* spp. but is similar to the condition seen in *Serpianosaurus mirigiolensis*, *Anarosaurus* spp., and *Dactylosaurus gracilis*. The squamosal extends in the new taxon as far posterior as to the posterolateral margin of the posterior parietal, giving the skull roof its excavated appearance, a character shared with *Anarosaurus* spp. and *Dactylosaurus gracilis*. The shape and posterior constriction of the pterygoid is unique in *Prosantosaurus* *scheffoldi* gen. et spec. nov. The pterygoid of *Neusticosaurus pusillus* and *Serpianosaurus mirigiolensis* are slender and constricted as well. However, the constriction is less deep and the posterior-most lateral processes are lacking (*N. pusillus*) or are not as pronounced (*Serpianosaurus mirigiolensis*) when compared to the new taxon. The skull of *Prosantosaurus scheffoldi* gen. et spec. nov., is broadest at the posterior region of the orbits as is also the condition in *Serpianosaurus mirigiolensis* and *Dactylosaurus gracilis*. This is less pronounced in *Anarosaurus* spp. The relatively large upper temporal openings, shares the new taxon with *Anarosaurus* spp. and *Dactylosaurus gracilis*. The teeth of the new taxon are less pointed and less constricted at their basis when compared to other pachypleurosaurs.

Humerus morphology in all European pachypleurosaurs is highly variable, which is related to ontogenetic and individual variation as well as to sexual dimorphism (Klein, 2010, 2012; Rieppel, 1989; Rieppel & Lin, 1995; Sander, 1989). In the new taxon, all three sacral ribs are proximally and distally equally broad/expanded, which is contrary to the usually pointed 1st and 3rd sacral ribs in *Neusticosaurus* spp., *Serpianosaurus mirigiolensis* (Carroll & Gaskill, 1985; Rieppel, 1989; Sander, 1989). In *Anarosaurus* spp., the sacral ribs are proximally broader than distally and in *Dactylosaurus gracilis*, the sacral ribs are proximally and distally of equally broad similar as in the new taxon but in *Dactylosaurus* the 1st sacral rib is the most massive element. All *Neusticosaurus* spp. have the gastralia composed of three elements (e.g., Rieppel, 2000), contrary to the condition in *S. mirigiolensis*, *O. teruzzii*, *Anarosaurus* spp., *Dactylosaurus gracilis*, and the new taxon. The Prosanto specimens, *O. teruzzii*, *S. mirigiolensis*, *Anarosaurus* spp., and *Dactylosaurus gracilis* all lack pachyostosis in the postcranium. Pachyostosis in adults however, is common in *Neusticosaurus* spp. (Rieppel & Lin, 1995). A lower count, i.e. posterior extension, of chevrons in *Prosantosaurus* *scheffoldi* gen. et spec. nov., when compared to *Neusticosaurus* spp. and *Serpianosaurus mirigiolensis*, might be related to a less developed swimming tail and could have influenced swimming abilities. Due to incompleteness, nothing can be said about the posterior extension of chevrons in the other taxa.

## Phylogenetic analyses

**Fig. 6:**
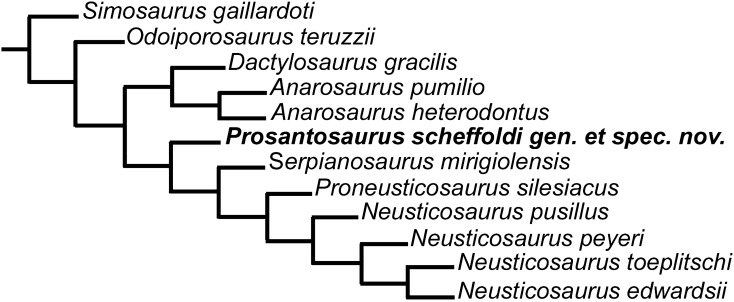
Phylogenetic relationships of European Pachypleurosauria and *Prosantosaurus scheffoldi* gen. et spec. nov. Cladogram resulting from Traditional Search (TNT Version 1.5; settings: 100,000 replications, 1000 trees held per replication, TBR active, outgroup *Simosaurus*; Memory settings: 30,000 trees, 1000 MB) with the eight specimens from the Prosanto Formation treated as a single OUT. Tree length is 178 steps. The New Technology Search revealed the same topology and tree length (Additional file 1: Fig. S31)

The matrix was analysed under maximum parsimony in TNT 1.5 using Traditional Search option with 100,000 replications, 1000 trees saved per replication and tree bisection reconnection (TBR) set as active and using the New Technology Search option with standard settings (Goloboff et al., 2008). None of the characters was treated as ordered or weighted. The matrix includes 82 characters and *Simosaurus gaillardoti* was set as outgroup. In a first analysis, the eight specimens from the Prosanto Formation had been coded individually as operational taxonomic units (OTUs) to elucidate whether any would cluster with other pachypleurosaur species or not. The analysis yielded one most parsimonious tree (MPT) with 186 steps (CI 0.640, RI 0.690). The tree topology of the MPT revealed all Prosanto Formation specimens to fall either into a basal polytomy or in a successive grade (Additional file 1: Figs. S28–S31). In a second step, the Prosanto Formation specimens were coded as a single OTU. This analysis recovered a single MPT with 178 steps (CI 0.669, RI 0.727). In this analysis, the new taxon was recovered as sister taxon to a clade comprising *Serpianosaurus*, *Proneusticosaurus*, and the *Neusticosaurus* spp. (Fig. 6).

## Discussion

### Phylogenetic implications

*Odoiporosaurus* was previously recovered as the sister taxon to a clade consisting of *Serpianosaurus mirigiolensis* and *Neusticosaurus* spp. (Renesto et al., 2014) or as sister to a clade including *Dactylosaurus* and *Anarosaurus* (Lin et al., 2021), in the latter it is unclear whether only *A. heterodontus* was used or if *A. pumilio* is included in the coding. In our analysis*,* however, *Odoiporosaurus teruzzii* did not cluster together with the other Alpine taxa from Monte San Giorgio or other taxa from the Germanic Basin but instead represents the sister to all included pachypleurosaur species (Fig. 6). The Germanic Basin taxa, *Dactylosaurus gracilis* and the two species of *Anarosaurus* group together forming the sister clade to a clade containing the taxa from the Alpine Triassic plus *Proneusticosaurus* from the Germanic Basin. The new taxon, *Prosantosaurus scheffoldi* gen. et spec. nov., was recovered in the second analysis as the sister taxon of the clade including *Serpianosaurus*, *Proneusticosaurus*, and the monophyletic *Neusticosaurus* spp., with the latter’s relationships being (*pusillus* (*peyeri* (*toeplitschi*, *edwardsii*))). Although the consistency and retention indices were almost 0.68 and 0.71, overall support of the tree is low. *Proneusticosaurus silesiacus* Volz, 1902, an incomplete taxon from the Early Anisian of Poland (Klein & Surmik, 2021) was included for the first time in a phylogenetic analysis. Including or removing it from the analyses did not have any impact on the rest of the topology. However, it is interesting that it always groups within the group of Alpine Triassic pachypleurosaurs, independent of settings or taxa included or excluded. The inclusion or exclusion of *N. toeplitschi* also did not have any impact on the topology.

As we did not include Chinese specimens in our analyses, we cannot compare results to the latest analysis of Lin et al. (2021) that recovered (*Wumengosaurus*, *Qianxisaurus*) as sister to (*Neusticosaurus*, S*erpianosaurus*), thus proposing a hypothesis in which the European pachypleurosaur taxa no longer form a monophyletic clade. Further analyses are needed to address this specific question.

### Evaluation of characters and osteological variability in pachypleurosaurs

The high number of individuals available for *Neusticosaurus* spp., *Serpianosaurus mirigiolensis*, and *Keichousaurus hui* clearly documents a very high intraspecific variability and developmental plasticity. Furthermore, ontogenetic variation and sexual dimorphism are obvious, as well as functional similarities (i.e., secondary aquatic adaptations) and differences in preservation (e.g., compaction or dorsal vs. ventral view). These are all important factors, making it extremely difficult to find reliable morphological or morphometric characters to test phylogenetic relationships of pachypleurosaurs and/or identify new taxa. In the end, unambiguous morphological characters diagnosing a pachypleurosaur taxon are extremely rare and character combinations must be used.

Thus, for example, fusion of skull elements as well as general skull and postcranial morphology is intra- and interspecifically variable in pachypleurosaurs, and is additionally related to ontogeny and preservation. The presence or absence of a coracoid foramen as well as that of an obturator foramen also depends on preservation and likely on ontogeny as well (Rieppel & Lin, 1995). Further on, also the number of cervical-, dorsal-, sacral- and caudal vertebrae is highly variable and not a reliable phylogenetic character/autapomorphy for species (e.g., Carroll & Gaskill, 1985; Rieppel, 1989; Sander, 1989; Table 2 this study). The humerus and other elements, as well as certain related morphometrics are subject to sexual dimorphism and thus variable as well (e.g., Cheng et al., 2009; Lin & Rieppel, 1998, 1998; Rieppel, 1989; Sander, 1989).

The new taxon described from the Prosanto Formation, *Prosantosaurus scheffoldi* gen. et spec. nov., lies with most of its morphological and morphometric features always within the range of one or even more taxa, notably those of *N. pusillus* (e.g., presacral count) and *Serpianosaurus* (e.g., gastralia composed of five elements; humerus morphology). However, a few unique characters have been identified (see above). As typical for pachypleurosaurs known by larger specimen numbers, the six Prosanto specimens show a certain variability in their postcranial elements and element counts (Table 2). For example, the stouter, more massive coracoid and ischium in PIMUZ A/III 1275 might be related to its relatively smaller size and thus an earlier ontogenetic stage. The simple humerus morphology of this specimen supports the hypothesis of a not yet fully grown individual. However, among the Chinese pachypleurosaur *Keichousaurus* some individuals identified as pregnant females are about 1/3 smaller than the maximum known body length and display a rather simple humerus morphology (Cheng et al., 2004). Similarly, small individuals with a simple humerus morphology were interpreted as representing one sex in *Neusticosaurus* as well (Sander, 1989).

Morphological variability in humerus morphology is thus either a result of an earlier ontogenetic stage or sexual dimorphism. However, sample size is too small for the Prosanto specimens to go into more detail here. That the femur is longer than the humerus in PIMUZ A/III 4566 and PIMUZ A/III 1275 but shorter than the humerus in the other specimens might be related to simple individual variability or also to ontogeny and/or sexual dimorphism.

Interestingly, preservation and preparation, i.e., whether the specimen was embedded and/or prepared in dorsal or ventral view, seems to have an impact on the phylogenetic relationships in our analyses. The two specimens prepared in ventral view plot closer together than the four specimens in dorsal view.

As such, the occurrence of more than one pachypleurosaur taxon in the Prosanto Formation cannot be completely excluded. Some of the specimens described above represent not fully grown individuals, which further hampers taxonomical assignment. Intraspecific- and ontogenetic variability, developmental plasticity as well as sexual dimorphism complicates detailed comparison, phylogenetic analyses, and in general finally taxonomical identification of Pachypleurosauria. This further results in a lack of unequivocal species characters (autapomorphies) in pachypleurosaurs (e.g., Carroll & Gaskill, 1985; Rieppel, 1989; Sander, 1989; Cheng et al., 2009). Thus, pachypleurosaur taxa are usually defined based on various cranial and postcranial character combinations and incomplete specimens can often only be assigned to the group or genus level. All incomplete specimens from the Prosanto Formation are treated accordingly as Pachypleurosauria indet. (Table 1).

### Comments on tooth replacement patterns in Pachypleurosauria

According to Rieppel (2001), pachypleurosaurs are usually too poorly preserved to investigate details of tooth implantation or replacement in spite of numerous available specimens. Only recently the tooth replacement was studied, among other dental aspects, in the Chinese pachypleurosaur *Keichousaurus hui* (Liao et al., 2021) in detail. The authors indicate that the replacement teeth develop in this species lingually to the pulp cavities of the functional teeth, linking it with the ‘iguanid tooth replacement type’ of reptiles.

PIMUZ A/III 1490 from the Prosanto Formation and its special preparation provides now insights in this matter also for European pachypleurosaurs. As is evident from this specimen (Fig. 3D, E; Additional file 1: Fig. S15), the replacement teeth develop in their own crypts (= the special alveoli where the replacement teeth develop, Shang, 2007) and then move anterolabially into the now empty alveoli as in *Nothosaurus* (Rieppel, 2001). The tooth in this alveolus is resorbed in the time the tooth posteriorly to it is in function. Thus, tooth replacement in the new pachypleurosaur *Prosantosaurus scheffoldi* gen. et spec. nov. happens horizontally and alternating as in *Nothosaurus* and *Cymatosaurus* and not vertically as in the crushing teeth of placodonts (Neenan et al., 2014; Rieppel, 2001). Thus, replacement pattern of European *Prosantosaurus scheffoldi* gen. et spec. nov. differs distinctly form that described for the Chinese pachypleurosaur *Keichousaurus hui* (Liao et al., 2021). Spiekman and Klein (2021) had described the development of replacement teeth directly lingual to the functional teeth in cf. *Lamprosauroides goepperti*, which might also represent a member of Sauropterygia. In spite of differences in the replacement pattern, *Keichousaurus hui* also shows an alveolarization of replacement teeth with the latter sitting in the same alveoli as the functional teeth (Liao et al., 2021). Rieppel (2001:215) wrote: “The “alveolarization” of replacement teeth necessitates remodelling of the functional alveoli during tooth replacement, which is unique among reptiles (Edmund, 1960) and supports the monophyly of Sauropterygia, including both placodonts and eosauropterygians (Rieppel, 2000). Thus, this monophyly is now supported by the tooth replacement patterns of pachypleurosaurs as well.

### Stratigraphic and palaeogeographic distribution

The stratigraphically oldest European pachypleurosaurs are documented from localities of Early Anisian age. During this time (Fig. 1A; Additional file 1: Fig. S27), pachypleurosaurs appear to have already inhabited the whole breadth of the Germanic Basin (e.g., localities Winterswijk in the North-west, Gogolin in the East, and Anisian localities on the Iberian Peninsula in the Southwest) and the Alpine Triassic region [e.g., localities in Slovenia in the Southeast and isolated vertebrae and ribs of pachypleurosaurs are documented in the S-charl-Formation (Anisian) of the Ducan region]. As such, dispersal routes are difficult to discern, as pachypleurosaurs remain present in the Ladinian in Germany, Spain, and in the Alpine region (Austria, Switzerland, and Italy). In the Early Ladinian, six pachypleurosaur species (*S. mirigiolensis*, *N. pusillus*, *N. peyeri*, *N. edwardsii*, *N. toeplitschi*, and *P. scheffoldi* nov. gen. et sp.) are documented from the Alpine Triassic, indicating that this was a ‘hot spot’ of European pachypleurosaur diversity and at least *N. pusillus* shows a more widespread distribution also in the localities of the central Muschelkalk Basin. The latest pachypleurosaur occurrence in Europe is documented by the Late Ladinian to possible Early Carnian remains in Vilanova de la Sal locality on the Iberian Peninsula (Fortuny et al., 2011). The decline of pachypleurosaurs in northcentral Europe coincides with the receding Muschelkalk Sea starting in the northern parts of Europe, and subsequent territorialization of northwestern and central Europe during the Ladinian and Early Carnian (e.g., Mckie & Williams, 2009).

Renesto et al. (2014) designed a scenario in which ancestors of *Dactylosaurus* and *Anarosaurus* immigrated from the eastern Palaeotethys into the Germanic Basin in the Olenekian/earliest Anisian. After they had diversified during the Anisian, they invaded—according to Renesto et al. (2014)—the earliest intraplatform basins of the South Alpine realm as evidenced by the presence of the Late Anisian *Odoiporosaurus* and gave then rise to the *Neusticosaurus* spp. and *Serpianosaurus* clade. Members of this clade then re-invaded the Germanic Basin during a transgression, as shown by the occurrence of *N. pusillus*. *N. pusillus* or a close relative occurs in high individual numbers in the Early Ladinian of the Germanic Basin (southern Germany; Hoheneck [Seeley, 1882; Rieppel & Lin, 1995] and various localities around the Muschelkalk/Keuper boundary, i.e., Grenzbonebed Klein & Surmik, 2021).

Based on stratigraphic data and the results of their phylogenetic analysis, Xu et al. (2022) proposed that Pachypleurosauridae (European taxa plus *Qianxisaurus*, *Honghesaurus*, and *Wumengosaurus*) may have originated in the western Tethys and from there migrated into the eastern Tethys. However, the superfamily Pachypleurosauroidea (the majority of Chinese taxa; see Xu et al., 2022) likely originated in the eastern Tethys. This concept would include the migration of an ancestor of Pachypleurosauridae or of Pachypleurosauroidea into the western Tethys as early as the Early Anisian or even already in the Olenekian, which is, however, not further discussed by Xu et al. (2022).

Documented in the fossil record is that the Early to Middle Anisian taxa, *Anarosaurus* spp. and *Dactylosaurus gracilis*, are so far restricted to the Germanic Basin (Rieppel, 2000). *Odoiporosaurus teruzzii*, which is in 32 always found basal (Renesto et al., 2014; Lin et al., 2021; this study)—although in varying positions and relationships—and *Proneusticosaurus* both differ in morphology from *Anarosaurus* spp. and *Dactylosaurus,* whereas they fit well with the morphology of the *Neusticosaurus* spp. and *Serpianosaurus* clade. Thus, *Proneusticosaurus* and the stratigraphic younger *Odoiporosaurus* are likely more basal when compared to the *Neusticosaurus* spp. and *Serpianosaurus*, whereas *Anarosaurus* spp. and *Dactylosaurus gracilis* seem to represent an isolated clade within the Germanic Basin.

## Conclusions

Eight specimens of pachypleurosaurs from the Prosanto Formation can unequivocally be assigned to a new taxon, *Prosantosaurus scheffoldi* gen. et spec. nov., based on morphology and phylogenetic analysis. The new taxon shows a mixture of plesiomorphic and apomorphic characters that supports the position as sister taxon to the clade that includes *Neusticosaurus*, *Serpianosaurus* and *Proneusticosaurus*. Finding unambiguous morphological and morphometric characters for pachypleurosaurs is largely hampered by intraspecific and ontogenetic variation, by sexual dimorphism as well as by functional (i.e., secondary aquatic adaptations) similarities and differences in preservation (e.g., compaction or dorsal vs. ventral view). Thus, to a large degree, character combinations distinguish pachypleurosaur taxa and incomplete specimens are often not assignable to a taxon. Tooth replacement of a European pachypleurosaur is described for the first time and shows a similar horizontal replacement pattern as was described in *Nothosaurus* and *Cymatosaurus*, but differing from that described for the Chinese pachypleurosaur *Keichousaurus*.

## Supplementary Information

**Additional file 1: I. Figures S1–S31. II. Character description. III. Data matrix.**

## Data Availability

All here mentioned specimens assigned to the new taxon are stored at the Palaeontological Institute and Museum, University of Zurich, Zurich.
