# Supplementary material for: A new pachypleurosaur from the Early Ladinian Prosanto Formation in the Eastern Alps of Switzerland
Source: Swiss J Palaeontol. 2022 Jul 13;141(1):12. doi: 10.1186/s13358-022-00254-2 (PMC9276568; doi:10.1186/s13358-022-00254-2)
Supplement: Supplementary file 1 — Additional file 1: I. Figures S1–S31. II. Character description. III. Data matrix. [file 13358_2022_254_MOESM1_ESM.pdf]

I. Supplementary Figures

Fig. S1. Holotype of *Prosantosaurus scheffoldi* gen. et spec. nov. (PIMUZ A/III 1274), nearly complete skeleton in dorsal view (both lower forelimbs are covered by the trunk region; posterior part of tail is missing) from Ducanfurgga 4, Davos Sertig, Canton of Grisons, south-eastern Switzerland. Specimen was coated with ammonium chloride for the photograph. **A**, complete specimen as preserved in dorsal view; **B**, skull in dorsal view; **C**, shoulder girdle and anterior trunk region; **D**, dorsal vertebrae; **E**, sacral region; **F**, anterior tail region; **G**, left humerus; **H**, left hindlimb; **I**, right hindlimb.

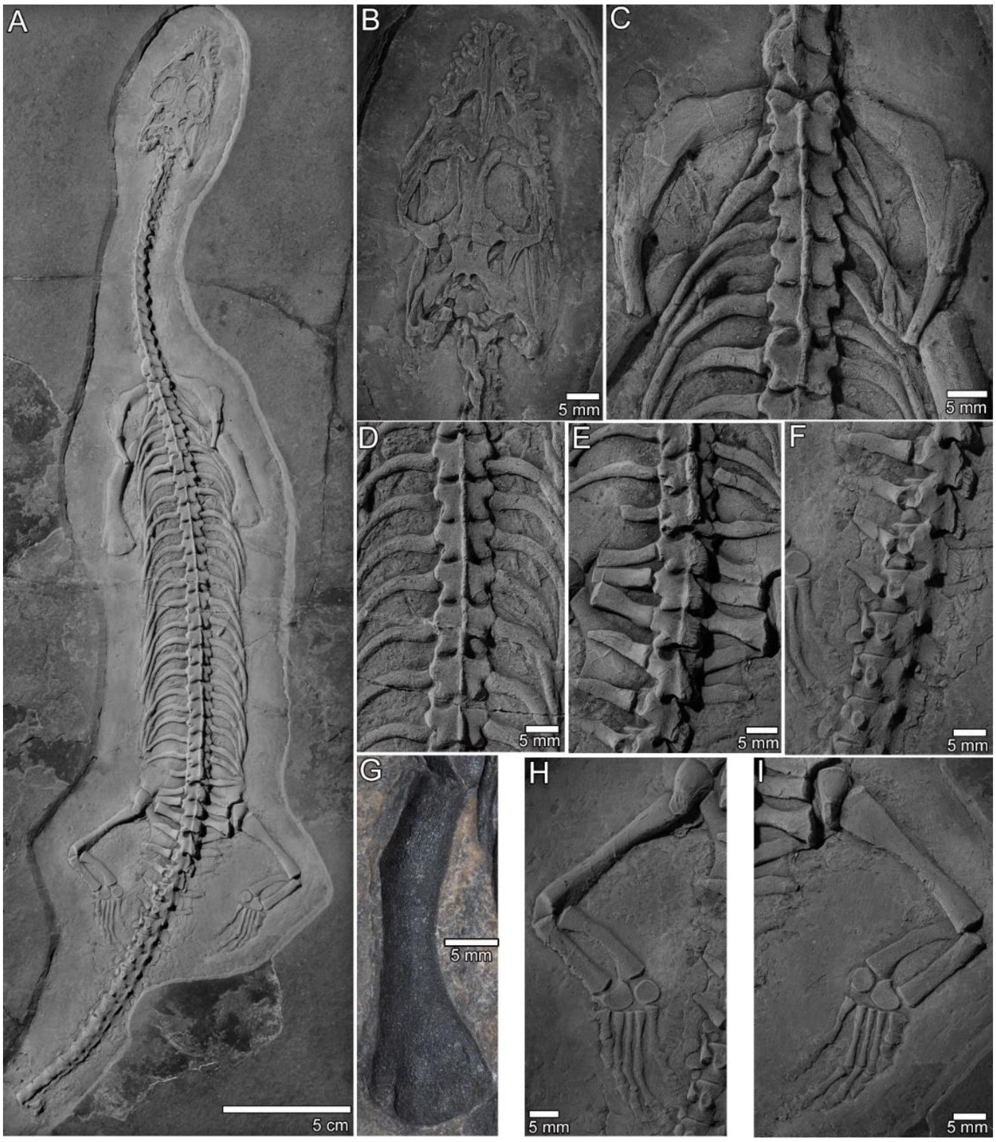

Fig. S2. Holotype of *Prosantosaurus scheffoldi* gen. et spec. nov. (PIMUZ A/III 1274), X-ray image of the entire skeleton.

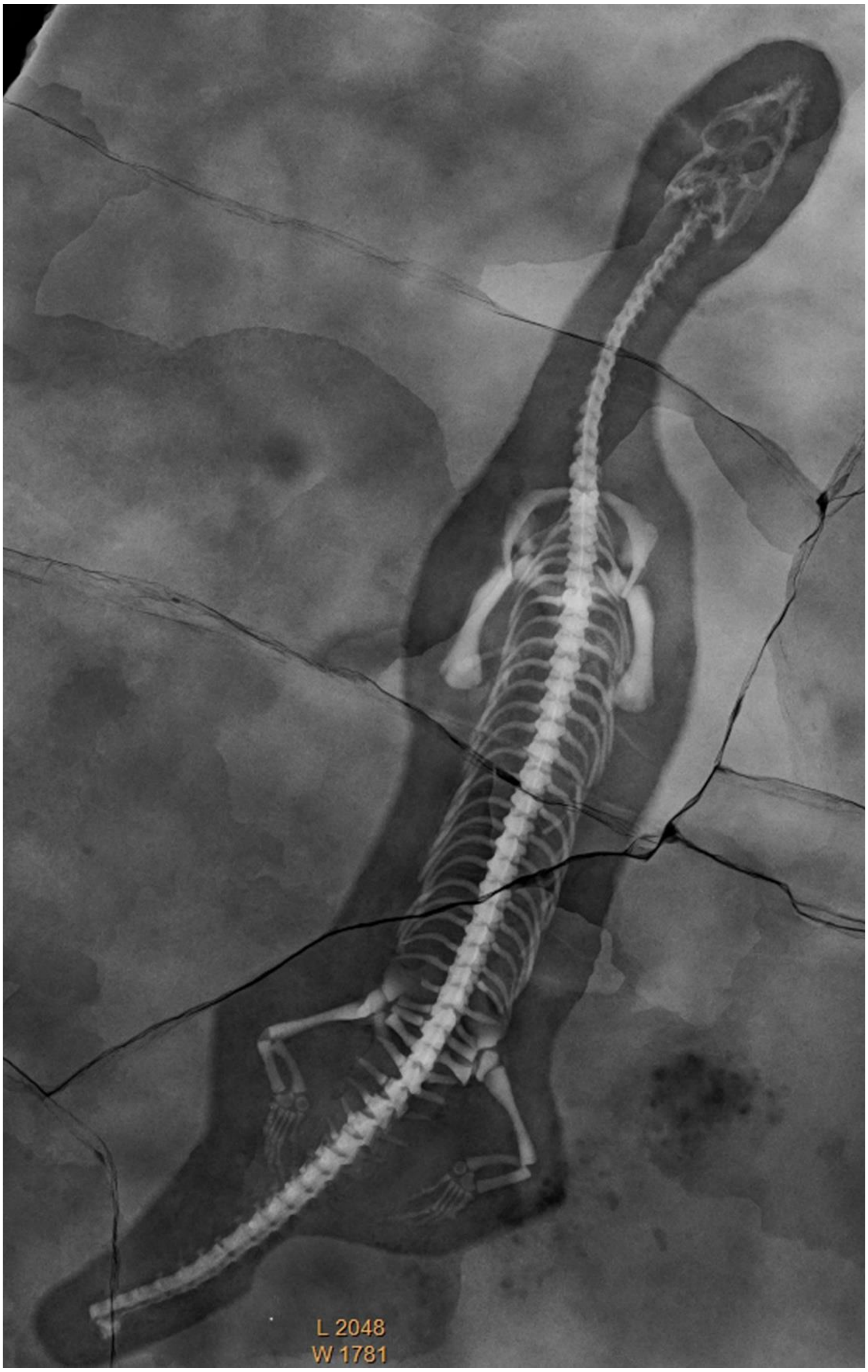

Fig. S3. Holotype of *Prosantosaurus scheffoldi* gen. et spec. nov. (PIMUZ A/III 1274). **A**, X-ray image depicting both lower arms under the trunk; **B** and **C,** photographs of the specimen depicting parts of the left radius and right metacarpals, phalanges and ulna on the slab's surface. Abbreviations: mc, metacarpal; ph, phalange; ra, radius; ul, ulna.

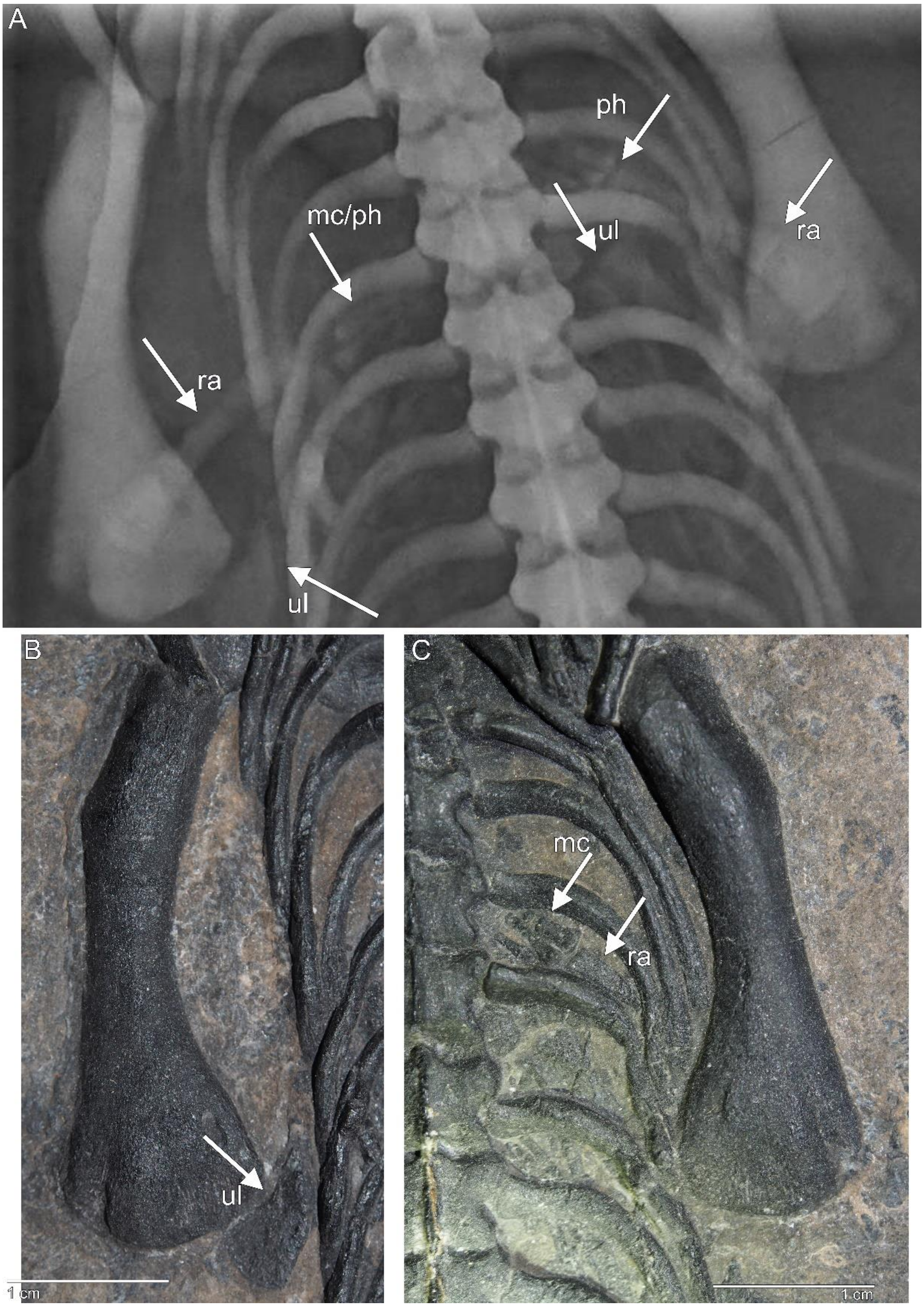

Fig. S4. Holotype of *Prosantosaurus scheffoldi* gen. et spec. nov. (PIMUZ A/III 1274). **A**, skeleton as preserved; **B**, outline sketch of entire skeleton.

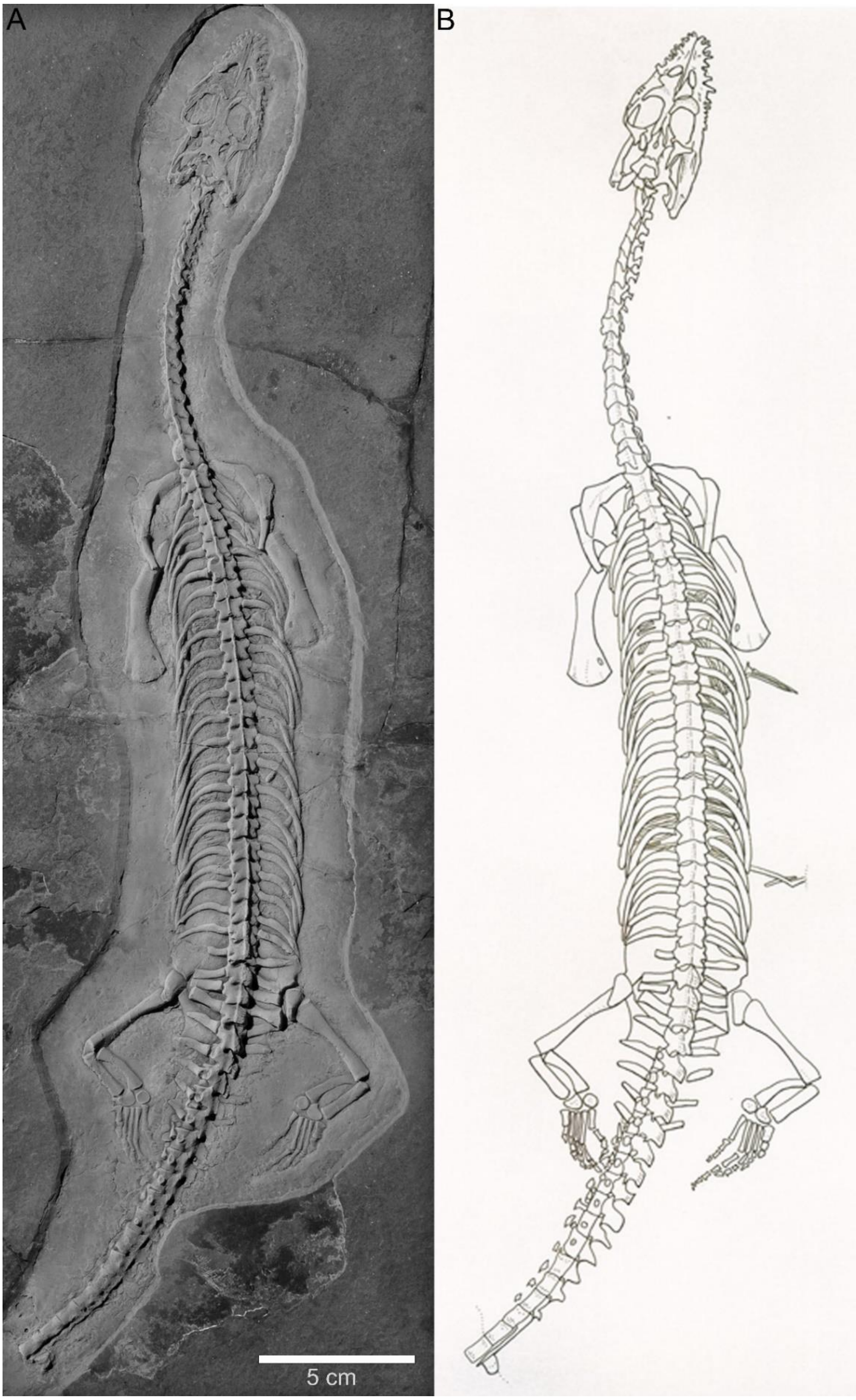

Fig. S5. Holotype of *Prosantosaurus scheffoldi* gen. et spec. nov. (PIMUZ A/III 1274). **A**, detail of skull with elements labelled and original skull in the background; **B,** interpretative outline sketch of skull elements; **C,** X-ray image of the skull. Abbreviations as in figure 2 in the main text.

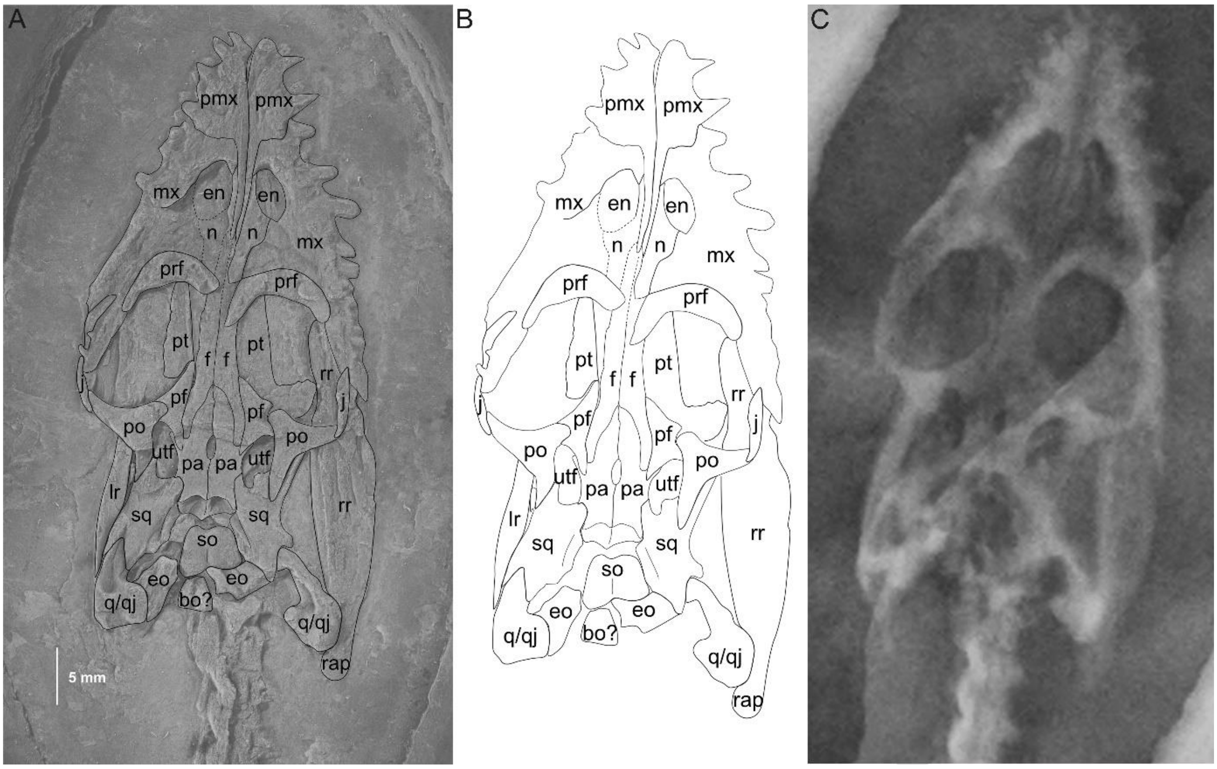

Fig. S6. *Prosantosaurus scheffoldi* gen. et spec. nov. (PIMUZ A/III 668), incomplete skeleton in dorsal view (parts of right body half and posterior tail are missing) Ducantal-Chachlengstell, Davos Sertig, Canton of Grisons, south-eastern Switzerland. Specimen was coated with ammonium chloride for the photograph. **A,** complete specimen as preserved; **B,** skull; **C,** left humerus; **D,** left radius and ulna; **E,** right manus; **F,** part of the tail vertebrae in lateral view; **G,** dorsal ribs in the left trunk region with gastral elements in between; **H,** sacral ribs; **I,** sacral ribs and partially preserved left pelvic girdle.

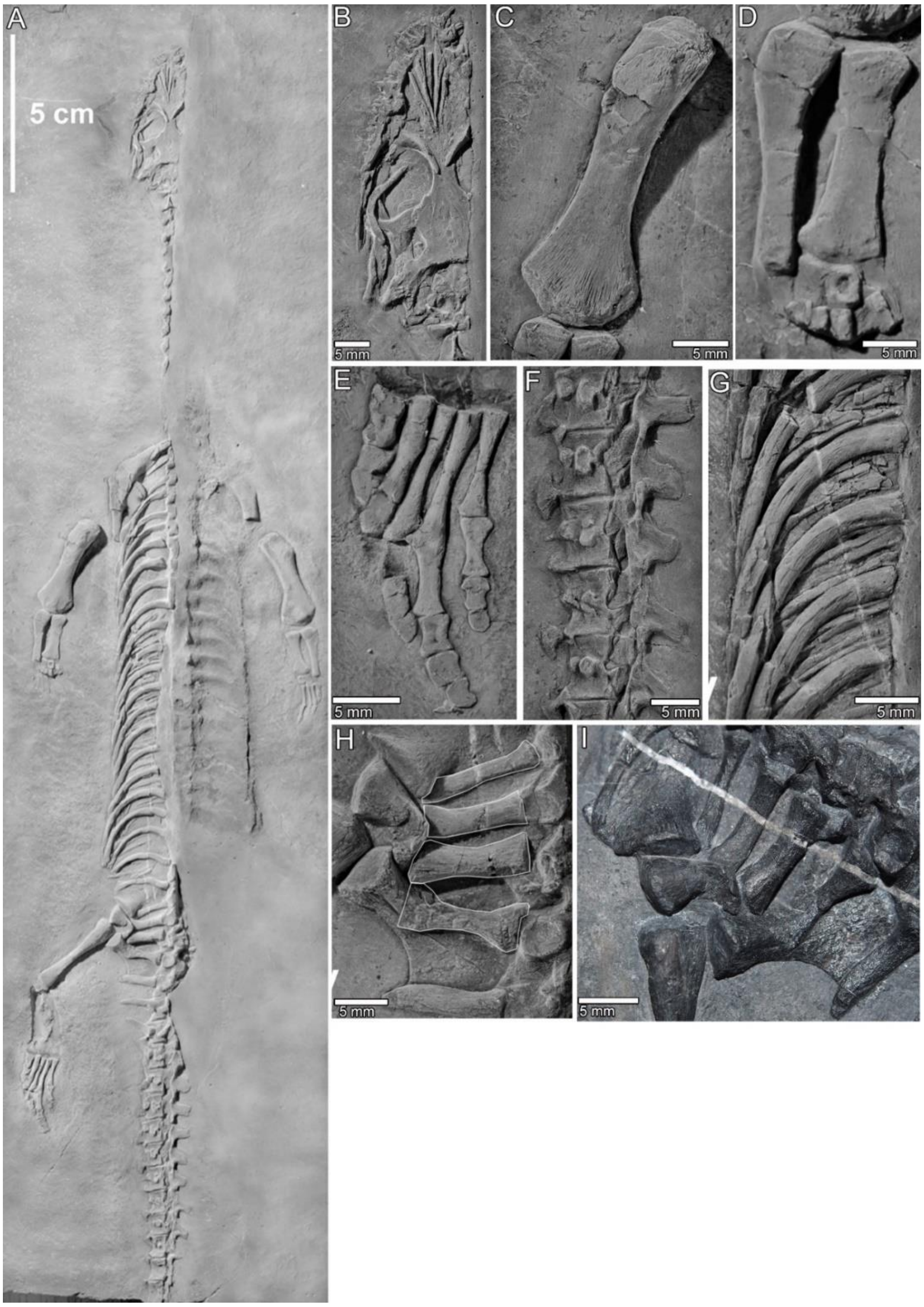

Fig. S7. *Prosantosaurus scheffoldi* gen. et spec. nov. (PIMUZ A/III 1197), nearly complete skeleton in dorsolateral view from Ducanfurgga 4, Davos Sertig, Canton of Grisons, south-eastern Switzerland. Specimen was coated with ammonium chloride for the photograph. **A,** lower jaws and skull in ventral view; **B,** complete specimen as preserved; **C,** cervical vertebrae in ventral view; **D,** dorsal vertebrae in dorsal view; **E,** anterior part of tail, i.e., caudal vertebrae; **F,** mid- part of tail; **G,** posterior part of tail; **H,** disarticulated shoulder girdle; **I,** right humerus; **J,** right femur; **K,** right stylo- and autopodium; **L,** left stylo- and autopodium.

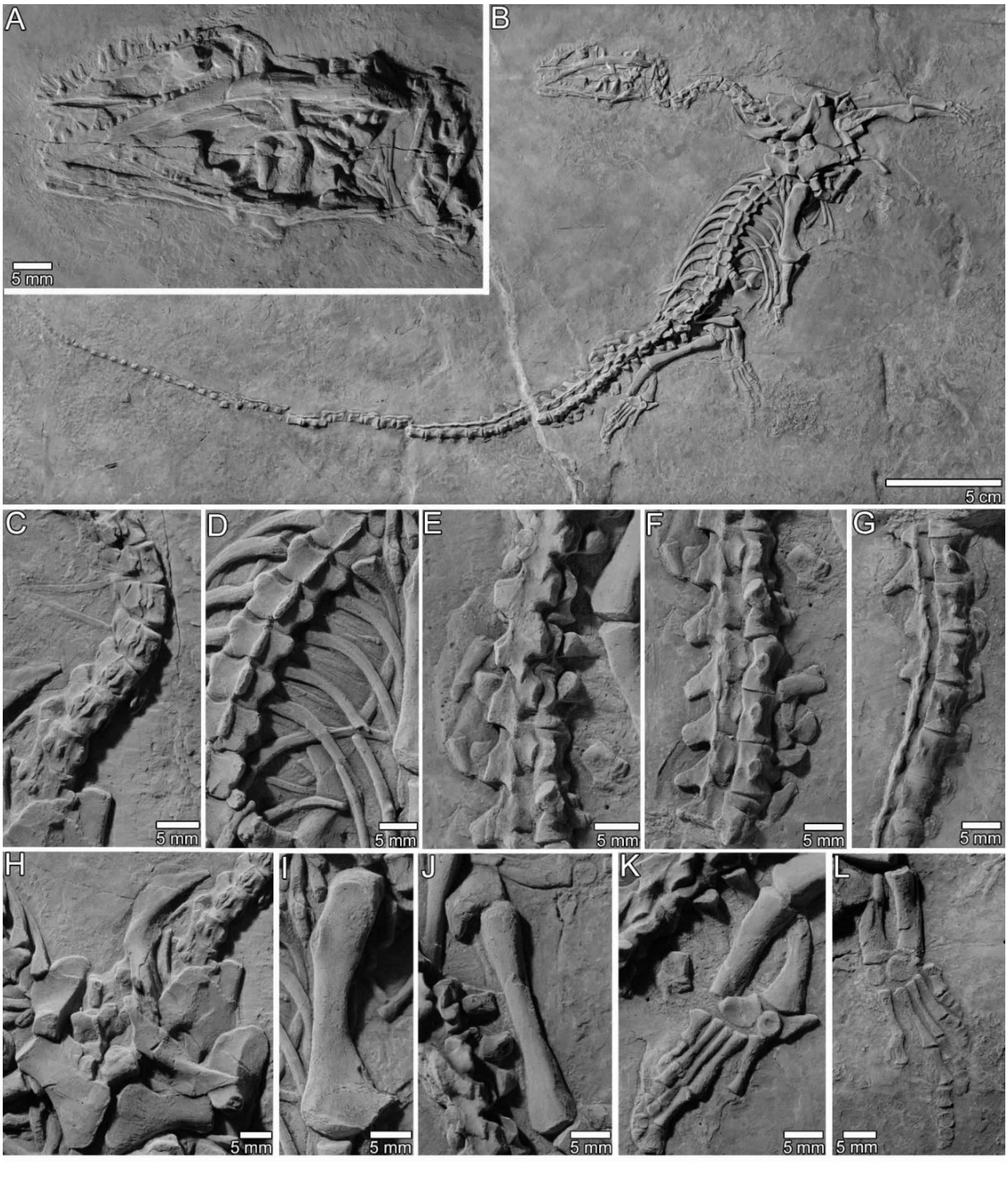

Fig. S8. *Prosantosaurus scheffoldi* gen. et spec. nov. (PIMUZ A/III 1197). **A,** original specimen as preserved, and **B,** detail of skull; C, outline sketch of skull elements; D, outline sketch of entire specimen.

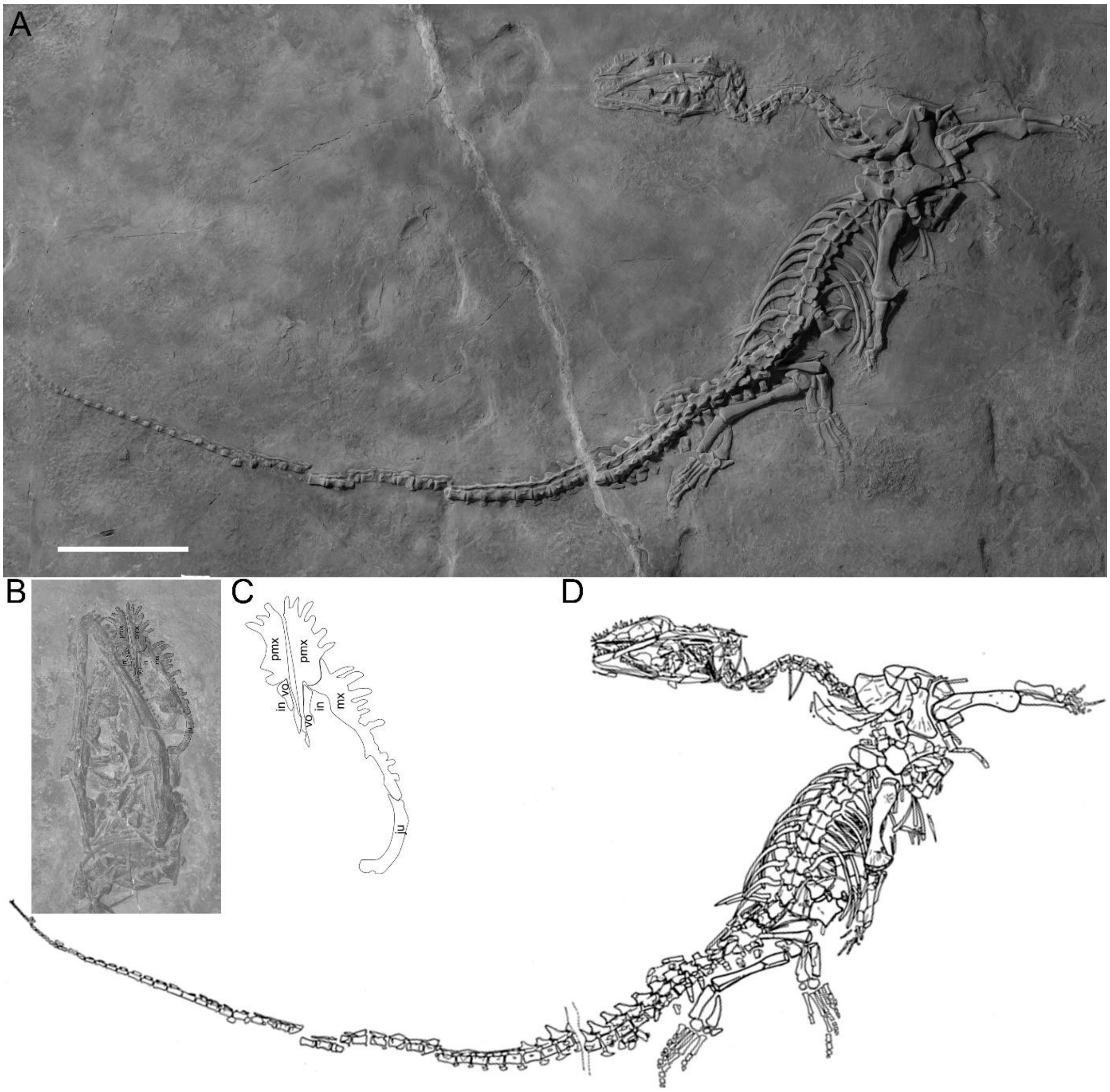

Fig. S9. *Prosantosaurus scheffoldi* gen. et spec. nov. (PIMUZ A/III 1240), nearly complete skeleton in dorsolateral view with the tip of the snout and the tip of the tail missing from Ducanfurgga 4, Davos Sertig, Canton of Grisons, south-eastern Switzerland. Specimen was coated with ammonium chloride for the photograph. **A,** complete specimen as preserved; **B,** skull with the anterior part missing; **C,** left humerus; **D,** left forelimb and half of trunk exposing dorsal ribs and gastral elements; **E,** left hindlimb; **F,** sacral region.

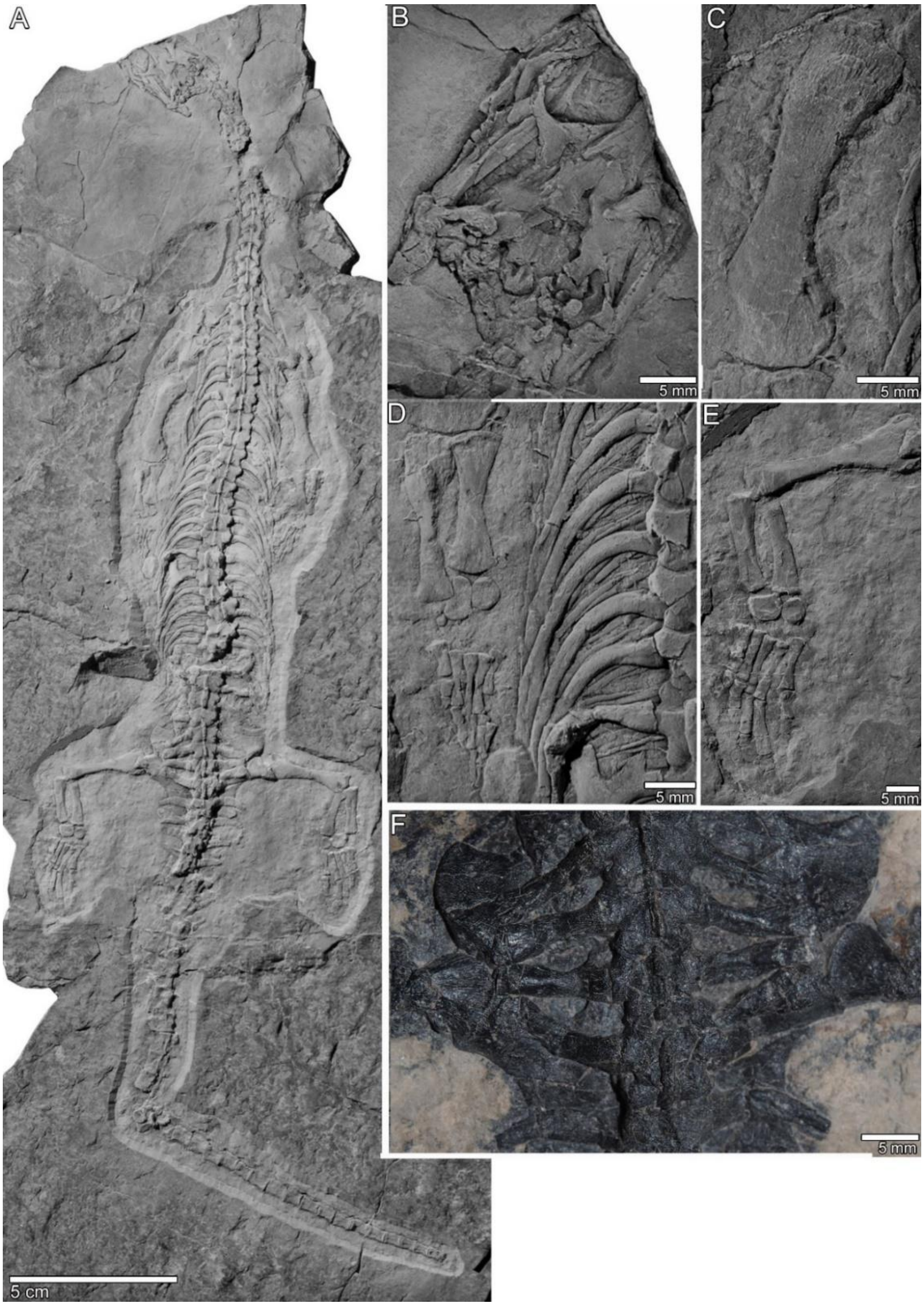

Fig. S10. *Prosantosaurus scheffoldi* gen. et spec. nov. (PIMUZ A/III 4566), nearly complete skeleton in ventral view (posterior part of tail is missing) from Ducanfurgga 4, Davos Sertig, Canton of Grisons, south-eastern Switzerland. Specimen was coated with ammonium chloride for the photograph. **A,** complete specimen as preserved; **B,** shoulder girdle; **C,** trunk region in ventral view exposing gastralia; **D,** pelvic region; **E,** skull; **F,** right humerus; **G,** left humerus (photograph taken from the cast, not from the original skeleton). **H,** left hindlimb.

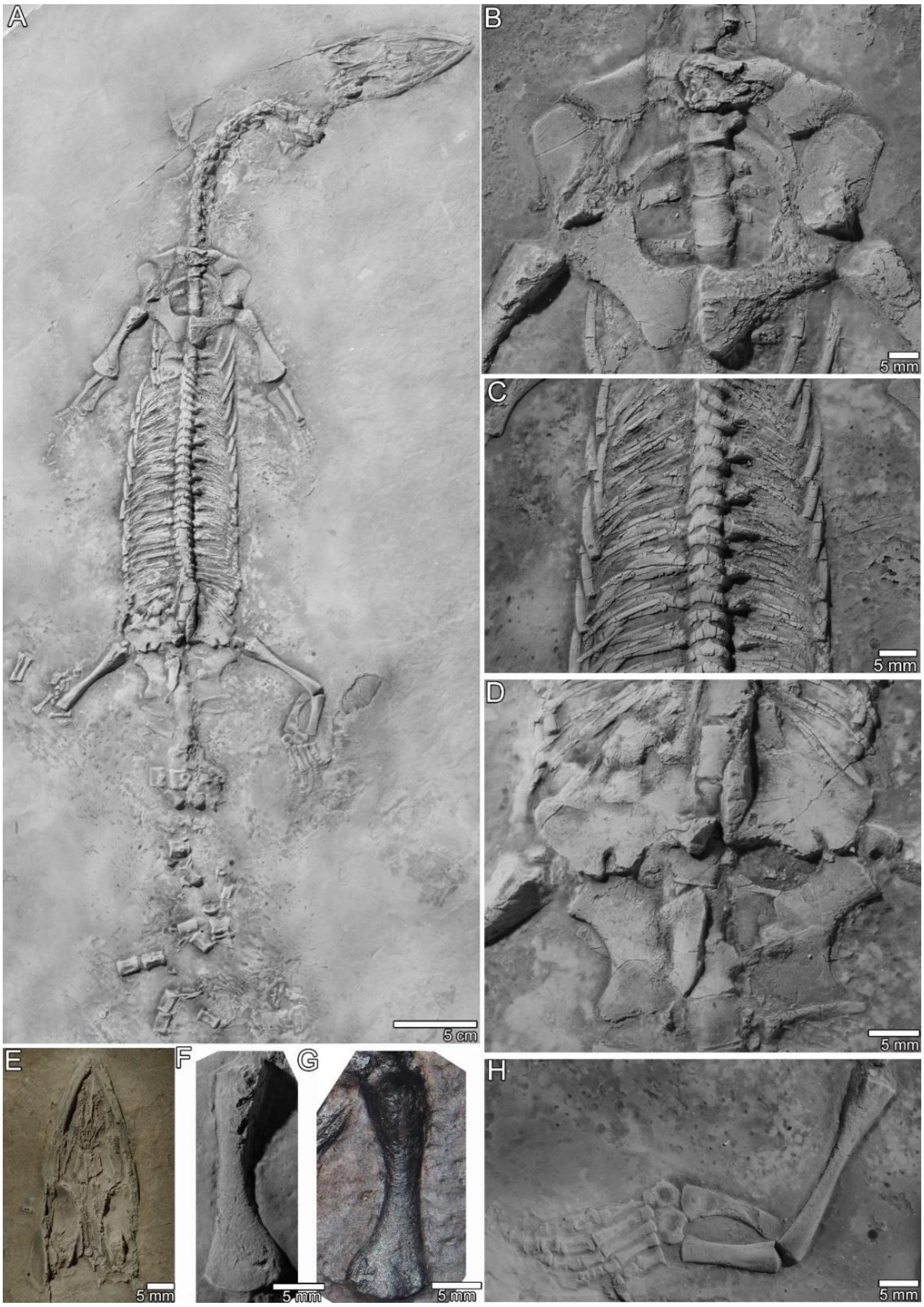

Fig. S11. *Prosantosaurus scheffoldi* gen. et spec. nov. (PIMUZ A/III 1275), nearly complete skeleton in ventral view (posterior part of tail is disarticulated) from Ducanfurgga 4, Davos Sertig, Canton of Grisons, south-eastern Switzerland. Specimen was coated with ammonium chloride for the photograph. **A,** complete specimen as preserved; **B,** skull in ventral view; **C,** posterior cervical vertebrae; **D,** right scapula; **E,** left half of pelvic girdle (pubis, ischium, illium); **F,** right humerus; **G,** right femur; **H,** detail of gastral apparatus.

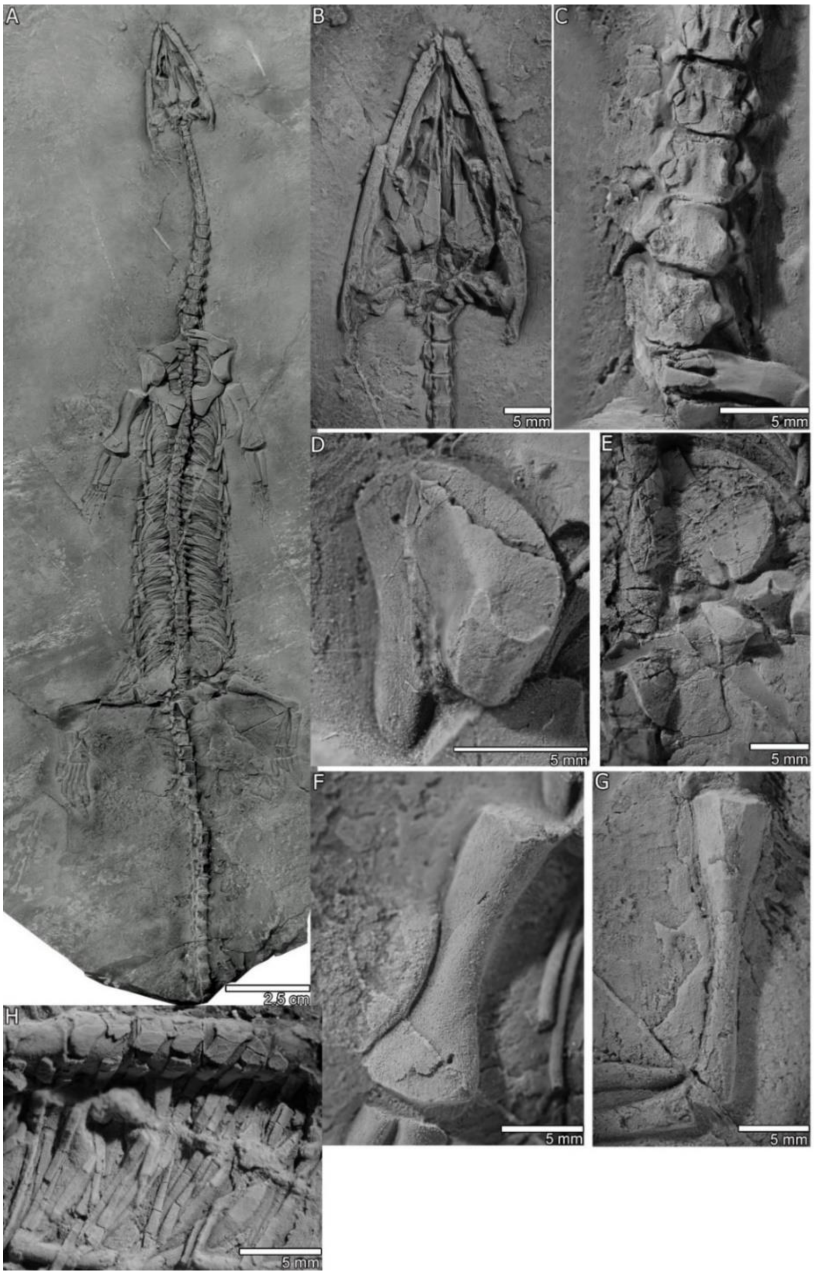

Fig. S12. *Prosantosaurus scheffoldi* gen. et spec. nov. in ventral view (PIMUZ A/III 1275). **A,** skeleton as preserved; **B,** outline sketch entire skeleton.

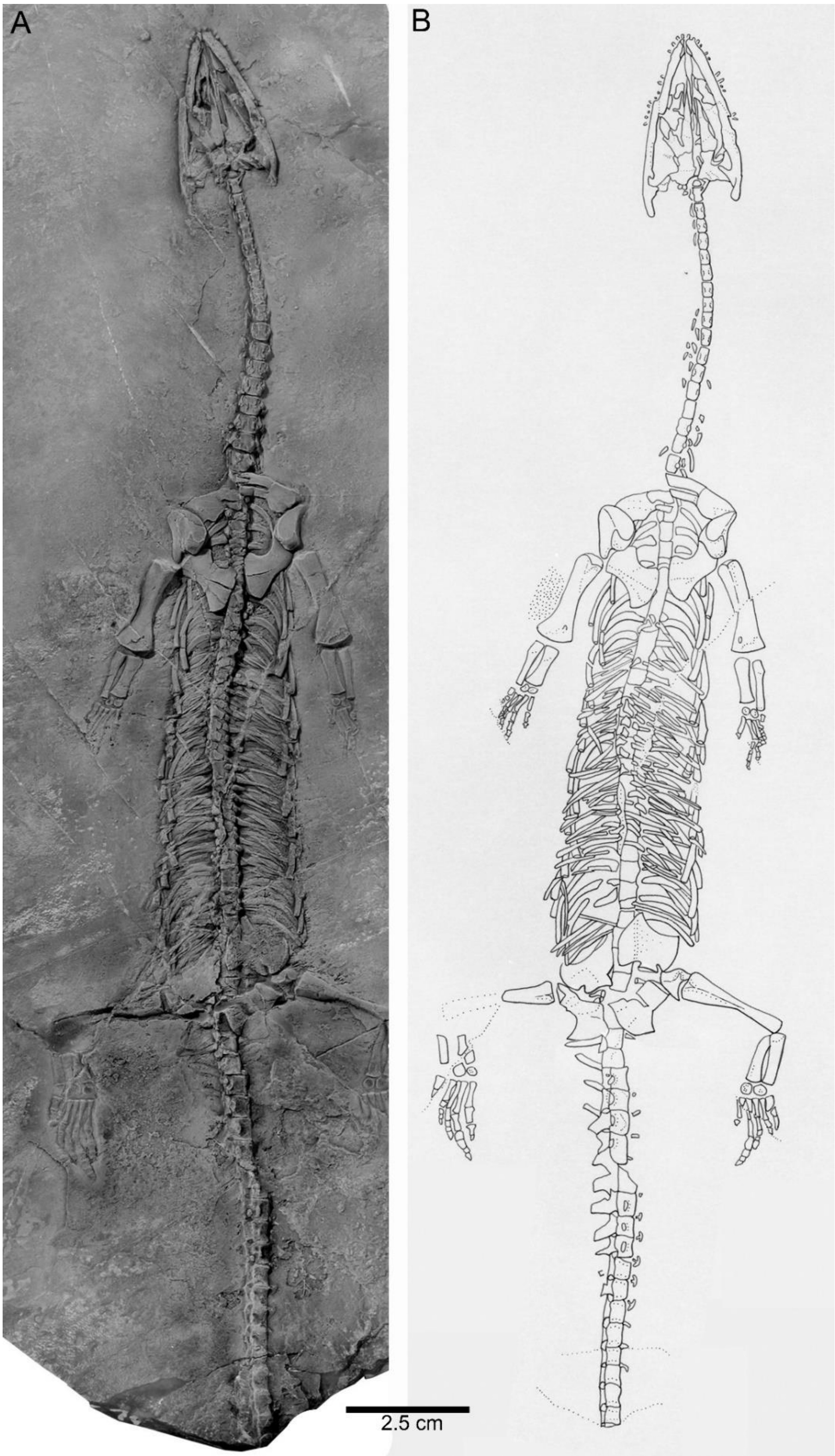

Fig. S13. *Prosantosaurus scheffoldi* gen. et spec. nov. in ventral view (PIMUZ A/III 1275). **A,** outline sketch of the skull elements in ventral view with the skull in the background; **B,** outline sketch with skull elements labelled; **C,** detail of cervical vertebral column with associated cervical ribs and shoulder girdle in ventral view. Abbreviations as in figure 2 in the main text.

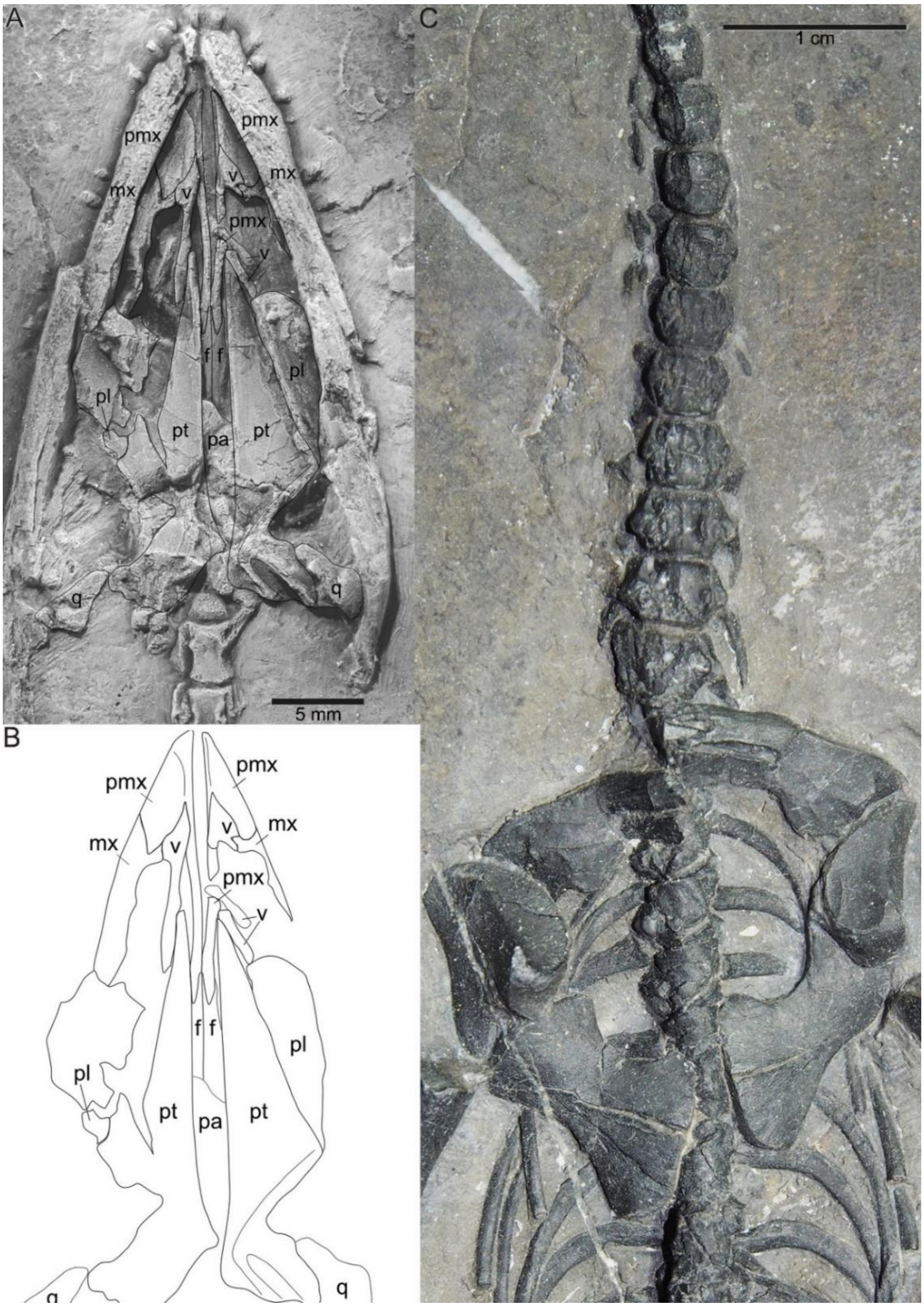

Fig. S14. *Prosantosaurus scheffoldi* gen. et spec. nov. (PIMUZ A/III 1490), incomplete skull in ventral view with disarticulated postcranium distributed over four slabs from Ducanfurgga 4, Davos Sertig, Canton of Grisons, south-eastern Switzerland. **A, E,** and **F,** are small slabs and counterslabs showing few elements of presumably the lower limb; **B,** small slab with broken-off teeth belonging to the left maxilla (see also S15B, C); **C,** slab with few elements i.e., mostly fragmented ribs and vertebrae; **D,** largest slab with many disarticulated bones, including a poorly preserved lower jaw ramus (see S15D), vertebrae and ribs, girdle elements (coracoid) and limb bones (stylopodial and zeugopodial bones).

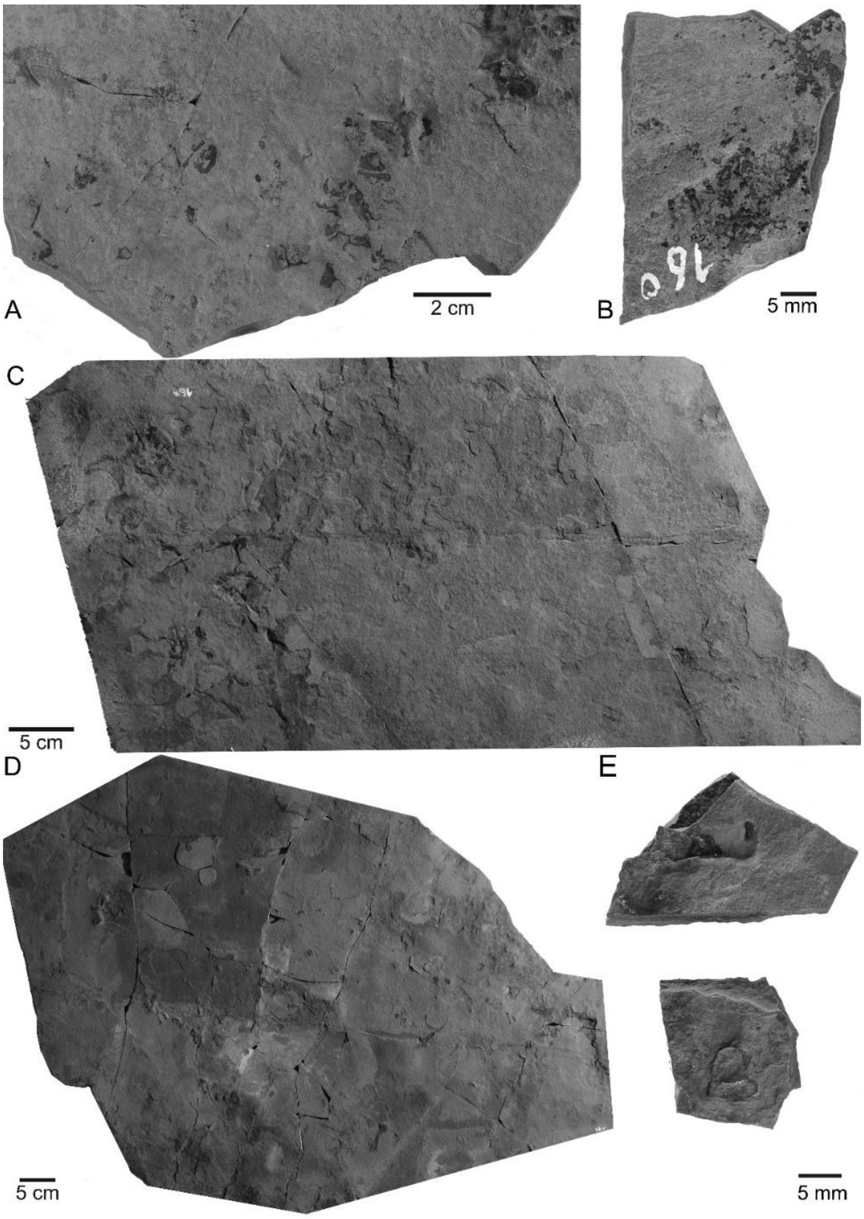

Fig. S15. *Prosantosaurus scheffoldi* gen. et spec. nov. (PIMUZ A/III 1490). **A,** detail of incomplete skull in ventral view; **B,** counterpart of left maxillary teeth; **C,** detail of teeth; **D,** lower jaw; **E,** outline sketch of the skull elements in ventral view with the skull in the background; **F,** outline sketch of the skull elements. Abbreviations as in figure 2 in the main text.

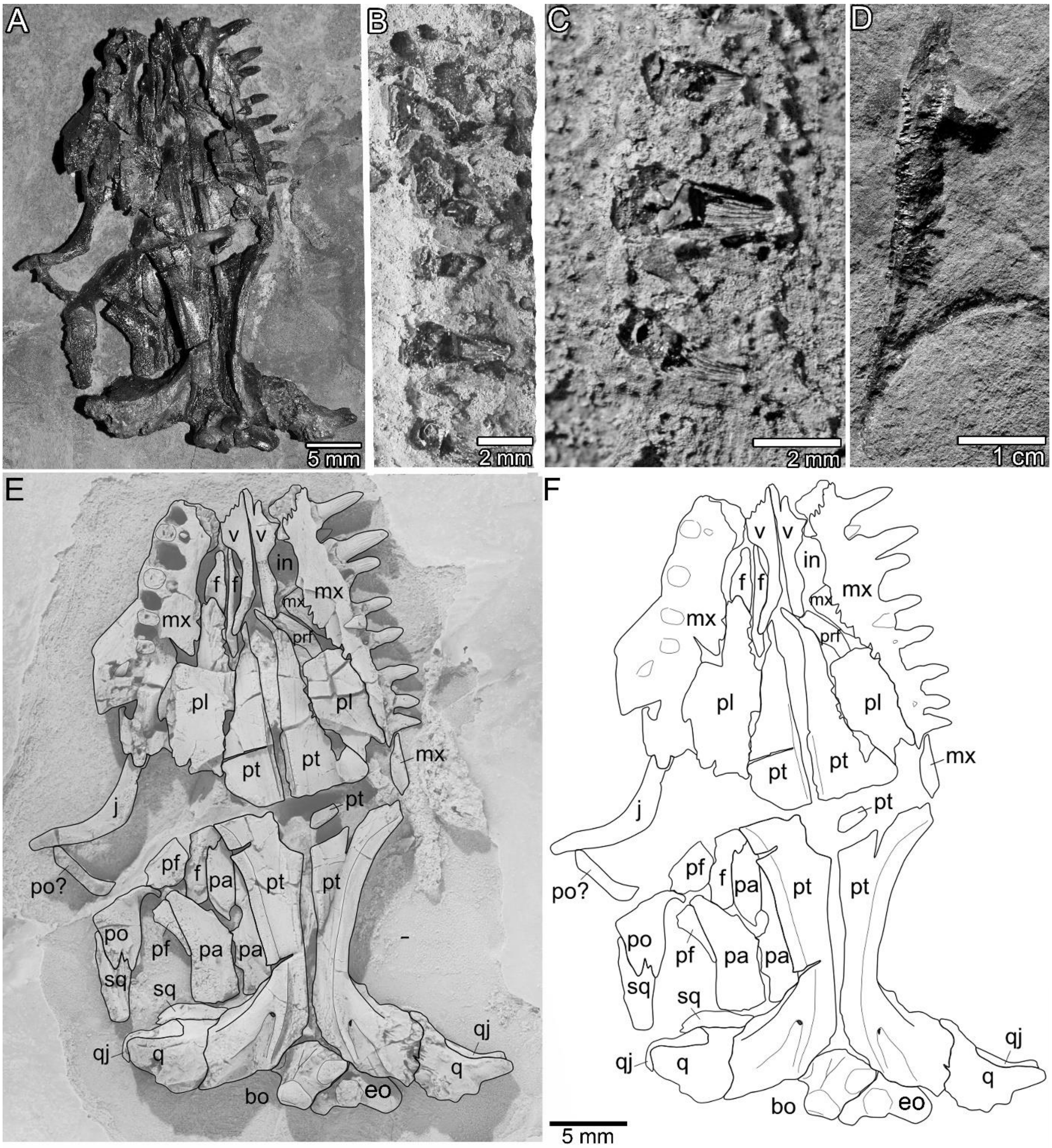

Fig. S16. *Prosantosaurus scheffoldi* gen. et spec. nov. PIMUZ A/III 1490. **A** and **B,** humeri; **C,** coracoid, ulna and radius. Due to poor preservation the orientation of each element cannot be elucidated.

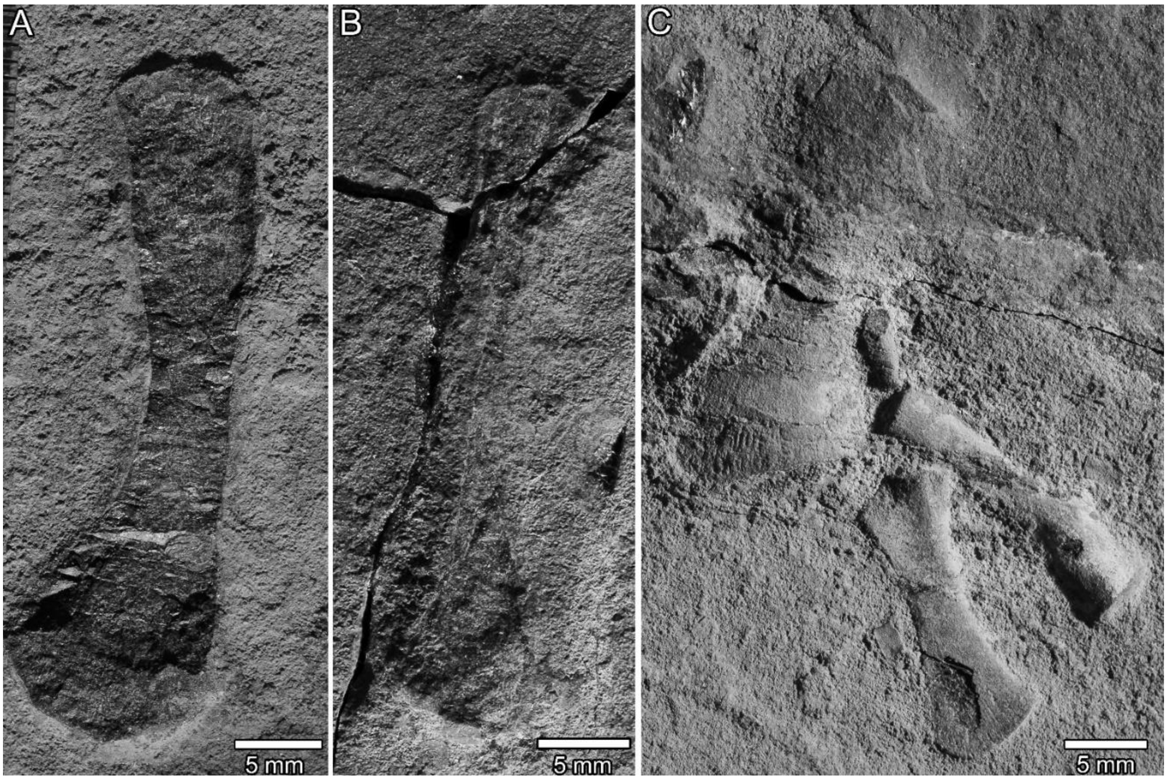

Fig. S17. *Prosantosaurus scheffoldi* gen. et spec. nov. (PIMUZ A/III 710) from Ducantal-Hungerbüel, Davos Sertig, Canton of Grisons, south-eastern Switzerland. **A,** incomplete skull in dorsal view. Specimen was coated with ammonium chloride for the photograph; **B,** outlines sketch of skull elements. Abbreviations as in figure 2 in the main text.

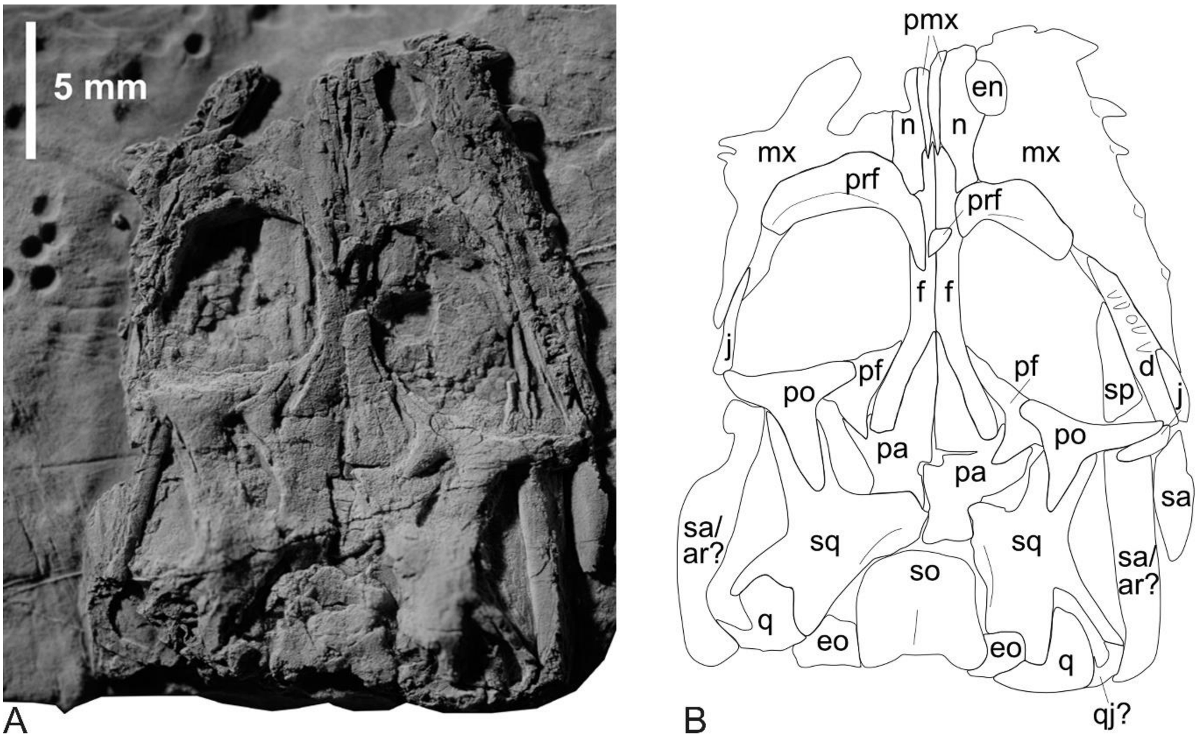

Fig. S18. Pachypleurosauria indet. (PIMUZ A/III 721), poorly preserved skull in dorsal view from Gletscher Ducan, Bergün Stugl, Canton of Grisons, south-eastern Switzerland.

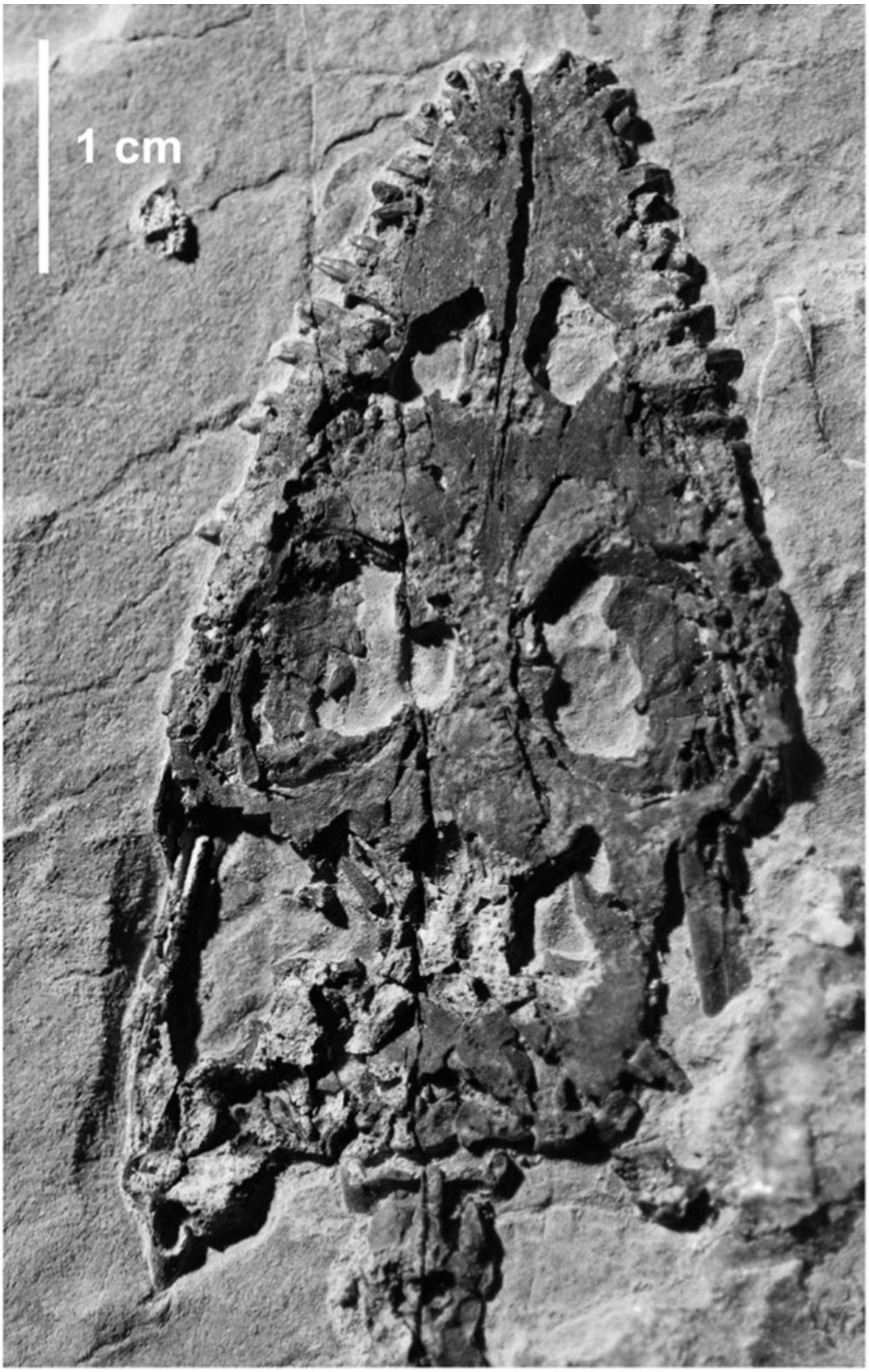

Fig. S19. Pachypleurosauria indet. (PIMUZ A/III 254), incomplete trunk region in dorsal view from the Stulseralp/Val da Stugl, Bergün Stugl, holotype of "*Pachypleurosaurus staubi*" (Kuhn-Schnyder 1959). Synonymised by Sander (1989) with *Neusticosaurus pusillus.* Specimen was coated with ammonium chloride for the photograph.

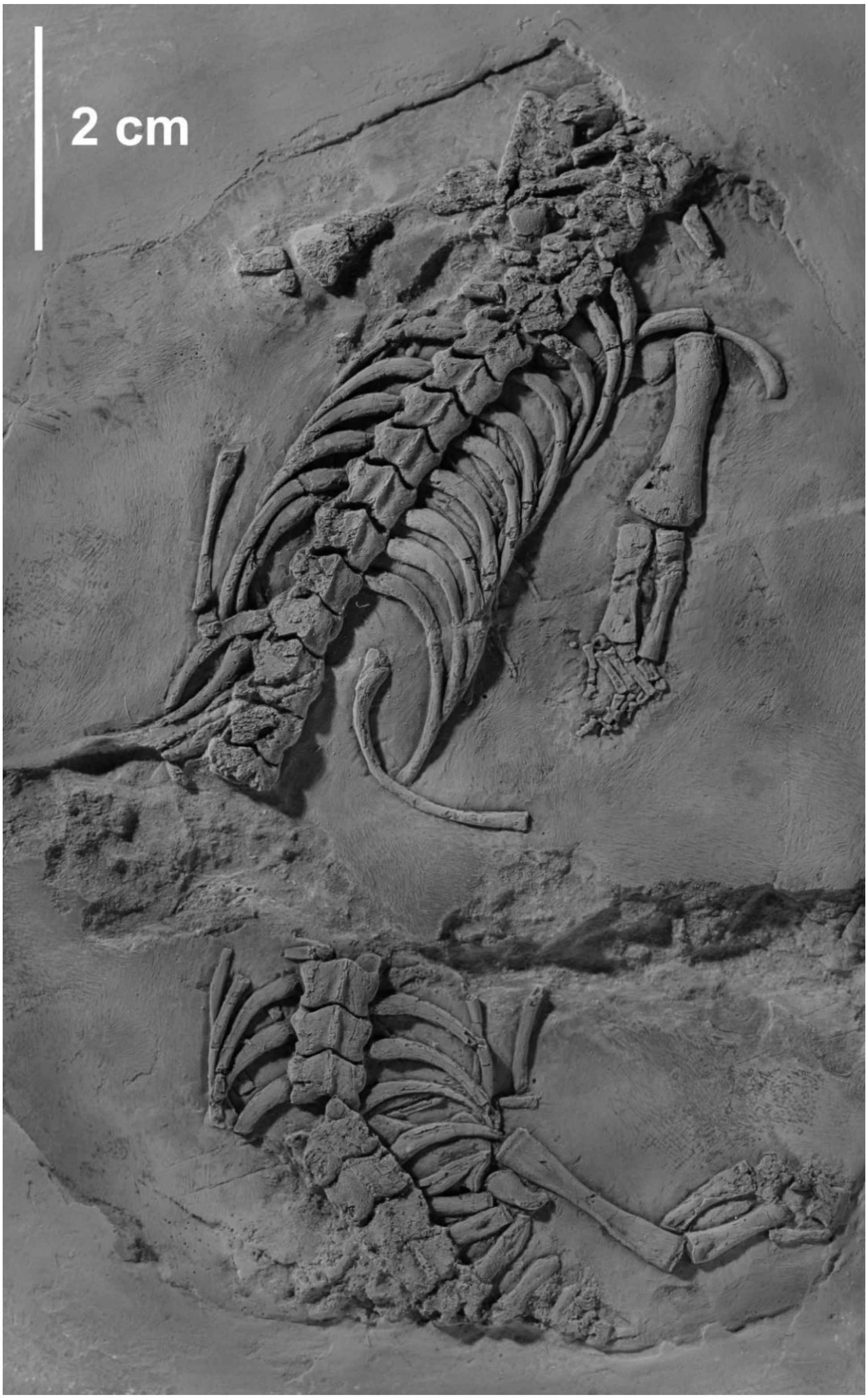

Fig. S20. Pachypleurosauria indet. (PIMUZ A/III 711), left trunk region in dorsal view from Ducanfurgga 1, Davos Sertig, Canton of Grisons, south-eastern Switzerland Specimen was coated with ammonium chloride for the photograph.

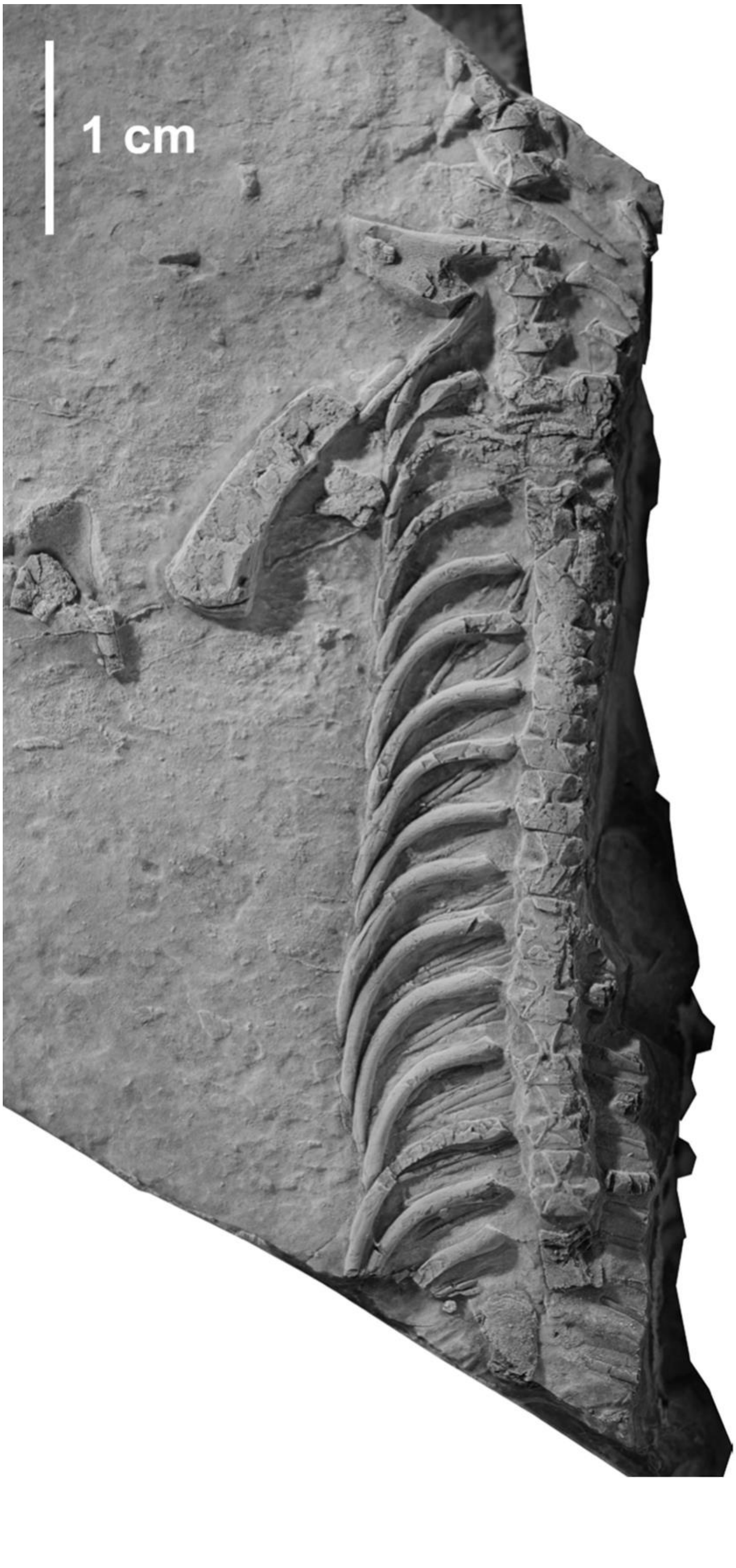

Fig. S21. Pachypleurosauria indet. (PIMUZ A/III 499), incomplete trunk region in dorsal view from the Ducantal, Davos Sertig, Canton of Grisons, south-eastern Switzerland. Specimen was coated with ammonium chloride for the photograph.

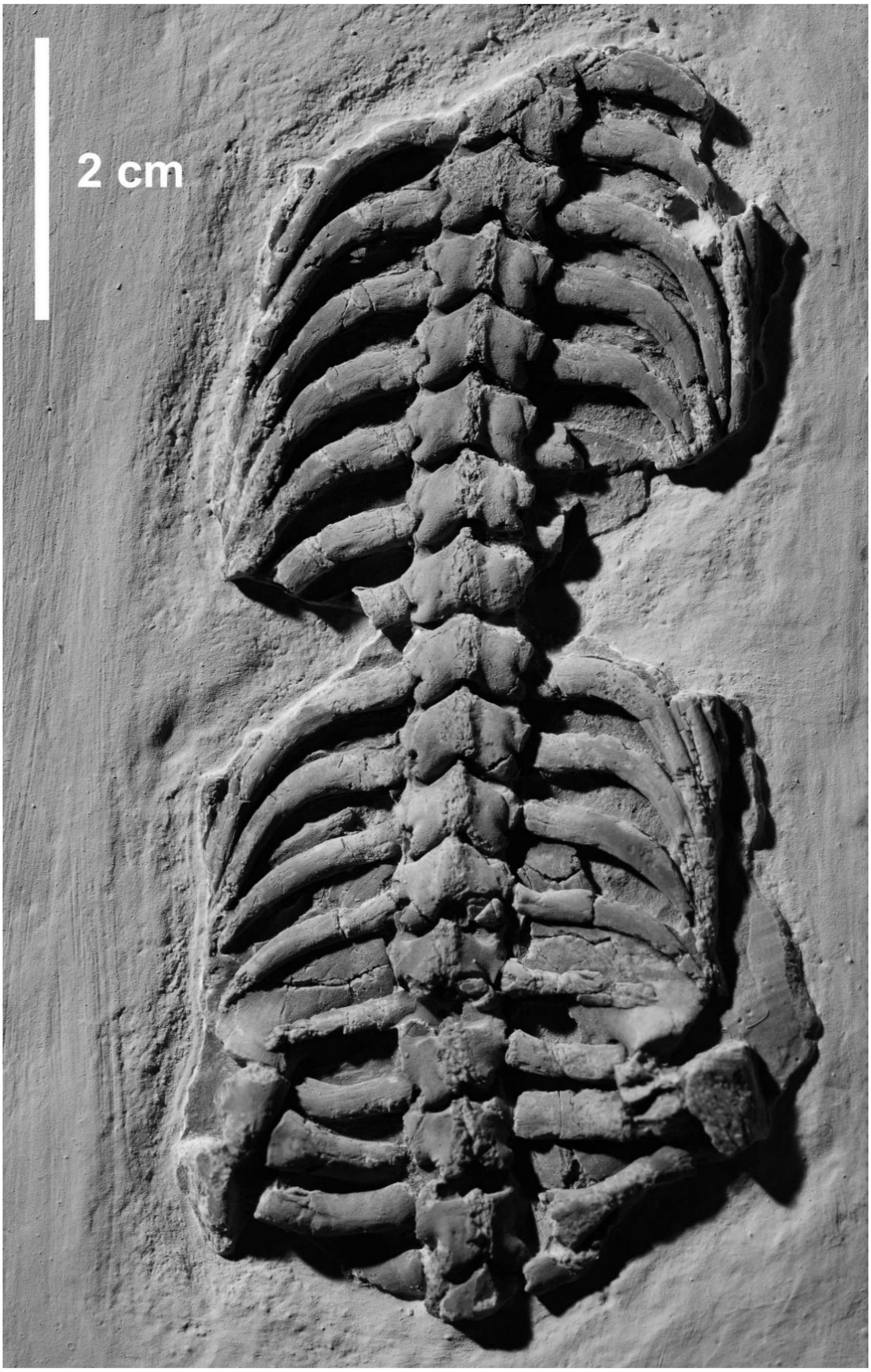

Fig. S22. Pachypleurosauria indet. (PIMUZ A/III 720), incomplete trunk region in dorsal view from Ducantal-Chachlengstell, Davos Sertig, Canton of Grisons, south-eastern Switzerland Specimen was coated with ammonium chloride for the photograph.

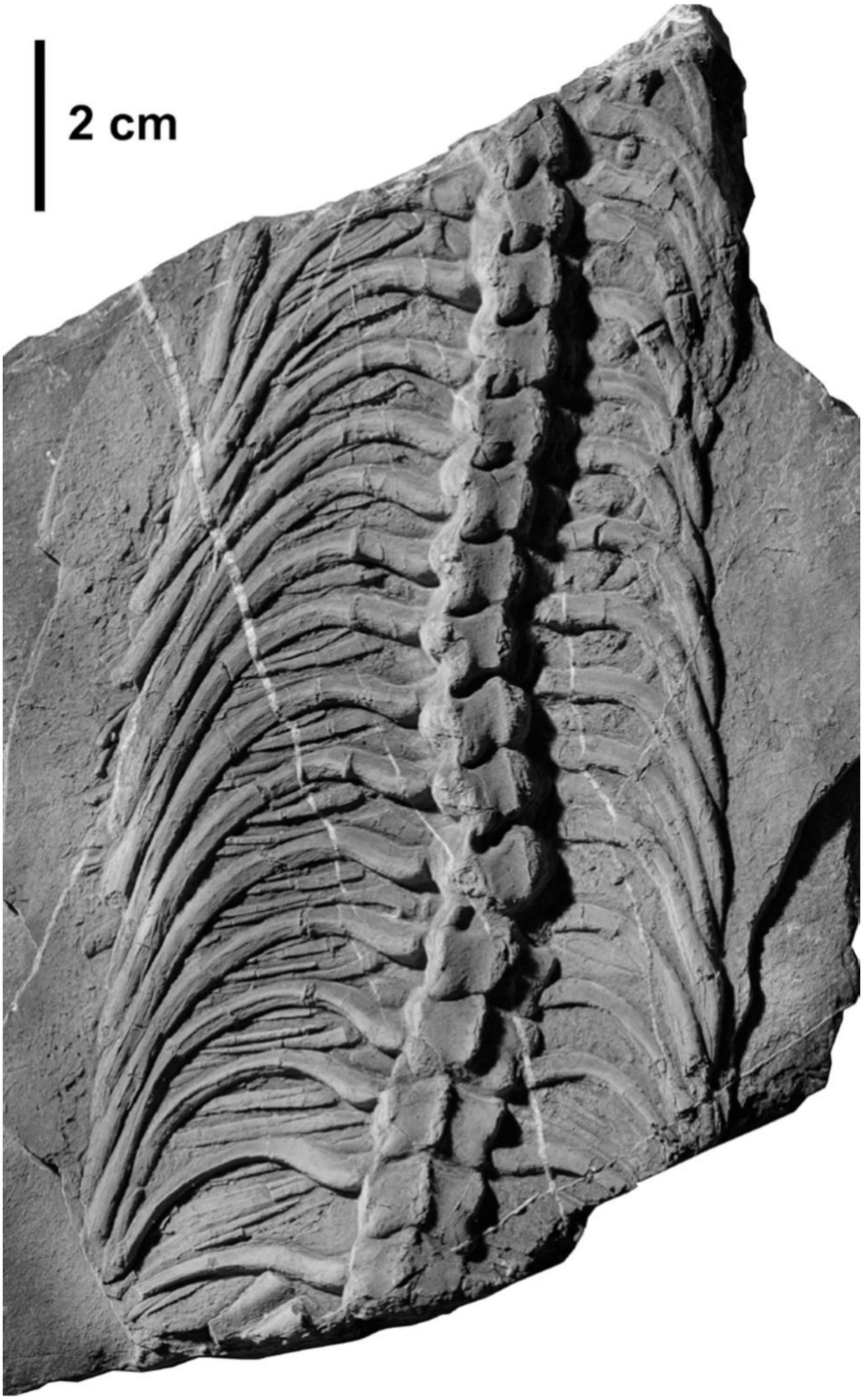

Fig. S23. Disarticulated postcranial elements of a pachypleurosaur in the stomach of *Saurichthys* sp. (PIMUZ A/I 3579) from Ducanfurgga 4, Davos Sertig, Canton of Grisons, south-eastern Switzerland.

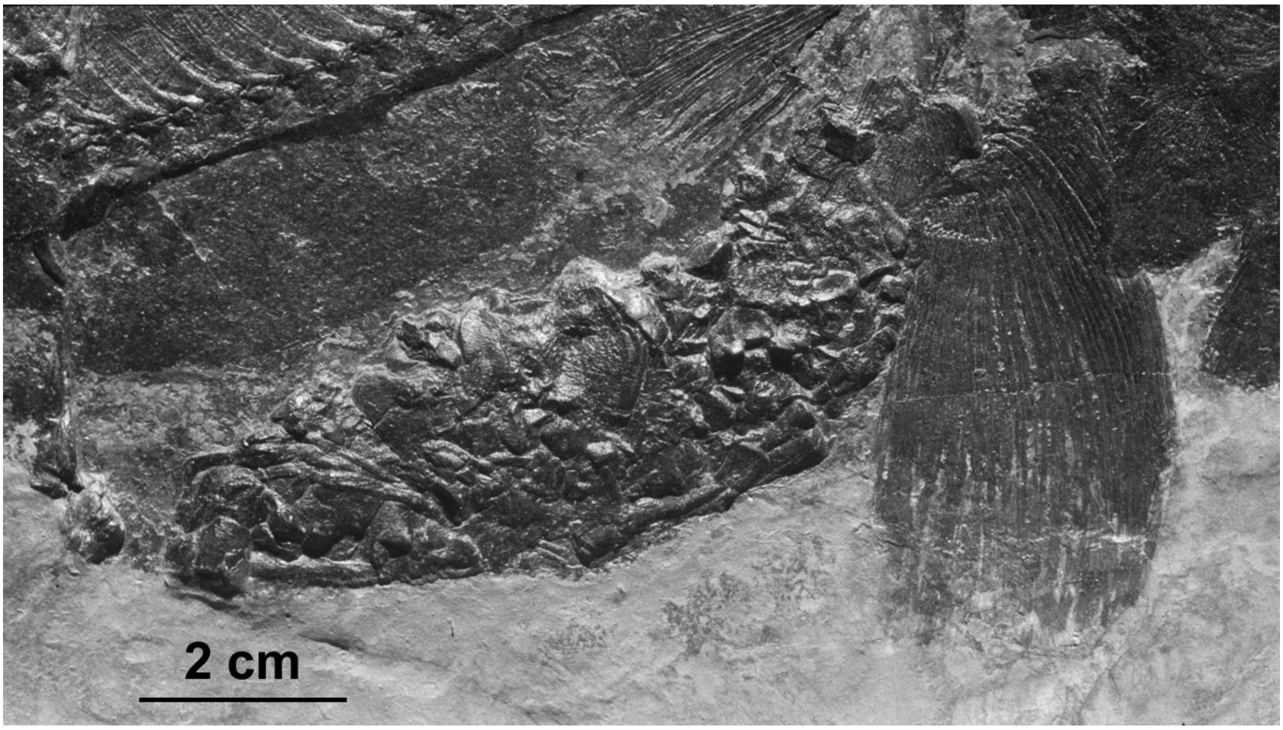

Fig. S24. Variation in humerus morphology of *Prosantosaurus scheffoldi* gen. et spec. nov. (PIMUZ A/III 1274/holotype, PIMUZ A/III 1197, PIMUZ A/III 668, PIMUZ A/III 1240, PIMUZ A/III 4566, PIMUZ A/III 1275).

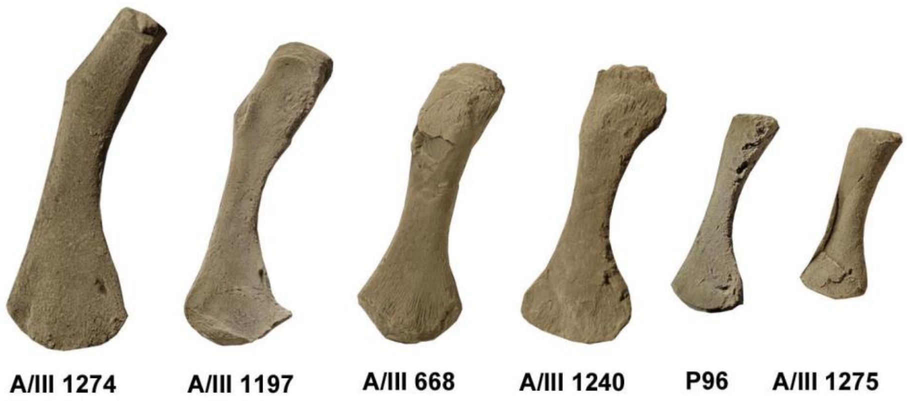

Fig. S25. Humeri of *Neusticosaurus pusillus*. **A1,** left humeri in dorsal view (PIMUZ T3740, T3519), sex X; **A2,** left humeri in dorsal view (PIMUZ T 3390, T 3614), sex Y; **B1,** right humeri in ventral view (PIMUZ T 3526, T 3530, T 3568, T 3598, T 3852), sex X; **B2,** right humeri in ventral view (PIMUZ T 3513, T 3525, T 3529, T 3551, T 3560, T 3556, T 3570), sex Y.

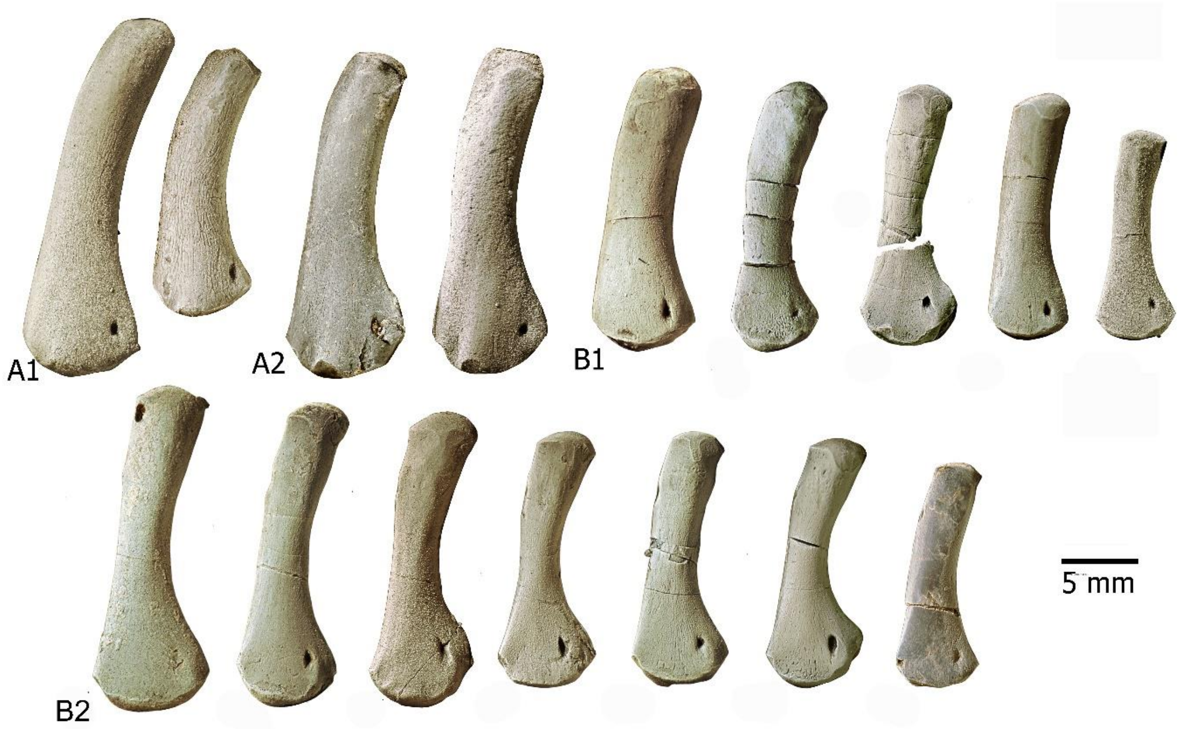

Fig. S26. Humeri of *Serpianosaurus mirigiolensis*. **A,** PIMUZ T 3682, T 3677, T 3406, T 3681, T 3709, T 490, sex Y; **B,** juvenile specimen (PIMUZ T 3810).

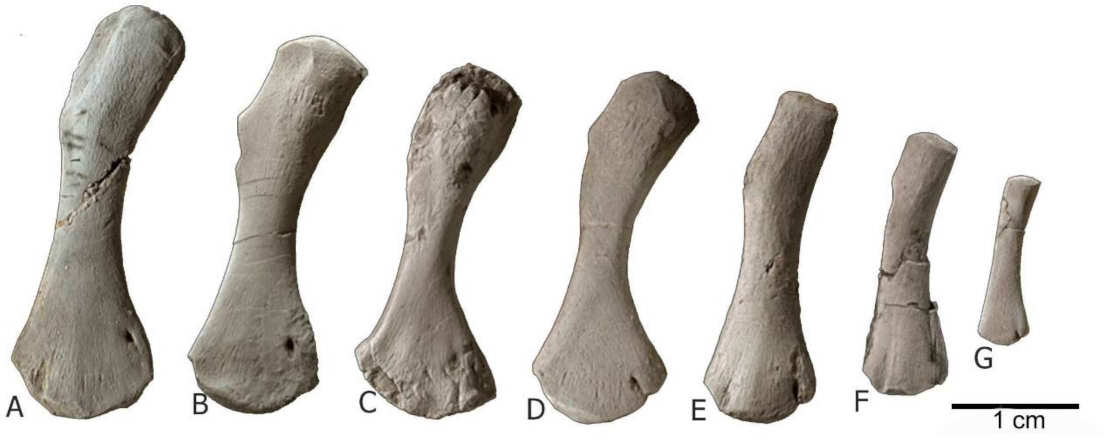

Fig. S27. Schematic palaeogeographic maps of the Germanic and Alpine Middle Triassic exhibiting European pachypleurosaur localities and their stratigraphic occurrences. Map is based on Ziegler (2005: fig. 6), Furrer (2019: fig. 129) and references therein (for abbreviations see Fig.1A in the main text).

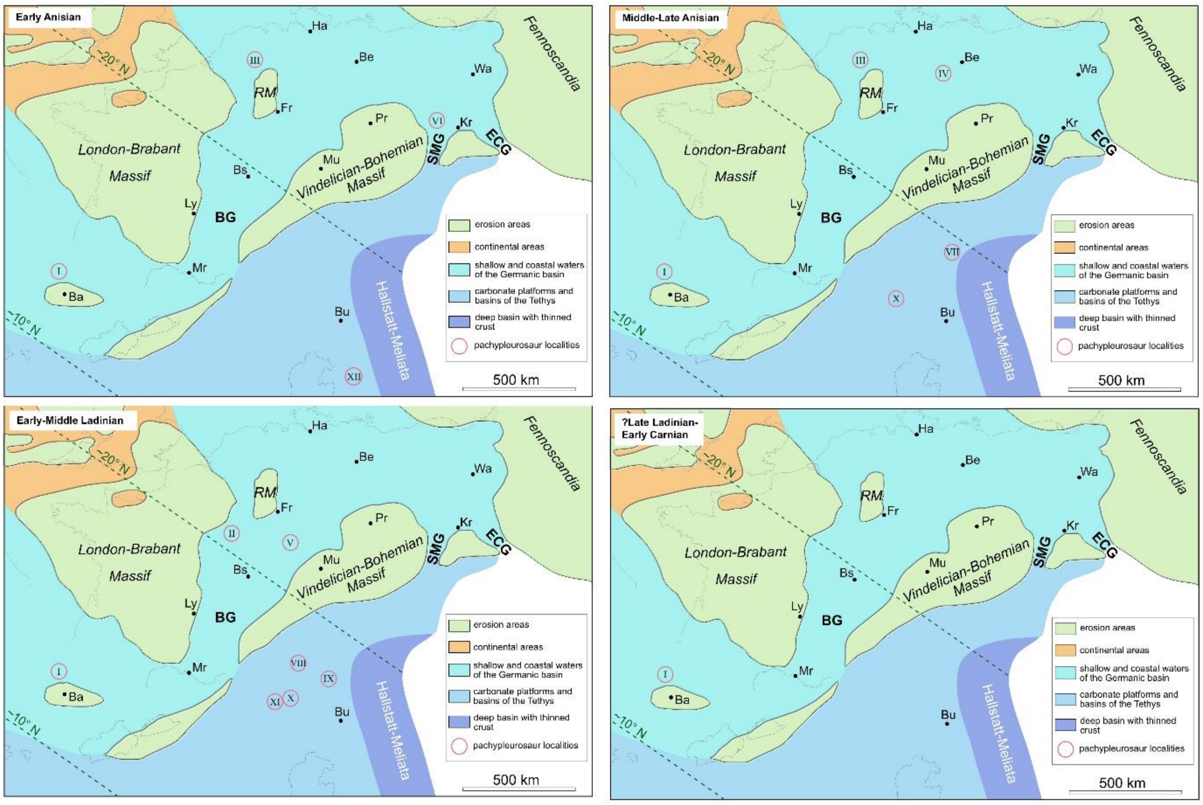

Fig. S28. Cladogram resulting from Traditional Search (TNT Version 1.5; settings: 100000 replications, 1000 trees held per replication, TBR active, outgroup *Simosaurus*; Memory settings: 30000 trees, 1000MB) with the more complete specimens from the Prosanto Formation treated as single OTUs. Tree length steps 186. Strict consensus of 3 trees.

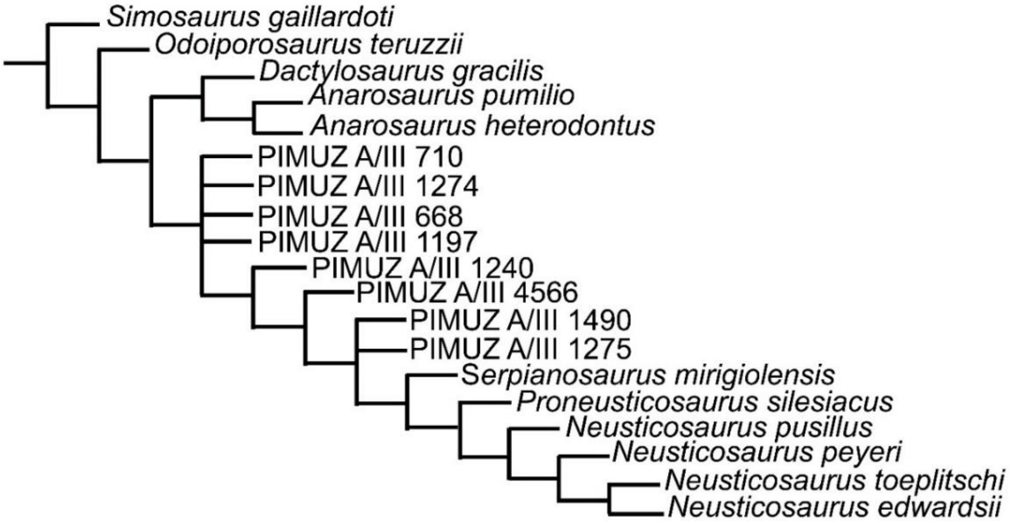

Fig. S29. Cladogram resulting from New Technology Search (TNT Version 1.5; default settings) with the more complete specimens from the Prosanto Formation treated as single OTUs. Tree length steps 186. Strict consensus of 3 trees.

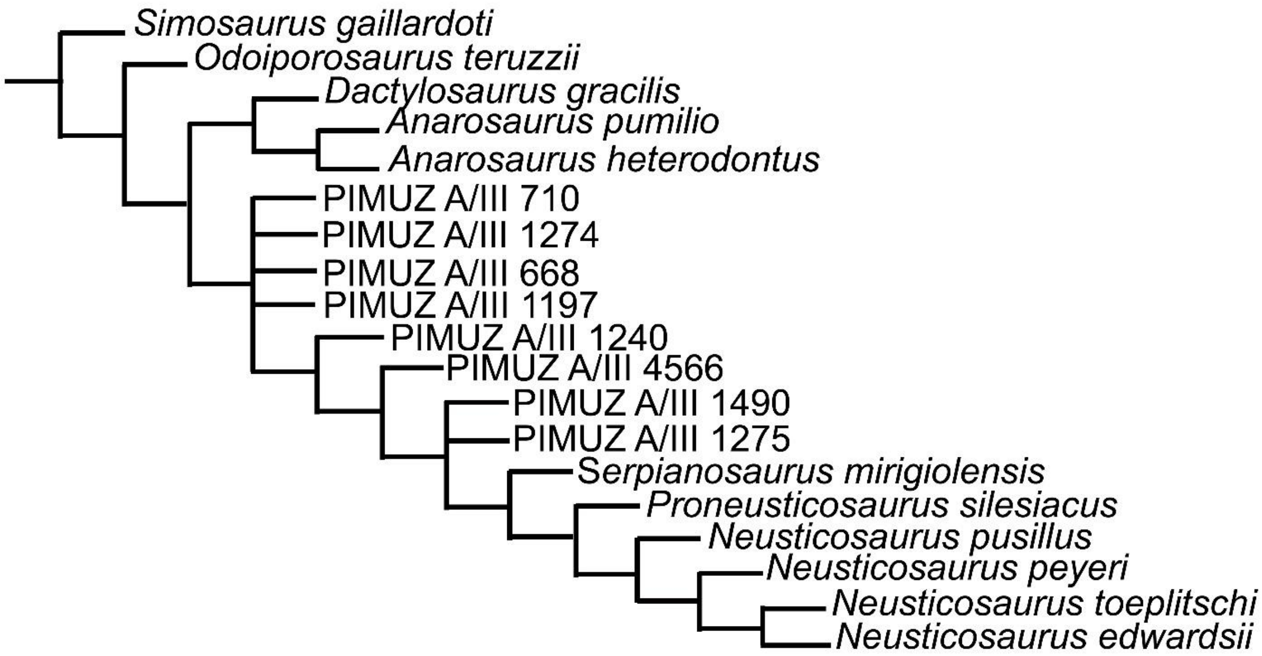

Fig. S30. Cladogram resulting from Traditional Search (TNT Version 1.5; settings: 100000 replications, 1000 trees held per replication, TBR active, outgroup *Simosaurus*; Memory settings: 30000 trees, 1000MB) with the eight specimens from the Prosanto Formation treated as a single OUT. Tree length steps 178. Strict consensus of 1 tree.

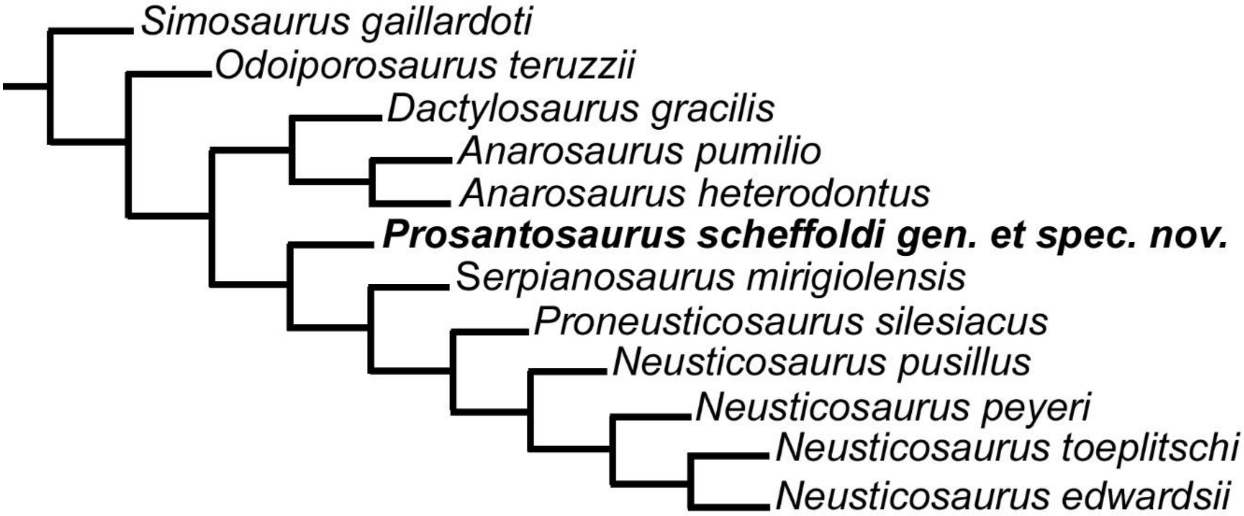

Fig. S31. Cladogram resulting from New Technology Search (TNT Version 1.5; default settings) with the eight specimens from the Prosanto Formation treated as a single OTU. Tree length steps 178. Strict consensus of 1 tree.

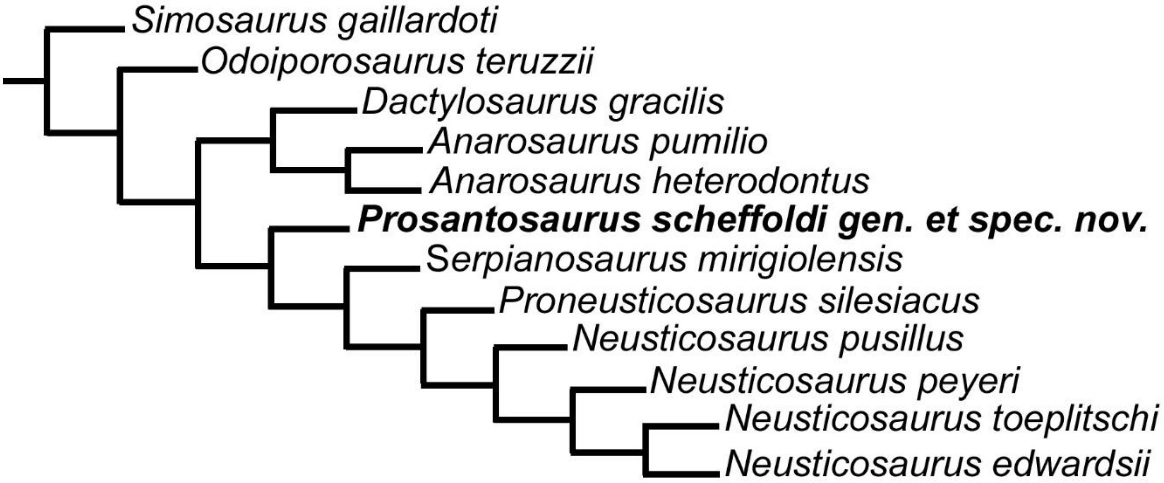

II. Character matrix

Character 1

Nasals sculptured (0) or smooth (1).

Modified character (1) from Rieppel and Lin 1995.

Sculpture includes any striation, grooves etc.

Character 2

Frontals sculptured (0) or smooth (1).

Modified character (1) from Rieppel and Lin 1995

Sculpture includes any striation, grooves etc.

Character 3 [NEW CHARACTER]

Skull shape: broadest at height of orbitae (0); parallel margins (1); wedge-shape (widest at posterior orbit/skull table) (2).

Character 4

Nasals in broad contact medially (0); median contact of nasals reduced (1); Nasals completely separated (2). Nasals completely separated and reduced (3).

Modified from character 4 from Rieppel and Lin (1995).

Character 5

Nasals broad and leaf-like (0); triradiate (1); small elongated (2); reduced (3).

Character 3 from Rieppel and Lin (1995).

Character 6

Prefrontal protruded/bulging (0); reduced (1).

Modified from Character 8 from Rieppel (2001).

Autapomorphy of *Simosaurus* (1), rest (0)

Character 7 [NEW CHARACTER]

Prefrontal and nasal are separated (0) or have an anterolateral contact (1).

Character 8 [NEW CHARACTER]

Maxilla extends far posterior almost to or up to posterior margin of orbit (0); reaches up to the mid orbit region (1); ends ate the anterior orbit (2).

Character 9

Frontals paired (0); fused in the adult (1).

Character 5 from Rieppel and Lin (1995).

Character 10

Frontal(s) without (0); or with distinct anterolateral process(es) entering between the prefrontal and the nasal (1).

Character 6 from Rieppel and Lin (1995).

Character 11

Nasals in broad contract at midline of skull (0), nasals meet in a short suture only (1), or nasals are separated by the contact of the nasal process of the premaxilla with the frontal (2).

Modified character 4 from Rieppel and Lin (1995).

Character 12

Premaxilla excluded from contact with the external and internal nares (0) or in contact (1). The premaxilla is excluded from the external and internal nares in the new taxon.

In all other European pachypleurosaurs, the premaxillae enter always somehow the external and internal nares, independent of preservation.

Modified character 19 from Rieppel and Lin (1995).

Character 13

Frontal broadly enters the dorsal margin of the orbit (0); participation of the frontal in the dorsal margin of the orbit is restricted due to an elongated anterior process of the postfrontal and/or due to an elongated posterior process of the prefrontal (1).

Modified character 7 from Rieppel and Lin (1995).

Character 14

Posterior process of frontal widely separated from the upper temporal fossa (0); narrowly approaches the upper temporal fossa (1); enters the anteromedial margin of the upper temporal fossa (2).

Character 29 from Li and Liu (2020).

Character 15

Lateral edge of frontal concave (0); straight (1).

Character 8 from Rieppel and Lin (1995).

Character 16 [NEW CHARACTER]

A ventral process of the postfrontal encompasses the dorsal tip of the postorbital at the dorsoposterior orbit margin (0); the postfrontal has no ventral process (1).

Character 17 [NEW CHARACTER]

The anteromedial process of the postorbital overlying the postfrontal is broad and rounded (0) or is not well developed or distinctly pointed (1).

Character 18 [NEW CHARACTER]

Anteromedial process of the postorbital articulates on the postfrontal anterolaterally (0) or contributes to the posterior orbit margin (1).

Character 19 [NEW CHARACTER]

Postorbital contributes to upper temp. margin (0); forms most of lateral margin (1); excluded (2).

Character 20

Width of upper temporal arch, i.e., temporal emargination, visible from above (0); not visible from above (1).

Character 11 from Rieppel and Lin (1995).

Contrary to Renesto et al. (2014) we coded *Odoiporosaurus* as 0.

Character 21

Posterior part of skull margin deeply concave/emarginated (i.e., posterior squamosals form wide angle) (0); slightly emarginated (1); not emarginated/straight (2).

Modified character 10 of Rieppel and Lin (1995).

Character 22

Parietal paired (0); fused (1) in adult.

Character 12 from Rieppel and Lin (1995).

Character 23 [NEW CHARACTER]

Parietal longer (posterior skull margin) than width (0); equal (1), wider than long (2).

Character 24

Pineal foramen located in centre of skull table (0); displaced anteriorly (1); displaced posteriorly (2).

Character 13 from Rieppel and Lin (1995).

Character 25

Supraoccipital, sagittal crest is absent (0); reduced (1); prominent (2).

Modified Character 66 from Li and Liu (2020).

Character 26

Shape o upper temporal opening: elongated oval (0); round oval (1).

Modified character 9 of Rieppel and Lin (1995)

Character 27

Size of upper temporal opening reduced (0); "pachy normal" (1); enlarged (2); distinctly enlarged (3).

Modified character 9 of Rieppel and Lin (1995)

Character 28 [NEW CHARACTER]

Posterior part of lower jaw normal (0); or massive (1).

Character 29

Mandibular articulations: approximately at level with occipital condyle (0); displaced to a level distinctly behind occipital condyle (1).

Character 51 from Lin et al. (2021).

Character 30

Premaxillary and anterior dentary teeth small (0); enlarged (1).

Character 24 from Rieppel and Lin (1995).

Character 31

Maxillary tooth row restricted to anterior margin of orbit (0); maxillary tooth row reaches til the mid orbit (1); extending to or over the posterior orbit (2).

Modified Character 26 from Rieppel and Lin (1995).

Character 32

Number of (visible) teeth upper jaw <15 (0); 16 - 20 (1); > 21 (2).

Character 33

Number of teeth lower jaw <15 (0); 16 - 25 (1); > 26 (2).

Character 34

Functional premaxillary teeth 4 (0); 5-6 (1); more than 6 (2).

Modiefied from character 93 from Li and Liu (2020).

Character 35

Premaxillary fangs absent (0); present (1).

Modified from Character 94 from Li and Liu (2020).

Character 36

One or two enlarged teeth on maxilla present (0); absent (1).

Character 95 from Li and Liu (2020).

Character 37 [NEW CHARACTER]

Posterior pterygoid strongly constricted (entire element not only shelf) with massive lateral processes connected to a massive quadrate (0); posterior pterygoid strongly constricted without massive lateral processes (1); mid-part of pterygoid is constricted (2); no distinct constriction (3).

Postcranial skeleton

Morphology

Character 38

In ventral view dorsal centra constricted (0); unconstricted (1).

Character 28 from Rieppel and Lin (1995).

Character 39

Neural spines on dorsal vertebrae in adults: high [higher than neural arch] (0); low/neural spine lower than neural arch (1).

Modified character 5 from Sander (1989)

Character 40 [NEW CHATRACTER]

Shape of neural arch of dorsals in dorsal view pentagonal [distinctly constricted margin between pre- and postzygapophyses of each side] (0); rectangular (1).

Character 41

Dorsal ribs without pachyostosis (0); with pachyostosis (1).

Character 31 from Rieppel and Lin (1995).

Character 42

Distal expansion of the sacral ribs smaller than proximally (0); equal in size (1).

Modified character 33 from Rieppel and Lin (1995).

Character 43

Clavicles without anterolaterally expanded corners (rounded shape) (0); with expanded corners (rectangular shape) (1).

Modified character 35 from Rieppel and Lin (1995).

Character 44

Clavicles: broad medially (0); narrow/tapering medially (1).

Character 77 from Rieppel et al. (2002).

Character 45

Posterior stem on interclavicle distinct (0); rudimentary (1); absent (2).

Character 36 from Rieppel and Lin (1995).

Character 46

Shape of interclavicle rhomboidal (0); T-shaped (1); triangular (2).

Character 135 from Li and Liu (2020).

Character 47

Proximal head of humerus in adults clearly set off/angled (0); smooth transition (1); no distinct transition (2).

Modified Character 146 from Li and Liu (2020).

Character 48

Humerus shaft constricted (0); not distinctly constricted (1).

Modified Character 147 from Li and Liu (2020).

Character 49

Ectepicondylar groove on humerus notched anteriorly (0); without notch (1).

Character 37 from Rieppel and Lin (1995).

Character 50

Distinct entepicondyle on the humerus present (0); absent (1).

Character 38 from Rieppel and Lin (1995).

Character 51

Entepicondylar foramen present (0); absent (1).

Character 39 from Rieppel and Lin (1995).

Character 52

Epicondyles of humerus prominent (0); reduced (1).

Character 150 from Li and Liu (2020).

Character 53 [NEW CHARACTER]

Humerus osteosclerotic (0); strongly osteoslcerotic (1).

Character 54 [NEW CHARACTER]

Humerus low growth rates (lb/pfb low to moderate vascu) (0); increased/high growth rates [less orga tissue, higher vascu] (1).

Character 55

Deltopectoral crest present/well developed (0); reduced or absent (1).

Character 148 from Li and Liu (2020).

Character 56

Ulna, mid-diaphysis/shaft: slender/constricted (0); broadened (1).

Character 41 from Rieppel and Lin (1995).

Character 57

Ulna: distal end not expanded, narrower than proximal part (0); prox and distal approx. equally sized (1); proximal part expanded (2); distal part expanded (3).

Modified character 126 from Rieppel et al. (2002).

Character 58 [NEW CHARACTER]

Ulna asymmetrical (curved) (0); symmetrical (1).

Character 59

Radius shorter than ulna (0), radius slightly longer than ulna (1), or bones of approximately equal length (2).

Character of 42 Rieppel and Lin (1995).

Character 60

Pubis without (0); or with (1) anteroventral process.

Character 49 from Rieppel and Lin (1995).

We coded Odopoisaurus as (0) contrary Renesto et al. (2014)

Character 61

Obturator foramen closed (0); open (1) in adults.

Character 50 from Rieppel and Lin (1995).

Character 62

Femoral **shaft**: straight (0); sigmoidally curved (1).
Modified Character 131 from Lin et al. (2021).

Character 63
Femoral **shaft**: stout (0); slender (1).
Modified Character 131 from Lin et al. (2021).

Character 64 [NEW CHARACTER]
Proximal part of femur in dorsoventral view equally size to distal part or slightly wider (0); more massive than the distal part (1).

Character 65
Proximal concavity of astragalus: absent (0); present (1).
Character 176 from Li and Liu (2020)

Numbers and ratios
Character 66
Number of cervical vertebrae: 15 or less (0); 16 -20(1); 21 or more (2).
Modified from character 106 from Li and Liu (2020).

Character 67
Number of dorsal vertebrae: 20 or below (0); 20 or more (1); 30 or more (2).
Character 30 from Rieppel and Lin (1995).

Character 68 [NEW CHARACTER]
Number of presacrals: less than 40 (0); more than 40 (1); more than 60 (2).

Character 69
Number of sacral vertebrae: two (0); three (1); four or more (2).
Character 32 from Rieppel and Lin (1995).

Character 70
Gastral ribs composed of five segments (0); three segments (1).
Character 14 from Sander (1989) and 34 from Rieppel and Lin (1995).

Character 71

Number of ossified carpal bones in adult: more than three (0); three (1); two (2).

Character 45 from Rieppel and Lin (1995).

Character 72

Number of tarsal ossifications: ≥ 4 (0); 3 (1); 2 (2).

Character 115 from Rieppel et al. (2002).

Character 73

Phalangeal formula of manus 2-3-4-5-3 (0) or characterised by hyperphalangy (1).

Modified character 46 from Rieppel and Lin (1995).

Character 74

Phalangeal formula in pes 2-3-4-5-3 (0) or less than 2-3-4-4-3 (1).

Character 47 from Rieppel and Lin (1995).

Character 75 [NEW CHARACTER]

Ratio, (post. squamosal) skull length/orbit length: < 0.20 (0), > 0.21-0.3 (1); > 0,31 (2).

Skull length/post. squamosal length (not lower jaw) vs. maximal longitudinal diameter of orbit).

Character 76 [NEW CHARACTER]

Preorbital and postorbital region of skull of subequal length (0); preorbital region distinctly longer (1); postorbital region distinctly longer (2).

Preorbital = measured from tip of snout to anterior margin of orbit

Postorbital = posterior margin of orbit to **posterior squamosal**

Character 12 from Rieppel et al. (2002) and character 2 Liu et al. (2011).

Character 77

Longitudinal diameter of the upper temporal fossa less than 30% (0); between 31% and 45% of orbital diameter (1); over 46% of orbital diameter (2).

Modified character 9 from Rieppel and Lin (1995).

Character 78

Ratio, post squamosal length/ skull length divided by longitudinal diameter of upper temporal fossa less than 5 (0); 6-10 (1); 11-20 (2); > 21 (3).

Modified from character 3 from Li and Liu (2020).

Character 79

Humerus shorter than femur (0), or approximately of same length (1); humerus longer than femur (2).

Modified *Character 111 from Lin et al. (2021).*

Character 80 [NEW CHARACTER]

Ratio, Humerus/femur < 1 (0); 1 to 1.5 (1); > 1.6 (2).

Character 81

Femur length/trunk length ratio > 0.2 (0); < 0.2 (1).

Modified character 11 from Sander (1989).

Character 82

Neck less than 80% of trunk length (0); between 80% and 100% of trunk length (1); distinctly more than 100% of trunk length.

Character 29 from Rieppel and Lin (1995).

III. Data matrix

```
Klein et al. Supplementary file II _Matrix_txt - Editor
Datei   Bearbeiten   Format   Ansicht   Hilfe
xread
82 22
Simosaurus      112331111121021110101100013010211101300001100100111101002020011012221011000220211?
Anarosaurus_heterodontus        01000010010111?00010000011111112211030100011[1 2][0 1][0 1]001000100001??01001112010??01120010
Anarosaurus_pumilio     ?100200001011001111000001110101??2???1100?????000100??0????11010?111?0????11110000
Dactylosaurus_gracilis  1100201001011001111000012020101??????1100111??000000100010100???01001011001021???1
Serpianosaurus_mirogil. 110120121111010111[0 2]0102001001012220011010101[1 2]0[0 1]1110[0 1]11[0 1]001[0 1]11010111[0 1]10220011031?00
Neusticosaurus_pusillus 1112201211[1 2]1010111[0 2]0100020100001110021111101[1 2]0[0 1]1[0 1][0 1]0[0 1]10[0 1]01110101012111122001102[1 2][0 1][0 1][0 1]
Neusticosaurus_peyeri   1122201211[1 2]1010000[0 2]1202220100001110021011101[1 2]0[0 1]1[0 1][0 1]0[0 1]10[0 1]01110[0 1]010110011220021020[0 1]00
Neusticosaurus_edwardsii        [0 1][0 1]12101211[1 2]100[0 1]000[0 2]1102121100001220031001101[1 2]010[0 1][0 1]0[0 1]11[0 1]011101001110011120111022[1 2][0 1]1
Odoiporosaurus  100210110121110111001011?02?1010?000?0100?????001100??012010001?1????0????111121??
Neusticosaurus_staubi   ??????????????????????????????????????001????201101??00111??010????1?1?0?????00??
Neusticosaurus_toeplitschi      ??????????????????????????????????????1111101??201101??101?10101110001?1100????001?
Proneusticosaurus       ??????????????????????????????????????11110???????????????31001110????200???????????
Keichousaurus   1103301010210000101021012030112101103000110011[1 2]1111110[0 1]1201001100201[0 1]00[1 2]102020[0 2][0 1][0 1]2
PIMUZ_1275      ???????????0????????????????00???????01????0?20111101??101110?0111111?02200????0000
cast    ???????????0????????????????00???????01????0120101101??101110?0101111?02200????0000
PIMUZ_1240      ??0????????????????100?00?0100?????????10010???001101??101110?01111111?2200????2110
PIMUZ_1197      110????0???0????????0????????000211100??100??120001101??00111?10101?1???2200????21??
PIMUZ_668       11022010002001100010000020100002?????????01?1??001101??00111??010??111?2???10112110
PIMUZ_1274      110220100020011000100000201000021?100??100101??001101??00111??010?1111?2???10122110
Prosanto        11022010002001100010000020100002[0 1]11000110010120[0 1][0 1]1101??[0 1]01110101[0 1]1111102200101[1 2][0 2][0 1][0 1]0
PIMUZ_1490      ???????????0?????????0?0???????20????0?????????111?0????0111???????????????????????
PIMUZ_710       ??02201000?001100010000020100?2??????????????????????????????????????????????????????
;


proc /;
comments 0
;
```
